# Transforming US agriculture for carbon removal with enhanced weathering

**DOI:** 10.1038/s41586-024-08429-2

**Published:** 2025-02-05

**Authors:** David J. Beerling, Euripides P. Kantzas, Mark R. Lomas, Lyla L. Taylor, Shuang Zhang, Yoshiki Kanzaki, Rafael M. Eufrasio, Phil Renforth, Jean-Francois Mecure, Hector Pollitt, Philip B. Holden, Neil R. Edwards, Lenny Koh, Dimitar Z. Epihov, Adam Wolf, James E. Hansen, Steven A. Banwart, Nick F. Pidgeon, Christopher T. Reinhard, Noah J. Planavsky, Maria Val Martin

**Affiliations:** 1 Leverhulme Centre for Climate Change Mitigation, School of Biosciences, University of Sheffield, Sheffield, UK; 2 Department of Oceanography, Texas A&M University, College Station, TX USA; 3 School of Earth and Atmospheric Sciences, Georgia Institute of Technology, Atlanta, GA USA; 4 Advanced Resource Efficiency Centre, Management School, University of Sheffield, Sheffield, UK; 5 School of Engineering and Physical Sciences, Heriot-Watt University, Edinburgh Campus, Edinburgh, UK; 6 Exeter Business School, University of Exeter, Exeter, UK; 7 Cambridge Centre for Energy, Environment and Natural Resource Governance, The David Attenborough Building, Pembroke Street, University of Cambridge, Cambridge, UK; 8 World Bank, Washington DC, USA; 9 Environment, Earth and Ecosystems, The Open University, Milton Keynes, UK; 10 Department of Ecology and Evolutionary Biology, Princeton University, Princeton, NJ USA; 11 Earth Institute, Columbia University, New York, NY USA; 12 School of Earth and Environment, University of Leeds, Leeds, UK; 13 Understanding Risk Research Group, School of Psychology, Cardiff University, Cardiff, UK; 14 Department of Earth and Planetary Sciences, Yale University, New Haven, CT USA

**Keywords:** Carbon cycle, Climate and Earth system modelling

## Abstract

Enhanced weathering (EW) with agriculture uses crushed silicate rocks to drive carbon dioxide removal (CDR)^1,2^. If widely adopted on farmlands, it could help achieve net-zero emissions by 2050^2–4^. Here we show, with a detailed US state-specific carbon cycle analysis constrained by resource provision, that EW deployed on agricultural land could sequester 0.16–0.30 GtCO_2_ yr^−1^ by 2050, rising to 0.25–0.49 GtCO_2_ yr^−1^ by 2070. Geochemical assessment of rivers and oceans suggests effective transport of dissolved products from EW from soils, offering CDR on intergenerational timescales. Our analysis further indicates that EW may temporarily help lower ground-level ozone and concentrations of secondary aerosols in agricultural regions. Geospatially mapped CDR costs show heterogeneity across the USA, reflecting a combination of cropland distance from basalt source regions, timing of EW deployment and evolving CDR rates. CDR costs are highest in the first two decades before declining to about US$100–150 tCO_2_^−1^ by 2050, including for states that contribute most to total national CDR. Although EW cannot be a substitute for emission reductions, our assessment strengthens the case for EW as an overlooked practical innovation for helping the USA meet net-zero 2050 goals^5,6^. Public awareness of EW and equity impacts of EW deployment across the USA require further exploration^7,8^ and we note that mobilizing an EW industry at the necessary scale could take decades.

## Abstract (web summary)

A state-level analysis of the impact of enhanced weathering deployment on carbon sequestration on agricultural land suggests that enhanced weathering could help the USA meet net-zero 2050 goals.

## Main

The US strategy to reach net-zero greenhouse gas (GHG) emissions by 2050 includes decarbonizing the energy system and deployment of carbon dioxide removal (CDR) technologies at scale^5,6^ to sequester a billion metric tons of CO_2_ annually (1 GtCO_2_ yr^−1^) within three decades. CDR strategies are needed to achieve a net-zero carbon budget which is otherwise unlikely given hard-to-decarbonize industries such as agriculture and aviation. Potential pathways to meet the US CDR goal focus on enhancing natural land carbon sinks and scaling up CO_2_ removal technologies, mainly bioenergy with carbon capture and storage (BECCS) and direct air capture with carbon storage (DACCS); see ref. ^5^. Given evidence that current GHG concentrations are already well into the dangerous zone^9^, there is a possibility that even greater CDR will be needed to achieve a negative carbon budget over the coming century.

Here we focus on the potential of purposeful terrestrial enhanced weathering (EW) of rock in agricultural settings as a promising but still underexplored CDR technology for meeting US decarbonization targets^5,6^. The Corn Belt in the American Midwest alone has more than 70 million ha in corn and soybean rotation and represents one of the most intensively managed agricultural regions in the world. With existing infrastructure, acidic soils, suitable crops and a large area, this region provides ideal opportunities for integrating EW practices^3,4,10^.

**Extended Data Fig. 1:**
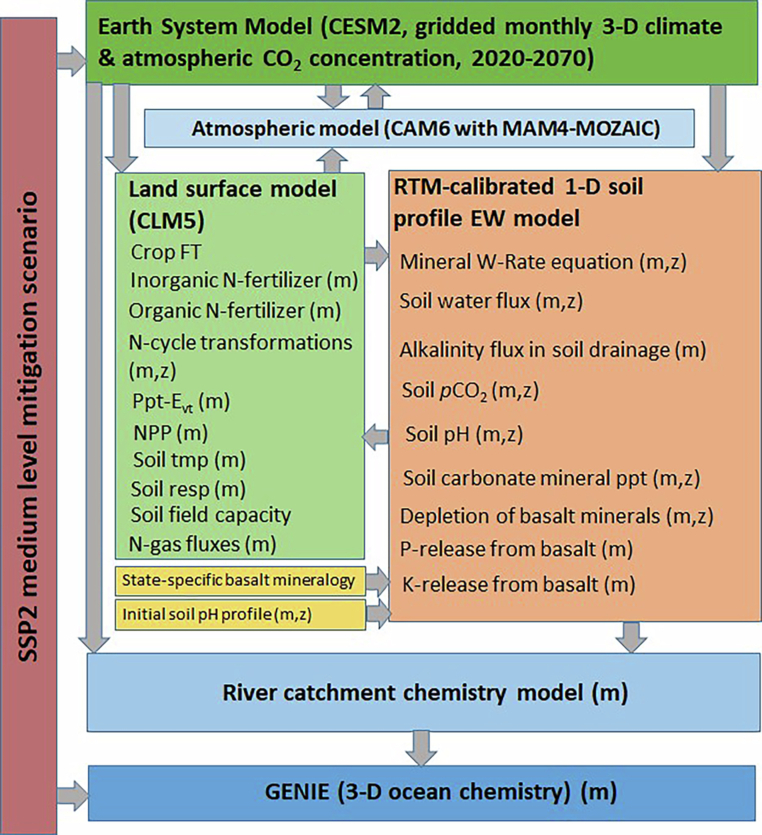
Schematic diagram of the Earth system modelling assessment of enhanced rock weathering deployment in the U.S. m – monthly, z by depth.

**Extended Data Fig. 2:**
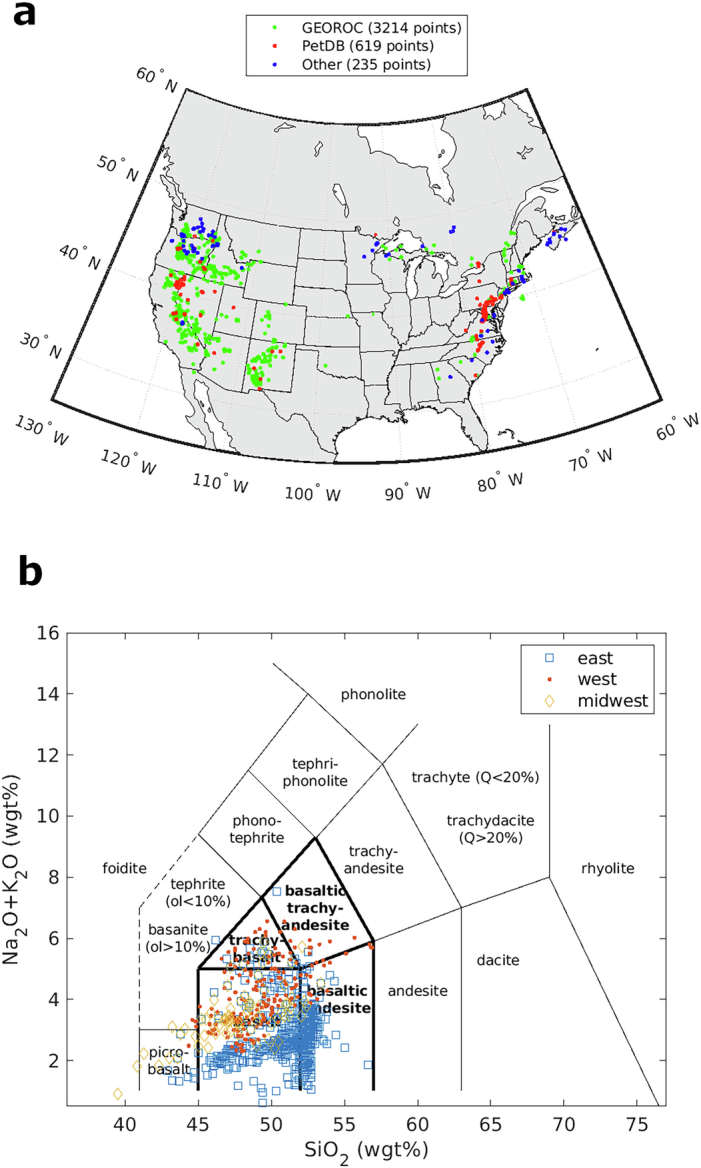
Rock geochemistry locations and classifications. (**a**) Geographical locations of geochemical data for U.S. mafic rocks (see Methods for databases, Data File 1). (**b**) Classification of mafic rock geochemistry based on sodium and potassium oxides vs silica oxides. Eastern points are east of -90E, western points are west of -100E, selected states are circled.

**Extended Data Fig. 3:**
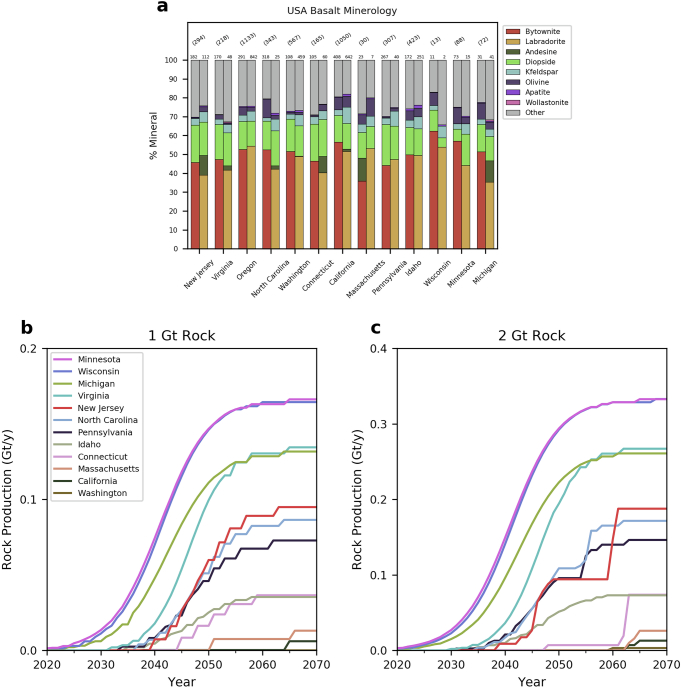
Representative state-specific normative minerologies and annual quarrying rates. (**a**) Representative basalt minerologies (two per state) using in the soil profile EW model simulations. Based on cluster analysis of normative mineralogies for mafic rocks within the geographical boundary of each state. Number above each bar is *n* data points assigned to each cluster and the number in brackets is the total data points for each state. Minerals included in the ‘other’ category are quartz, enstatite, ilmenite, magnetite, albite, oligoclase, anorthite. Modelled basalt quarrying rates by state for (**b**) the 1 Gt yr^−1^ and (**c**) the 2 Gt yr^−1^ extraction by 2070. In these modelled quarrying curves, Minnesota and Wisconsin ramp-up production early due to their close proximity to the Corn Belt. Michigan and Virginia follow, with Virginia lagging behind as it is further from crop areas than Michigan but ramping up production faster as it is already producing 50 Mt of crushed rock a year. Washington, Massachusetts, and California, produce small volumes of basalt to cover mostly in-state requirements due to their distance to the Corn Belt.

**Extended Data Fig. 4:**
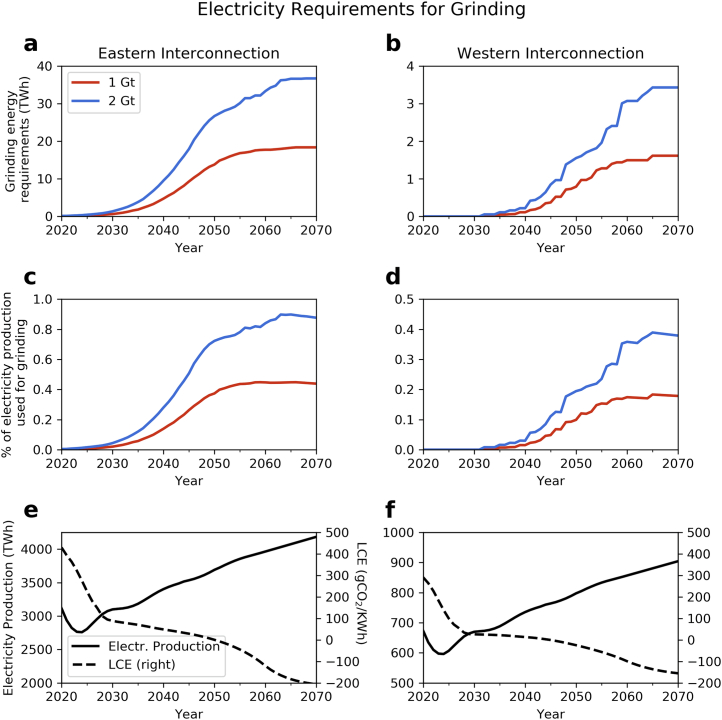
Energy requirements for rock grinding. Grinding requirements in electricity for eastern and western interconnections for 1 and 2 Gt rock yr^−1^ scenarios. (**a**, **b**) Annual electricity requirements for grinding in TWh/y. (**c**, **d**) Annual electricity requirements for grinding as a percentage of total electricity production. (**e**, **f**) Annual electricity production in TWh/y and Life Cycle Emissions (LCE) of electricity in gCO_2_/kWh.

We present an integrated whole-system assessment of feasibility, costs and the possible outcomes for soil and air quality of upscaling EW in future decades with US agriculture for CDR (Extended Data Fig. 1). State-specific low- and high-basalt supply rates, by means of quarrying, provide feedstock constraints (Extended Data Figs. 2 and 3 and Supplementary Figs. 1 and 2) for high-resolution dynamic offline climate–carbon–nitrogen cycle EW simulations^11^ (2020–2070) adopting the Shared Socioeconomic Pathway (SSP) 2 medium-level mitigation scenario (Extended Data Fig. 1 and Supplementary Figs. 3–6). Technological development pathways for EW are, however, constrained by basalt supply rate and regional future policies for decarbonization of energy and transportation systems in line with more stringent US net-zero pathways^12,13^ (Extended Data Fig. 4, Supplementary Figs. 7–10 and Supplementary Tables 1–3). We build on this analysis by assessing the effects of EW on regional air quality and soils, highlight key challenges of upscaling and discuss the requirement for social license to operate this technology at scale^7,8,14^.

## Carbon drawdown potential of US agriculture

**Fig. 1:**
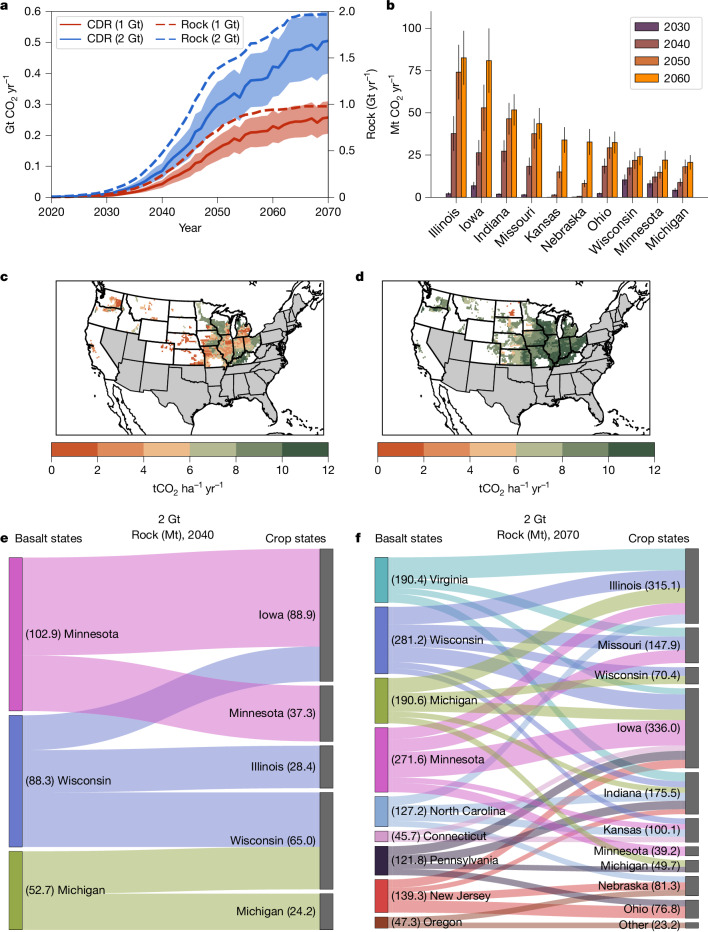
Atmospheric CDR by enhanced weathering with US agriculture. **a**, Net annual cumulative CDR by EW as constrained by 1 Gt yr^−1^ and 2 Gt yr^−1^ rock extraction scenarios, 2020–2070 (annual crushed basalt application of 40 t ha^−1^). The shaded area shows the 90% uncertainty envelope due to differences in the mineralogy of basalt sourced from the supply states. **b**, Mean (with 90% confidence limits) annual CDR rates of the top ten states (2 Gt rock yr^−1^ scenario). **c**,**d**, Spatial patterns of net annual CDR rates per hectare in 2040–2050 (**c**) and in 2060–2070 (**d**) for the 2 Gt rock yr^−1^ by 2070 scenario. All simulations are illustrative for crushed basalt with a particle size P80 of 100 µm, that is, 80% of particles less than or equal to 100 µm diameter; previous work indicates that particle size has a relatively minor effect on net CDR over decadal timescales^11^. All CDR figures are net and account for the CO_2_ emissions penalty associated with mining, grinding, transporting and distributing rock dust. **e**,**f**, Sankey diagrams illustrating the main transfer pathways of crushed rock from basalt source states to recipient cropland states in 2040 (**e**) and 2070 (**f**), for the 2 Gt yr^−1^ rock extraction by 2070 scenario; only fluxes greater than 20 Mt yr^−1^ are shown for clarity.

Simulation results indicate that EW deployed with crushed basalt applied annually (40 t ha^−1^) gives a net CDR potential for US agriculture of between 0.16 ± 0.04 GtCO_2_ yr^−1^ (90% confidence limits) and 0.30 ± 0.07 GtCO_2_ yr^−1^ by 2050 for our low and high rock extraction scenarios, respectively (Fig. 1a). The dominant source of uncertainty in these simulations is within and between state differences in basalt mineralogy. By 2070, the CDR potential of EW increases further to 0.25 ± 0.05 GtCO_2_ yr^−1^ and 0.49 ± 0.1 GtCO_2_ yr^−1^ for both scenarios, the latter being equivalent to about 6% of present US emissions, with 85–90% of this CDR delivered by ten of the 20 states analysed. For each state, the CDR potential rises over successive decades following repeated annual crushed rock applications (Fig. 1b). Of those ten states, four Corn Belt states (Illinois, Iowa, Indiana and Missouri) make the largest contributions, in part, by virtue of having a large geographical area for rock dust deployment reaching net 40–75 MtCO_2_ removal per year by 2050 (Fig. 1b). Three other Corn Belt states (Wisconsin, Minnesota and Michigan) with smaller deployment areas, achieve average CDR rates of 15–20 MtCO_2_ yr^−1^ by 2050 (Fig. 1b).

Geospatial patterns of CDR rates per hectare (Fig. 1c,d) primarily reflect the timing of when EW is initiated, which in our model is a function of proximity to basalt supply, soil pH, crop type and climate. We assume that supply states nearest to agricultural states respond quickest to demand, and that those states with existing infrastructure for rock extraction are best placed to achieve the highest production rates. Earlier EW deployment allows greater cumulative CDR as a result of consecutive annual applications, with basalt added in earlier years still capturing CO_2_ years later as the slow-weathering minerals dissolve^11^. Corn Belt states closest to basalt production states start EW early and have the highest potential CDR rates per hectare by 2070 (Fig. 1d). Optimal selection of states for basalt provision to croplands is based on minimizing transportation distances, thus maximizing CDR by keeping logistical CO_2_ emissions low (which also reduces overall cost) and delivering EW efficiently over time. This analysis identifies states to prioritize for early EW implementation to maximize CDR potential.

**Extended Data Fig. 5:**
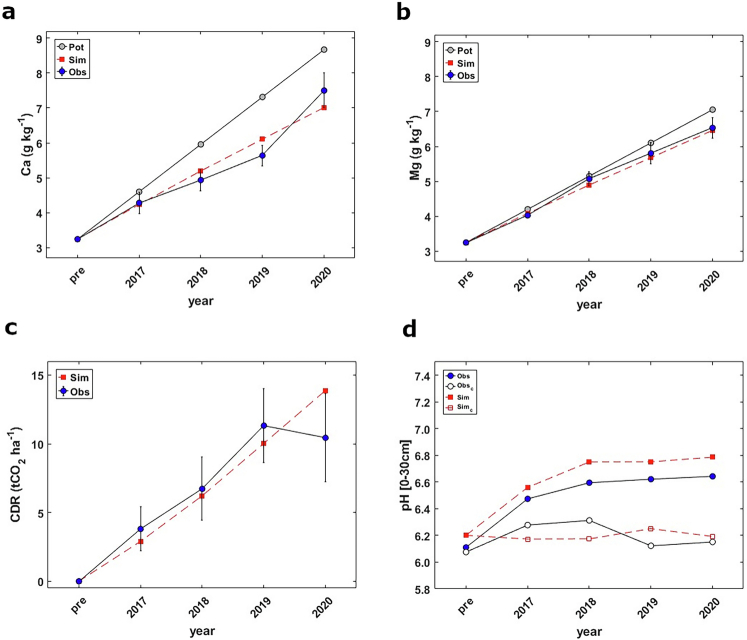
Comparison of observed and modelled EW rates in the U.S. Corn Belt. Observed (Obs)^4^ (mean ± s.e.m, *n* = 5) and simulated (Sim) rates of loss of (**a**) calcium (Ca^2+^), (**b**) magnesium (Mg^2+^) ions by EW at 0–20 cm soil depth by EW of crushed basalt. (**c**) Cumulative rate of potential carbon dioxide removal (CDR), based on mass balance. (**d**) Average depth-weighted treated (Obs) and control (Obs_c_) bulk soil pH (0–30 cm) for the Energy Farm EW field trial, Illinois^3^, with model predictions (for soil pore water) pH with (Sim) and without EW (Sim_c_); pH measurements are for bulk soil. 2016 is the pre-treatment year. Simulations were undertaken using the 1-D soil profile EW model with the blue ridge metabasalt mineralogy, particle size distribution, and application rate as used in the field trials, driven with CESM climate and CLM5 soil profile inputs (Extended Data Fig. 1), i.e., the same methodology as the current study. Pot in (a) and (b) indicates maximum potential soil Ca and Mg concentrations respectively, based on basalt chemistry and application rate, i.e. assuming no weathering^3^.

Comparison of modelled EW rates with field trial results from the Corn Belt^3^ indicates model agreement within error of observations for Ca^2+^ and Mg^2+^ loss and soil pH over 4 years, lending support to our approach (Extended Data Fig. 5). However, we recognize the limited nature of this initial single site model evaluation over several years and the need for more empirical data to better constrain field trial weathering rates, the effects of secondary mineral phase formation (for example, clays) and overall model uncertainty. We also need to better understand the time lag between weathering in soils and bicarbonate export in drainage waters due to cation sorption throughout the soil column. These represent continuing challenges for robust monitoring, reporting and verification (MRV) of CDR by EW^15^ (Table 1). Nonetheless, measured weathering rates at the Illinois EW field trial site indicate that rapid weathering is possible.

**Table 1:** Uncertainties and research priorities for assessing carbon removal by enhanced weathering with agriculture

Uncertainty	Research and development requirement
**Science**	
Effects of heterogonous soils (for example, pH, structure, hydrology and background mineralogy), crop functional types and climates on rates of mineral weathering over time.	Replicated multiyear field trials across a diversity of soils, crops and climates with detailed measurements of in situ rates of cation loss from feedstock.
Time lag between feedstock dissolution in surface soils and CDR through bicarbonate export and its dependency on background soil pH, fluid fluxes and soil cation exchange capacity.	Long-term watershed EW deployment sites with soil biogeochemistry (cation exchange complexes, exchangeable acidity, secondary mineral formation and base saturation), measurements of soil pore water and stream water chemistry monitoring. Numerical modelling of cation residence time in soil layers.
Potential and timescale for longer-term CO_2_ storage in groundwater.	Knowledge of hydrological flow paths, aquifer fluid dynamics, alkalinity and dissolved inorganic carbon and breakthrough times to streams.
Potential carbonate and clay formation in streams, rivers and groundwaters.	Modelling hydrological flow paths, aquifer fluid dynamics, alkalinity and dissolved inorganic carbon and breakthrough times to streams, carbonate and clay precipitation dynamics. Monitoring of streams in catchments with intensive EW deployments.
Whether or when upstream CO_2_ loss in river or stream systems will be compensated by CO_2_ uptake in the ocean by exported weathered cations.	State-of-the-art high-resolution river system and ocean modelling for the fate of EW products and ocean biogeochemistry.
**Monitoring, reporting and verification**	
Lack of international accreditation framework, measurement protocols and standards for data collection and sharing. Lack of international accreditation of models for data interpretation and interpolation.	Development of robust cost-effective and practical measurement protocols in consultation with stakeholders. Model intercomparison and agreement about benchmarking and performance metrics by the geochemical community. Open-source code, protocols and standards.
Lack of scientific consensus about MRV, allowable ranges for uncertainty and cost.	Accredited process-models optimized or benchmarked with field trial datasets. New computational tools modelling whole-system CDR over time for carbon credit attribution for use by stakeholders, including non-scientists and landowners.
**Policy and wider impacts**	
Robust evaluation of local and state-level community social and environmental impacts.	Local and state-specific assessment of energy, equity and environmental justice implications following Department of Energy Justice40 principles. Environmental monitoring of the plant–soil–water environment.
Lack of understanding of community and stakeholder needs and values.	Scientific evaluation of community needs and values, and link with the methodologies and data collected for life-cycle assessment and MRV. Sustained farmer engagement.
Prospects for coherent science-led governance and its effects on EW deployment at scale.	Federal and state-specific empirical engagement work to understand the social impacts of evolving governance landscape and near-term deployment on agricultural communities and stakeholders. Rolling evaluation of social licence to operate EW.
Actionable policy portfolios across states or regions, CDR strategies and mitigation instruments across sectors.	Integrated and engaged research on policy options, techno-economic dynamics and integrated system impacts.

Our simulation results highlight the potential for EW deployment with agriculture to contribute 16–30% of the CDR required from CO_2_ removal technologies by 2050. This represents a substantial contribution to near-term US net-zero pathways^5^. Relative to engineered and other terrestrial CDR options proposed for achieving net-zero, rates of CO_2_ removal by EW are competitive and warrant consideration for large-scale implementation. BECCS, for example, has an estimated US technical potential 0.36–0.63 GtCO_2_ yr^−1^ in 2040, after accounting for constraints of long-distance biomass and CO_2_ transport, regional CO_2_ storage and injection well capacities^16^. Afforestation/reforestation (0.25–0.6 GtCO_2_ yr^−1^), agricultural practices to increase soil carbon sequestration (0.25 GtCO_2_ yr^−1^) and reforestation of understocked timberlands (0.19 GtCO_2_ yr^−1^)^17^ have similar CDR potential in the USA to that modelled here for EW (0.16–0.30 GtCO_2_ yr^−1^ by 2050) but with large uncertainties in the permanence of CDR.

Analysis of crushed rock transfer between source states and recipient agricultural states for achieving these CDR trajectories shows that within two decades three states with pre-existing quarrying infrastructure co-located with basalt reserves (for example, Wisconsin, Minnesota and Michigan) are the main rock suppliers to adjacent farmland (2040) (Fig. 1e). By 2070, basalt supply for EW ramps up to include seven key states meeting the demand of 11 main crop states (Fig. 1f), with Virginia, North Carolina and Pennsylvania becoming additionally important. Implementation of EW over time requires interstate transportation networks with sufficient capacity for moving basalt from supply states to crop states as well as engagement of several stakeholders for producing, collecting and transporting crushed rock (Fig. 1f). However, cost-effective quarrying and transporting of material at scale are activities that society undertakes today, with much of the required technology, infrastructure and human capital already in place. In terms of energy requirement, we calculate a demand of 0.4% (20 TWh) and 0.2% (1.5 TWh) of electricity production of the eastern and western power grids, respectively, for undertaking EW with up to 1 Gt yr^−1^ of rock extracted by 2070 (Extended Data Fig. 4). These figures rise to 0.8% and 0.4% by 2070 for 2 Gt yr^−1^ of rock extraction, consistent with independent analyses for the EW in the USA^18^, and fall within the range of current national power usage for rock comminution processes (USA 0.4%, Canada 1.9%, South Africa 1.8% and Australia 1.5%, but the EW demand will be additional to existing power usage)^19^.

## Durability of EW carbon sequestration

**Fig. 2:**
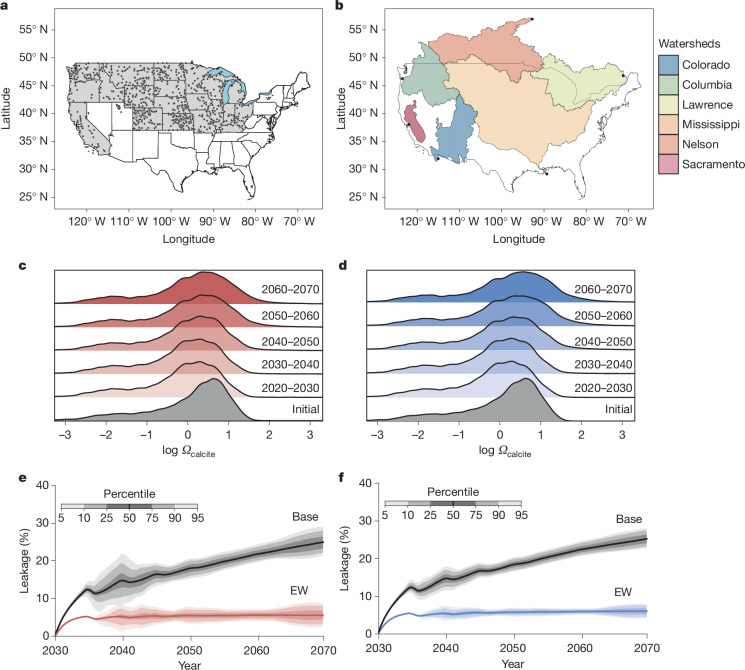
River and ocean responses to enhanced weathering. **a**, Locations of river and stream sites from the aqueous geochemical database used to estimate river calcium carbonate saturation states (*Ω*_calcite_). The filled circles show individual monitoring sites and the grey shading indicates the US states in which EW was applied. **b**, Watersheds over which river cation and dissolved inorganic carbon data were interpolated for use in our Earth system model. The shaded polygons show the watershed extent and the filled circles show the outflow locations. The six large watersheds considered are the Mississippi, Colorado, Columbia, Sacramento, Lawrence and Nelson. **c**,**d**, Ridgeline plots of *Ω* (log_10_) for US river systems in the background state (grey) and for each decade between 2020 and 2070 for the 1 Gt yr^−1^ (**c**) and 2 Gt yr^−1^ (**d**) rock extraction scenarios by 2070. **e**,**f**, Carbon leakage from the ocean during EW for the 1 Gt yr^−1^ rock extraction scenario (**e**) and 2 Gt yr^−1^ rock extraction scenario (**f**) by 2070. The solid lines and shaded regions show the median and percentile values, respectively, for our 984-member model ensemble. Base leakage refers to CO_2_ outgassing from the ocean; EW is the residual re-release of CO_2_ captured through EW.

We next quantify durability of CDR with EW by assessing the capacity of US rivers to carry dissolved EW products from soil drainage without extensive release of CO_2_ captured by EW through carbonate system equilibration^20^. Our analysis uses the major US river watershed water chemistry and flow data for 863 river sites (Fig. 2a,b) to calculate changes in the flow and carbonate saturation state (*Ω*) of river systems, 2020–2070 (Supplementary Figs. 11–14).

Simulation results show that with excess amounts of solutes (for example, Ca, Mg, Na, K and HCO_3_^−^) derived from EW entering river systems, *Ω* remains generally below the kinetic threshold required for extensive carbonate precipitation; that is, *Ω* of less than 10, for the 1 and 2 Gt yr^−1^ rock extraction scenarios (Fig. 2c,d). River *Ω* values increase with time as a result of increasing EW solute fluxes, but more than 86% of rivers across both scenarios have *Ω* values less than 10 by 2060–2070 (Fig. 2c,d), indicating that most have capacity to transport weathered products without extensive CO_2_ re-release^20^. Additionally, there are likely to be high rates of carbonate dissolution in the upper portion of riverine sediments—even in limited cases of water column carbonate formation^21^. Therefore, our river geochemistry calculations indicate that transport of dissolved constituents in surface waters is unlikely to be a limiting factor in the CDR potential of EW^20^.

**Extended Data Fig. 6:**
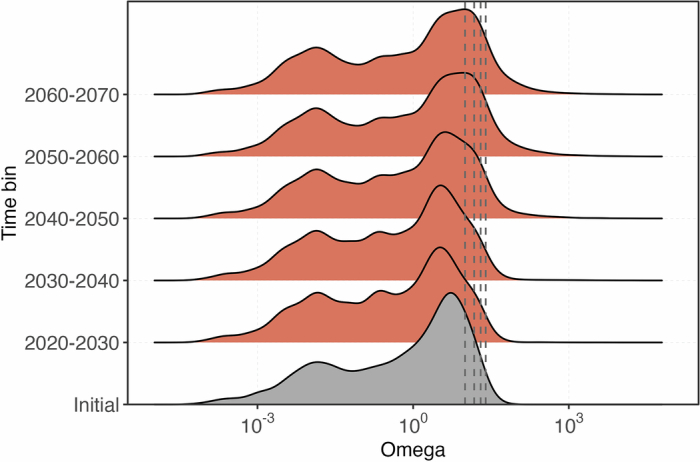
Ridgeline plots of the calcite saturation (omega) responses to EW over time for small catchments. Modelled calcite saturation (omega) for small USGS watersheds (<500 km^2^) for 2 Gt rock extraction by 2070 EW scenario. Vertical dashed lines indicate mean omega values of 10, 15, 20, 25 from left to right.

Effects of the influx of EW products from soils to rivers on *Ω* values may depend on spatial scale, with smaller scales (low-order rivers and tributaries) being more susceptible to variations in *Ω* values than larger river systems. However, smaller catchments (less than 500 km^2^) have a correspondingly smaller footprint of agricultural land and a proportionally lower flux of EW drainage products, with more than 85% still having *Ω* values less than 10 by 2060–2070 (Extended Data Fig. 6).

Our watershed river chemistry simulations assume no transformation or loss of ions through plant uptake and harvest, which would otherwise reduce the available alkalinity to the aquatic system. Similarly, cation exchange and secondary mineral formation in soils, which should act to decrease and prolong the flux of weathering products over time, are not considered. These assumptions render our river analysis a conservative estimate for the limit of EW solute transport. The impacts of these processes on the timing of solute transfer through stream drainage networks are an important topic for future work.

We calculate potential for CO_2_ leakage following the transport of EW products by rivers to the ocean with a three-dimensional ocean biogeochemistry model at locations representing the outlets of the six major watersheds (Fig. 2b). Results indicate a compensatory ocean outgassing of CO_2_ with EW deployment due to equilibration of the ocean–atmosphere system that gradually increases over time to around 10% in 2040 and 25% in 2070^22^ (Fig. 2e,f). This is a well-established Earth system response with all CDR technologies and emissions reductions. However, we also calculate a further around 5% backflow of CO_2_ out of the ocean caused by the re-equilibration of the shallow ocean carbonate system that represents carbon captured by EW returning to the atmosphere on short timescales (Fig. 2e,f). Potential upstream losses of CO_2_ from stream/river degassing are not necessarily additive with shallow ocean losses, as these two processes may compensate for one another with respect to net CO_2_ storage efficiency. Verification of the effectiveness of coastal ocean carbon storage requires higher resolution ocean models for specific EW deployments moving forward. Nevertheless, our results support the effectiveness of ocean carbon storage when EW is deployed at scale.

## Soil biogeochemistry responses to EW

**Fig. 3:**
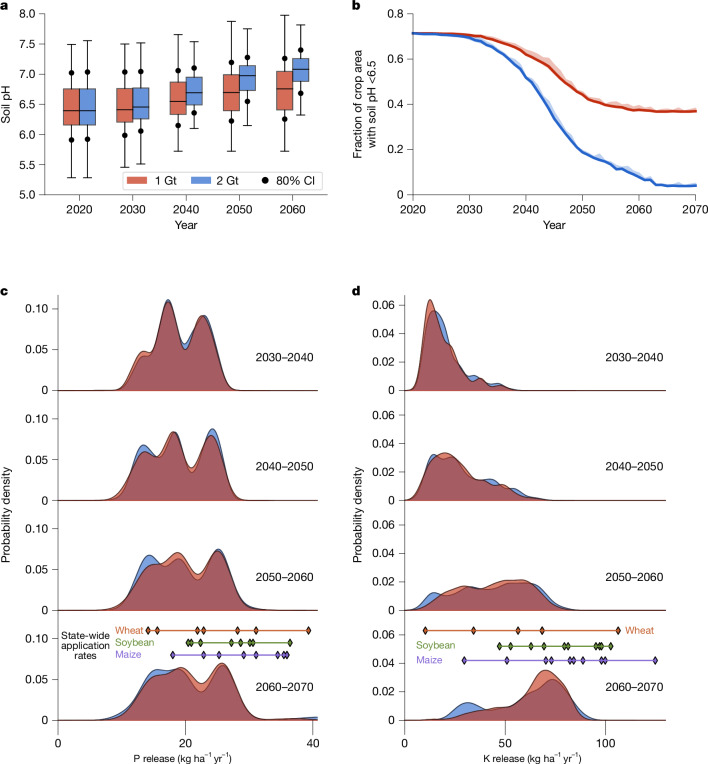
Benefits of enhanced weathering for agricultural soils. **a**, Boxplots with decadal average distributions of topsoil (0–15 cm depth) pH for grid cells representing the top ten Corn Belt states (defined by CDR potential), with EW deployment. Boxes show the interquartile range and median line, with whiskers extending to the 90% confidence interval. The dots depict the 80% confidence interval. **b**, Decreasing fraction of acidified lands in the same ten Corn Belt states over time with EW deployment. The shading denotes 90% confidence limits. A threshold pH value of 6.5 is used as this is beneficial for most arable crops and serves as a reasonable middle ground for safeguarding yields. Only a small fraction of grid cells will have an average pH value close to the 6.5 boundary value each year, resulting in a narrow confidence interval range. **c**,**d**, Frequency histograms of phosphorus (P) release (**c**) and potassium (K) release (**d**) by EW with basalt over successive decades for Corn Belt states (2030–2070). Also indicated in **c** and **d** are the application rates of P and K fertilizers for individual states growing soybean, maize and wheat. Simulation results for annual crushed basalt applications of 40 t ha^−1^. The red and blue in **a**–**d** indicate the 1 Gt yr^−1^ and 2 Gt yr^−1^ rock extraction by 2070 scenarios. CI, confidence interval.

**Extended Data Fig. 7:**
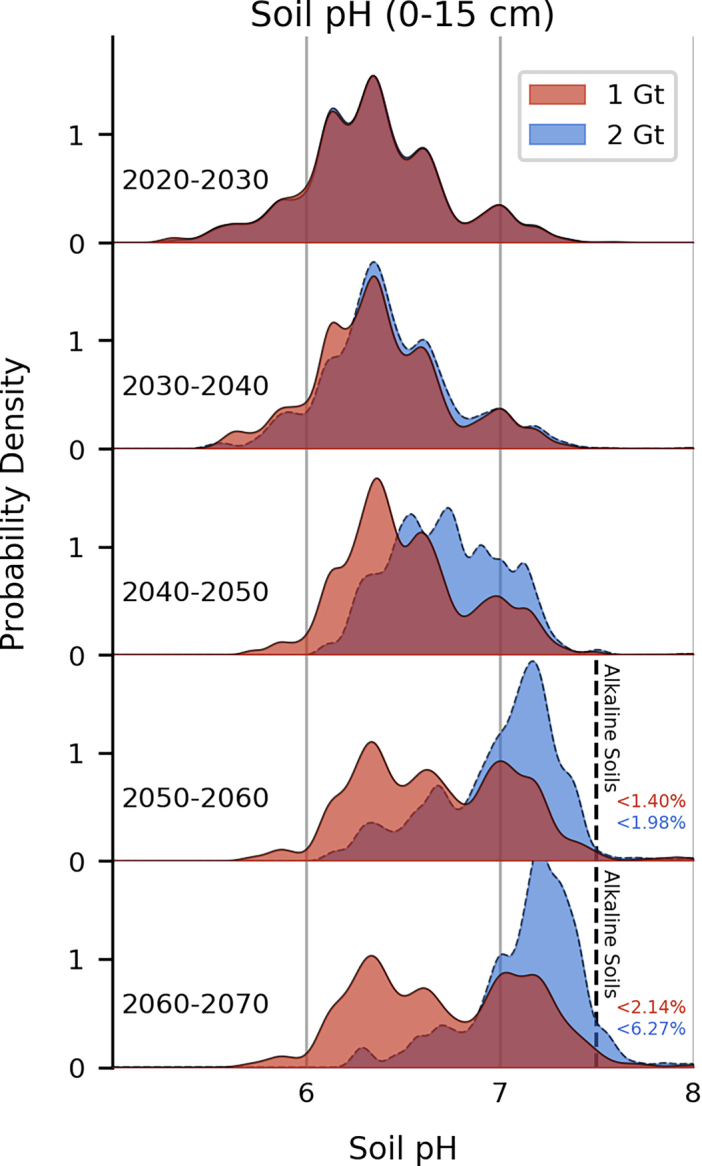
Probability density plots of U.S. cropland soil pH with EW deployment over successive decades (2020–2070). Histograms show topsoil pH results for 1 Gt rock yr^−1^ (red) and 2 Gt rock yr^−1^ (blue) extraction by 2070 scenarios for the Corn Belt states where EW is implemented. Decadal values for 2050–2060 and 2060–2070 are percentage of cropland area above a threshold pH value of 7.5 (denoted by the vertical lines).

Regulation of pH in US agricultural soils is important for maintaining and improving crop yields, soil fertility^23^ and nitrogen fixation rates in legumes (for example, soybean)^24^. In our simulations, we show that EW regulates soil acidity by progressively increasing median farmland soil pH from 6.4 in 2020 to pH 6.7 or 7.1 by 2070 with the 1 and 2 Gt yr^−1^ rock extraction scenarios by 2070, respectively (Fig. 3a). These results support substituting basalt for agricultural limestone to manage soil acidity while maximizing CDR. Geospatial analysis of our results for the Corn Belt states over successive decades indicates that average topsoil pH typically remains close to the optimal range of nutrient uptake by major row crops with EW implementation^10^ (Extended Data Fig. 7). After five decades of EW, soil pH in about 6% of locations increases above 7.5, indicating the need for continuous monitoring of soil pH, a current standard farm management practice. For these cases, EW practices could be halted to avoid micronutrient deficiency, particularly iron^25^, without major impact on overall US CDR (less than 5%). Over time, EW reduces the fraction of cropland soil areas classified as acidic (pH less 6.5) from about 0.7 to between 0.4 and 0.05 by 2070, when acidified soils are practically eliminated for the 2 Gt rock extraction scenario (Fig. 3b).

EW releases crop nutrients phosphorus (P) and potassium (K), as basaltic minerals undergo dissolution, with the potential to reduce the requirement for supplemental P and K amendment. These nutrients are usually obtained from expensive chemical fertilizers with large environmental footprints and geopolitically unstable global supply chains^26^. Calculated P release patterns by EW reflect fast-weathering apatite and across the top ten Corn Belt states (by CDR potential) release rates are typically 10–30 kg ha^−1^ yr^−1^ (Fig. 3c). As for rock phosphate-derived fertilizers, the orthophosphate form of mineral P from basalt can adsorb strongly to soil minerals, particularly at acidic pH values, but is released and becomes more bioavailable as soil pH increases^27^. For K release from slower-weathering feldspars, rates increase from 20 kg ha^−1^ yr^−1^ in 2030–2040 to 60–70 kg ha^−1^ yr^−1^ by 2070 (Fig. 3d). These rates are comparable to the range of maintenance P and K fertilizer application rates used in the Midwest for soybean, maize and wheat (Fig. 3c,d), although rates vary with crop and soil type. Rock supply states for farmland in the western USA produce basalt with a higher P and K content than on the east coast, and EW nutrient release rates that could exceed present average fertilizer rates. On the basis of present agronomic practices for three crops (soybean, wheat and maize) across ten states, our analysis shows that EW practices can partially replace expensive P (urea phosphate, US$890 t^−1^; diammonium phosphate, US$940 t^−1^) and K (potash US$840 t^−1^)^28^. Regardless of market price volatility, this analysis suggests that EW practices could avoid millions of tons of CO_2_ emissions linked to P and K fertilizer production and distribution^11^.

**Extended Data Fig. 8:**
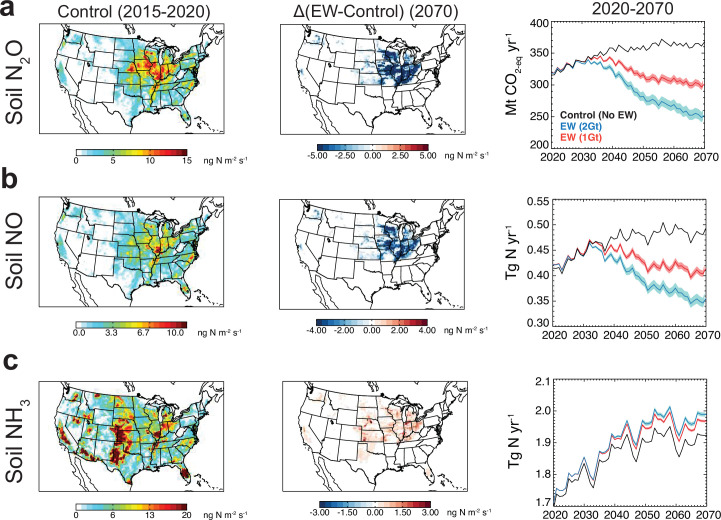
U.S. Soil agriculture nitrogen gas fluxes and responses to EW. Modelled emissions of (**a**) nitrous oxide (N_2_O) (**b**), nitric oxide (NO) and(**c**) ammonia (NH_3_). Left panels (Control) show the spatial distribution of present-day (averaged 2015–2020) fluxes. Middle panels (Δ EW-Control) show geospatial changes in fluxes due to EW in 2070. Right hand panels show time series annual fluxes from 2020 to 2070 for Control, 1 and 2 Gt yr^−1^ scenarios, shaded areas denote 90% confidence interval.

Mitigation of nitrous oxide (N_2_O) emissions from agricultural soils as the dominant anthropogenic source of N_2_O, is an integral part of US net-zero pathways^5^; N_2_O is a potent long-lived GHG that also causes stratospheric ozone depletion. Reductions in soil N_2_O emissions have been reported following basalt applied to farmland^29,30^ and maize production in controlled environments^31^ and represent important climate benefit of EW^32^. These reductions are probably linked to soil pH increases and analogous to the effects of liming on soil N_2_O fluxes. Expressed as CO_2_ equivalents (CO_2_e), simulated EW N_2_O reductions translate into 90 Mt CO_2_e yr^−1^ and about 120 MtCO_2_e yr^−1^ of avoided emissions by 2070 (1 and 2 Gt yr^−1^ rock extraction by 2070 scenarios, respectively; Extended Data Fig. 8) which improve EW GHG removal budgets by a further 36–45%, and potentially compensates for ocean-related CO_2_ degassing.

## Regional air quality improvements with EW

**Fig. 4:**
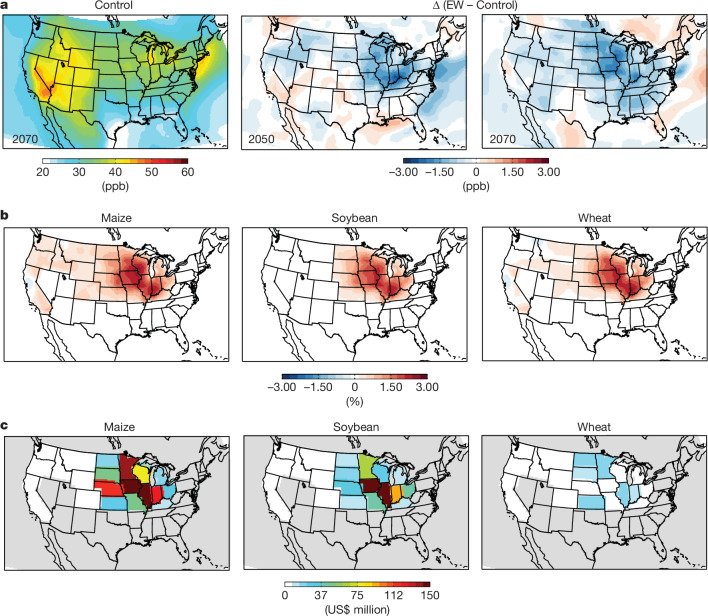
Benefits of enhanced weathering for surface ozone and crop production. **a**, Simulated summer surface ozone (O_3_) for 2070 (control; anthropogenic emissions + biomass burning + present-day biogenic emissions, no EW effects), with widespread reductions by 2050 and 2070 due to EW lowering soil nitrogen trace gas emissions. **b**, Average calculated increases in yields of maize, soybean and wheat for 2070 of three ozone exposure–crop yield functions. **c**, Calculated avoided economic yield losses for maize, soybean and wheat per state due to lower surface O_3_ exposure levels in 2070 with EW.

**Extended Data Fig. 9:**
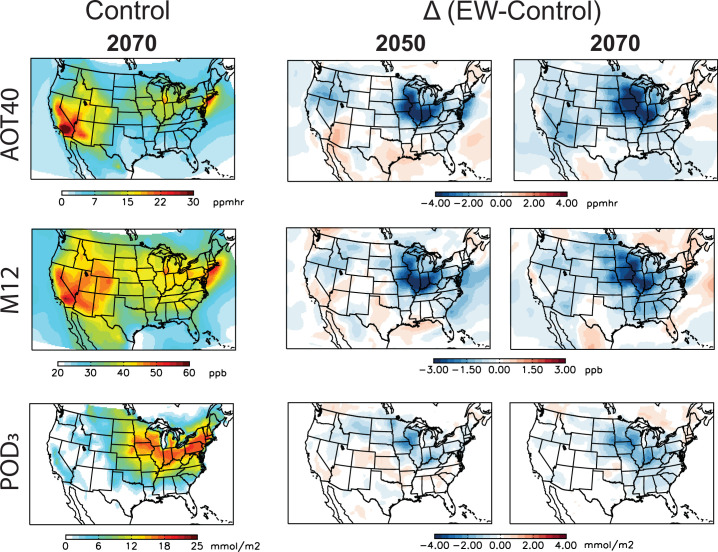
U.S. crop yield-ozone metrics and responses to EW. Simulated summer surface AOT40 (ppm-hr), M12 (ppb) and POD_3_ (mmol m^−2^) for 2070 (control, no EW) and changes in response to EW practices in 2050 and 2070 (2 Gt yr^−1^ scenario).

Our constrained EW scaling scenarios allow assessment of possible consequential effects on regional air quality (Extended Data Fig. 1 and Supplementary Table 4). We find that air quality improvements with EW are expected given that soil emissions of nitric oxide (NO) track decreases in N_2_O emissions (Extended Data Fig. 8) due to the rise in soil pH with EW increasing the ratio of N_2_ to N_2_O production during denitrification. Soil NO released to the atmosphere undergoes rapid oxidation to nitrogen dioxide (NO_2_) to generate tropospheric ozone (O_3_) (ref. ^33^), a strong oxidant detrimental to crop health^34^. Thus, lowering NO emissions with EW can decrease ground-level O_3_ production. We focus on the response of surface O_3_ reductions and use three O_3_ exposure metrics to quantify its impact on crop yields^35^ (Supplementary Table 5). Decadal-mean summer surface O_3_ concentrations are highest in highly populated and industrial regions, such as the eastern USA and California, with the largest anthropogenic emissions (Fig. 4a). With EW, however, we find widespread reductions in surface O_3_ throughout the Corn Belt in 2050, which expand further by 2070 (Fig. 4a). Using established functions, these surface O_3_ reductions translate into yield gains of up to 3% for maize, soybean and wheat throughout the Corn Belt when averaged across three O_3_-damage metrics (Fig. 4b and Extended Data Fig. 9). These gains are comparable to those obtained through mitigation strategies such as methane emission controls and ozone-resistant cultivar selection^35^. Mitigation of surface O_3_, an overlooked indirect benefit of EW, may help counteract O_3_ increases estimated by some Earth system models with future climate change under the SSP2-4.5 pathway^36^. However, if future anthropogenic emissions fall closer to a business-as-usual scenario the possible benefits of EW-related O_3_ reductions will be more uncertain^37^. At the state level, our results indicate marked regional damage-avoided costs from ozone-induced reductions in yields. For maize and soybean, state-specific avoided financial losses could be US$75–150 million annually by 2070, particularly in central growing states such as Iowa and Illinois, with avoided losses of up to US$10–30 million annually per state for wheat (Fig. 4c).

**Extended Data Fig. 10:**
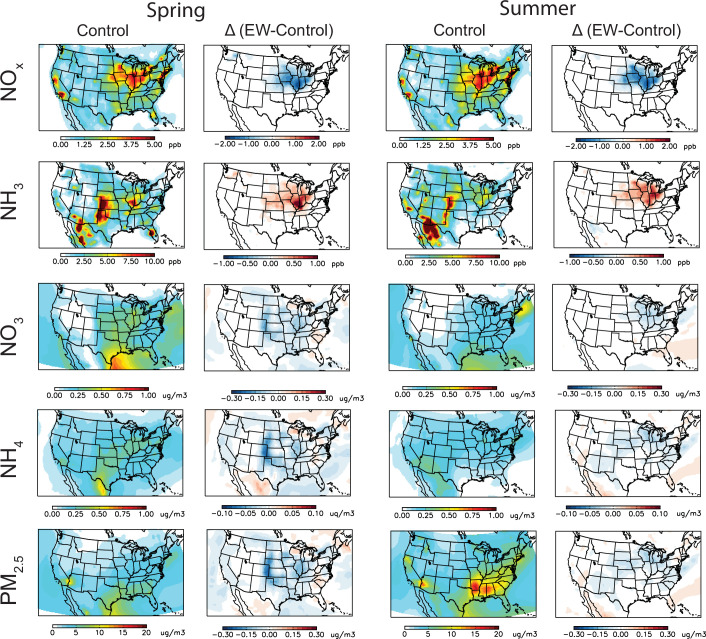
U.S. spring and summer air quality and PM_2.5_ responses to EW. Simulated PM_2.5_ and relevant PM_2.5_ chemical species for control in 2070 and changes due to EW, for nitrogen oxides (NO_x_ in ppb), ammonia (NH_3_ in ppb), nitrate (NO_3_ in μg m^−3^), ammonium (NH_4_ in μg m^−3^) and anthropogenic PM_2.5_ (in μg m^−3^) during spring and summer.

**Extended Data Fig. 11:**
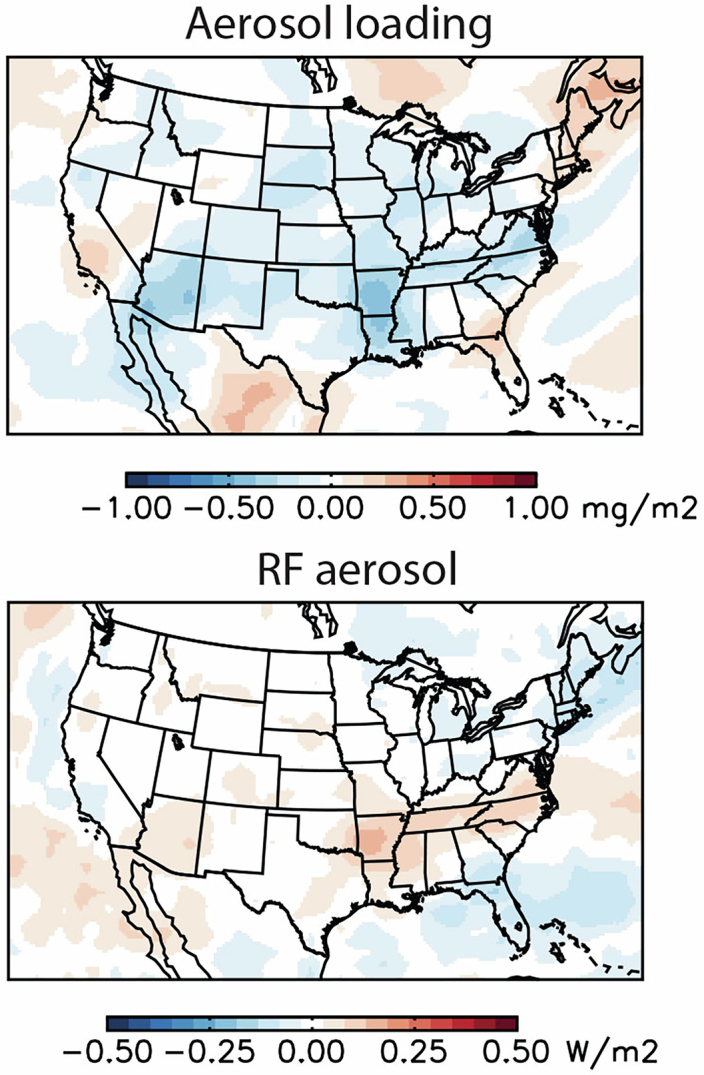
Annual changes in fine aerosol loading (mg m^−2^) and radiative forcing with EW. (**a**) Changes in fine aerosol loading with EW and (**b**) radiative forcing (RF) from aerosol scattering (W m^−2^) in response to EW practices (2 Gt yr^−1^ rock extraction by 2070 scenario).

Increases in soil pH with EW carry the risk of increasing aerosol pollutants harmful to human health by stimulating ammonia (NH_3_) volatilization at higher pH^38^. In particular, ammonia has an important role in the formation of fine particulate matter (particles less than 2.5 µm in diameter; PM_2.5_). It reacts with nitric acid, derived from the reaction of NO_2_ with water, to form secondary inorganic nitrate and ammonium aerosols, which catalyse production of PM_2.5_. In our modelling, EW increases soil NH_3_ emissions by 4–6% by 2070 (Extended Data Figs. 8 and 10), but with reduced soil NO emissions limiting production of nitric acid, the formation of secondary inorganic nitrate and ammonium aerosols is reduced. Consequently, we calculate reductions of 0.2 μg m^−3^ (about 8%) and 0.1 μg m^−3^ (about 3%) in spring and summertime PM_2.5_ by 2070, respectively. Counterintuitively, therefore, EW may be an effective measure to control future PM_2.5_ formation in agricultural regions and is consistent with reducing emissions of nitrogen oxides to control PM_2.5_ in California^39^. Small reductions in total aerosol loading with EW could, however, lead to localized annual positive radiative forcing from aerosol scattering of less than 0.1 W m^−2^ (Extended Data Fig. 11).

## Spatial and temporal costs of CDR

**Fig. 5:**
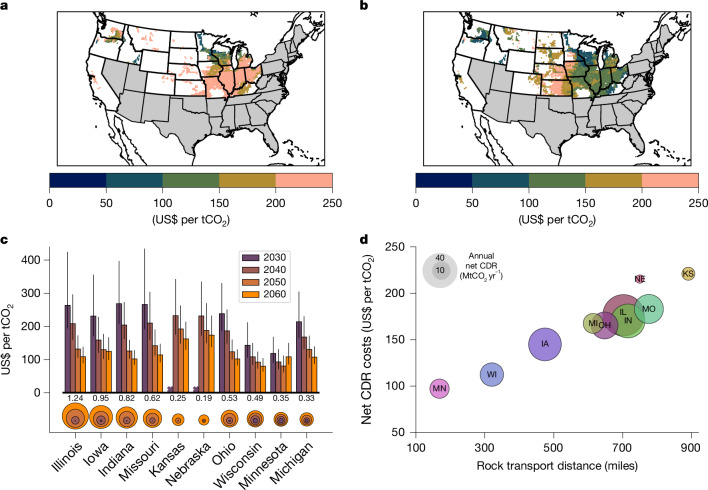
Costs of EW implementation in the USA. **a**,**b**, Geospatial pattern costs of CDR by EW for the 2 Gt rock extraction scenario in 2040–2050 (**a**) and 2060–2070 (**b**). **c**, Averaged state-level costs of net CDR by EW for successive decades between 2030 and 2060, with 90% confidence limits. The circles show corresponding cumulative net CDR by decade for each state and the labels denote values for 2060–2070 (GtCO_2_). **d**, Relationship between state-specific costs of net CDR and average distance between basalt supply state and farmland for 2060; the diameter of each circle indicates the annual net CDR in 2060. Standard abbreviations indicate US states, each state being represented by a different colour for clarity.

Geospatially mapped CDR costs show marked heterogeneity across the USA reflecting a combination of cropland distance from basalt source regions, timing of EW deployment and evolving CDR rates (Fig. 5a,b). By 2040–2050, spatial patterns show that CDR costs are lowest in crop states that start EW deployment early, such as Minnesota and Wisconsin (Fig. 5a). By 2060–2070, widespread reductions in CDR costs occur as a result of cumulative increases in CDR and lower energy costs for transportation and rock grinding, with most states achieving CDR at a cost of less than or equal to US$150 tCO_2_^−1^ (Fig. 5b). Central US agricultural states (for example, Kansas and Nebraska) are the exception in which costs remain stubbornly high mainly as a result of long transport distances of crushed rock from supply states; however, these states are relatively minor contributors to total US CDR (Fig. 1b). This geospatial cost analysis strengthens support for early implementation of EW in northern Corn Belt states.

Average CDR costs vary between states, being highest in the first decades of EW deployment before declining to a range of about US$100–130 tCO_2_^−1^ by 2050, including three states (Iowa, Illinois and Indiana) that contribute most to the US total (Fig. 5c). This range compares favourably with an initial global techno-economic assessment of EW for 2050 that included estimates for the USA (US$160–180 tCO_2_^−1^)^2^ and a techno-economic assessment for EW in the Midwestern USA (US$46–476 tCO_2_^−1^)^40^. Our analysis indicates that most agricultural states undertaking EW reach the suggested threshold (about US$100 tCO_2_^−1^) for making CDR technologies affordable and ready for large-scale deployment after a couple of decades^41^. Calculated costs of CDR with EW are competitive compared to other proposed CDR strategies, but these await comparable cost analyses that include state-by-state assessment and future projections.

Opportunities exist for marked cost reductions in CDR with EW. Long transport distances between basalt and crop states in the USA compared to, for example, the United Kingdom^11^ and Brazil^42^, are a key factor contributing to variations in CDR costs between states by 2050 (Fig. 5d). In line with emissions accounting for freight operations, costs assume that road transport vehicles will perform a different activity after basalt delivery^43^. Modifying our analysis to include costs for a return journey increases CDR costs by about 15% for the first decade of deployment and much less thereafter with the electrification of the transport network. Post-2050, the transition to electrified transportation systems weakens this constraint by lowering the C-emission penalty. Mass transportation of crushed rock from source states to crop states by high-capacity barge traffic on navigable inland river systems could help to lower these costs and also reduce operational CO_2_ emissions^40^, and warrants further analysis.

## Social licence and an equitable transition

Obtaining a social license to deploy EW equitably is critical. Research on US perceptions reveals a lack of awareness and knowledge of both CDR^44^ and EW^7,8^. When EW is described in detail to people, concerns include environmental risks (especially to wildlife and oceans), alongside a worry that CDR represents a temporary fix when the main need is to arrest rising GHG emissions^7^. Hence, parallel deep reductions in anthropogenic emissions is the sine qua non for gaining societal acceptability of any CDR deployment.

Outreach and dialogue with local stakeholders and communities are also required, linked to transparent and rigorous MRV (Table 1). Stakeholders include the general community, the farming, mineral extraction and supply chain industries and those with local political voice. Whether this transition will prove fair for US agricultural communities is a critical but little understood question, with equity issues (procedural, distributional and recognition justice)^45^ important for cultivation of social license. Experience from other technologies highlights early and continuing dialogue to map and respond to local values and concerns, equitable sharing of benefits with communities (which could also lead to increased EW deployment costs), transparent monitoring and control of impacts on local habitats and species, and attention to building trustworthy relationships^46^.

Environmental justice is a mandatory requirement of the clean energy transition through US Department of Energy Justice40 principles^47,48^. Hence, a key research need is the development of tools for evaluating equity impacts of EW deployment across the USA integrated with both MRV appraisals and mapping of local community and stakeholder concerns (Table 1).

## Upscaling challenges

Mobilizing an EW industry on the scale discussed here is a major undertaking and could take several decades, hence our scenarios require 50 years to fully deploy. At present, the US quarrying sector handles about 5.5 Gt yr^−1^ of processed ore and rock waste^49^, suggesting that the capacity to scale an EW industry in coming decades by 1 or 2 Gt of rock annually would be challenging but achievable. Additionally, key supply states in the Midwest have under-used capacity for excavation and crushing because of declines in iron ore mining and processing that could be repurposed for EW with basalt. Demand for up to 2 Gt of basalt to meet EW requirements in future could require employment of 60,000–80,000 more people (assuming 30–40 workers per Mt yr^−1^ production)^50^. However, any labour gains need to be balanced against potential reductions in employment if demand for P and K fertilizers and limestone were to fall with EW deployment. Whether EW scales to such an extent is uncertain given that US pathways to net-zero suggest a dominant role for BECCS and DACCS augmented by subsidiary contributions from other CDR strategies^5,6^.

Upscaling of EW must also evaluate possible environmental impacts and human health risks before and during deployment^51^, as emphasized by detailed life-cycle assessments of the processes embedded in the EW supply chain^52^. Mineral extraction and processing are long-standing activities with regulated health and safety guidelines for mitigation of the harmful effects of dust^53^. Scaling can only be accomplished safely if these procedures are followed by the quarrying industry and operators deploying EW on farmland to minimize human exposure and health risks. Risk of dispersal and transport of dust from EW practices necessitates adopting precautionary principles, as with liming operations.

Environmental risk assessments are required before EW deployment at any spatial scale together with due diligence checks on basalt feedstocks ahead of implementation to avoid potential trace metal hazards with respect to state-specific threshold criteria^51,54^. Rigorous monitoring of the plant–soil–water environment system is required pre- and post-EW treatment with basalt to track movement of trace metals and assess possible negative consequences for the biosphere^51^. In freshwater systems, extensive EW deployment may offset the widespread acidification of US inland waters following industrialization^55^. Nevertheless, direct alkalinity changes through EW could lead to uncertain impacts on downstream ecosystems and requires monitoring.

Lastly, our carbon cycle simulations adopt a baseline application rate of crushed basalt (40 t ha^−1^) with the overarching goal of determining feasibility of EW in the USA at scale, costs and other possible outcomes for soil and atmospheric chemistry. However, considerable scope exists for optimizing local application rates on individual farms and the use of a wider range of alkaline soil amendments^2^. Important for accelerating the pace of EW implementation will be transparent MRV schemes that are accurate and cost-effective, not only to increase the confidence of markets, investors and federal agencies in EW^15^ (Table 1) but also to avoid perceptions of ‘greenwashing’.

## Conclusions

Our analysis suggests that transitioning to manage US agriculture with EW and crushed basalt is a promising practical innovation for improving farming practices to extract carbon from the air and reverse agriculture’s contribution to climate change, although knowledge gaps remain (Table 1). With straightforward technological pathways to upscaling that use existing supply chains, EW offers a means of sequestering atmospheric carbon to assist with US net-zero objectives, while also improving air quality critical to crop and human health and soil fertility. Nevertheless, despite the large potential deployment area, EW is now absent from US government net-zero policy discussions^5,6^. Federal recognition of EW potential will be important because fiscal policy initiatives are needed to spur deployment, especially in the early stages of implementation when costs are highest.

Our results clarify the substantial challenges in mobilizing an EW industry in the USA at scale. Responsible upscaling of EW in future decades requires careful consideration of potential negative externalities, including monitoring of the environmental risks of basalt application on plant–soil–water environment systems^51,52^, alongside robust public engagement. Advancing the science to build confidence in EW also requires addressing the challenges of improving our understanding of weathering rates across a wide variety of soils and environments, rates of hydrological export of weathered products and the development of a robust framework to empirically track carbon removal (Table 1). Finally, we emphasize that efforts to remove atmospheric carbon by means of any CDR technology will ultimately be futile for addressing the climate crisis without urgent and drastic emissions reductions and a transition to clean energy^9^.

## Methods

Detailed methods are provided in Supplementary Information.

## Online content

Any methods, additional references, Nature Portfolio reporting summaries, source data, extended data, supplementary information, acknowledgements, peer review information; details of author contributions and competing interests; and statements of data and code availability are available at 10.1038/s41586-024-08429-2.

## Supplementary information

Supplementary Information Supplementary Methods, Tables 1–5, Figs. 1–14 and References.

Supplementary Data 1 geochemistrydata_normcode.

Supplementary Data 2 GEOROC_USA_Plutonics.

Supplementary Data 3 GEOROC_USA_Volcanics.

Supplementary Data 4 State-specific maximum rock extraction 2020–2070; both scenarios (t yr^−1^).

Supplementary Data 5 US mineralogy proportions by state.

Supplementary Data 6 Normative mineralogy stoichiometry and kinetic parameters.

## Data Availability

The model datasets used in this study are: air quality crop damage, available on Zenodo at 10.5281/zenodo.14755340 (ref. ^56^); soil nitrogen fluxes, available on Zenodo at 10.5281/zenodo.14755401 (ref. ^57^); EW US datasets–outputs source data, available on Zenodo at 10.5281/zenodo.14755423 (ref. ^58^); and US river catchment chemistry input–output data files, available on Zenodo at 10.5281/zenodo.14605782 (ref. ^59^). MATLAB EW datasets are available on Zenodo at 10.5281/zenodo.10940280 (ref. ^60^). River catchment datasets are available on Zenodo at 10.5281/zenodo.14605782 (ref. ^59^). One-dimensional EW soil profile data files and plotting files for the Energy Farm model versus observation evaluation are available on Zenodo at 10.5281/zenodo.12806314 (ref. ^61^).

## Code availability

MATLAB EW code is available on Zenodo at 10.5281/zenodo.10940280 (ref. ^60^). The river catchment chemistry model is available on Zenodo at 10.5281/zenodo.14605782 (ref. ^59^). The NCAR Community Earth System Model (CESM2) (with CAM-chem) code is available on GitHub at https://github.com/ESCOMP/CESM. CLM5.0 is available through the Community Terrestrial System Model (CTSM) on GitHub at https://github.com/ESCOMP/ctsm. The CLM5 updated version of the nitrogen cycling scheme used in the paper is available on Zenodo at 10.5281/zenodo.8111541 (ref. ^62^). One-dimensional EW soil profile model code for the Energy Farm model versus observation evaluation is available on Zenodo at 10.5281/zenodo.12806314 (ref. ^61^).
