# Supplementary material for: Transforming US agriculture for carbon removal with enhanced weathering
Source: Nature. 2025 Feb 5;638(8050):425–34. doi: 10.1038/s41586-024-08429-2 (PMC11821523; doi:10.1038/s41586-024-08429-2)
Supplement: Supplementary file 1 — Supplementary Methods, Tables 1–5, Figs. 1–14 and References. [file 41586_2024_8429_MOESM1_ESM.pdf]

nature portfolio

https://doi.org/10.1038/s41586-024-08429-2

Supplementary information

Transforming US agriculture for carbon removal with enhanced weathering

In the format provided by the authors and unedited

Supplementary Information

Transforming US agriculture for carbon removal with enhanced weathering

David J. Beerling[1]*, Euripides P. Kantzas[1], Mark R. Lomas[1], Lyla L. Taylor[1], Shuang Zhang[2], Yoshiki Kanzaki[3], Rafael M. Eufrasio[4], Phil Renforth[5], Jean-Francois Mecure[6,7], Hector Pollitt[7,8], Philip B. Holden[9], Neil R. Edwards[7,9], Lenny Koh[4], Dimitar Z. Epihov[1], Adam Wolf[10], James E. Hansen[11], Steven A. Banwart[12], Nick F. Pidgeon[13], Christopher T. Reinhard[3], Noah J. Planavsky[14] & Maria Val Martin[1]

[1]Leverhulme Centre for Climate Change Mitigation, School of Biosciences, University of Sheffield, Sheffield, UK

[2]Department of Oceanography, Texas A&M University, College Station, TX

[3]School of Earth and Atmospheric Sciences, Georgia Institute of Technology, Atlanta, USA

[4]Advanced Resource Efficiency Centre, Management School, University of Sheffield, Sheffield, UK

[5]School of Engineering and Physical Sciences, Heriot-Watt University, Edinburgh Campus, Edinburgh, UK

[6]Exeter Business School, University of Exeter, Exeter, UK

[7]Cambridge Centre for Energy, Environment and Natural Resource Governance, The David Attenborough Building, Pembroke Street, University of Cambridge, Cambridge, UK

[8]World Bank, 1818 H Street, NW Washington DC 20433, USA

[9]Environment, Earth and Ecosystems, The Open University, Milton Keynes, UK

[10]Department of Ecology and Evolutionary Biology, Princeton University, Princeton, USA

[11]Earth Institute, Columbia University, New York, NY, USA

[12]School of Earth and Environment, University of Leeds, Leeds, UK

[13]Understanding Risk Research Group, School of Psychology, Cardiff University, Cardiff, UK

[14]Department of Earth and Planetary Sciences, Yale University, New Haven, USA

*Corresponding author. Email: d.j.beerling@sheffield.ac.uk

Supplementary Information includes:

Supplementary Methods
Supplementary Tables S1 to S5
Supplementary Figs. S1 to S14
Supplementary Data Files 1-6
References

Supplementary Table listing

Table S1. Costs for mineral extraction and crushing to aggregate (mm) sized particles for a 1.3 Mt/yr operation.
Table S2. Additional cost items associated with rock grinding to 100 µm for a 1.3 Mt/yr operation.
Table S3. Summary of the levelized costs for mineral extraction and processing to 100 µm in North America
Table S4. Summary of future anthropogenic and soil agriculture emissions in the U.S.
Table S5. Summary of ozone exposure metrics and relative yields for maize, soybean and wheat.

Supplementary Figure listing

Fig. S1. Basalt tock metal oxides in western and eastern regions.
Fig. S2. Geospatially mapped basalt phosphorus (P) and potassium (K) contents
Fig. S3. Variations in the chemical affinity term, C_{eq}, with soil temperature and infiltration rate
Fig. S4. Comparison of EW model basalt mineral weathering rates 1
Fig. S5. Comparison of EW model basalt grain weathering rates 2
Fig. S6. Comparison of EW model basalt grain weathering rates 3
Fig. S7. Projected future changes in energy prices by state
Fig. S8. Transport electrification paths, costs and emissions
Fig. S9. U.S. power generation regions and output
Fig. S10. Projected U.S. distribution of energy production by region
Fig. S11. Riverine HCO_3^- fluxes for six watersheds and both rock extraction scenarios
Fig. S12. Riverine alkalinity fluxes for six watersheds and both rock extraction scenarios
Fig. S13. Evolution of river carbonate saturation state for both EW scenarios
Fig. S14. Location of river basin flux sites for GENIE simulations

Supplementary Data File listing

Data File S1. USA_geochemistrydata_normcode_17-Feb-2023.tar.gz (1MB)
Data File S2. GEOROC_USA_Plutonics.tar.gz (171 KB)
Data File S3. GEOROC_USA_Volcanics.tar.gz (1.3 MB)
Data File S4. State-specific maximum rock extraction 2020-2070; both scenarios (t/yr).
Data File S5. US mineralogy proportions by state
Data File S6. Normative mineralogy stoichiometry and kinetic parameters

Detailed Methods

U.S. state-level basalt geochemistry and mineralogy datasets

We determined normative mineralogies[1] of U.S. basalts using geochemical data from the literature and online repositories[2-4] for 6430 mafic rock samples with complete major oxide data, SiO_2<57%, chemical index of alteration under 40% and RCO2[5,6]>0.25 (**Extended Data figure 2; figs. S1, S2**). Geochemical data are far more widely available than modal (e.g., X-ray diffraction determined) mineralogies, allowing assessment of variability between U.S. states. These idealised mineralogies are suitable for our reactive transport modelling but do not account for secondary minerals, altered/metamorphic minerals, xenoliths, trace carbonates or glass, see **Data Files S1–S3** for details and code. We assigned plagioclase based on normative anorthite and albite. In ~26% of samples, putative trace calcite led to calcic plagioclase. These samples are not plutonic and lack CO_2 data so cannot be corrected. Such samples were retained during the clustering process described below (**Extended Data figures 2,3**) to facilitate generation of a distinct pair of mineralogies for each state (**Data Files S1–S3**).

State-level rock production

For each state, we created potential rock production curves *P(y)* for year *y* by tuning the parameters of the logistic function:

$$P(y|m,r,L) = \frac{L}{1 + e^{-r(y-m)}} \qquad \text{Eq. 1}$$

The rationale for logistic functions is a slow roll-out phase of 10–20 years for rock production (with delays due to mining licenses, public acceptance, etc.), followed by a period of rapid expansion with the opening of new mines, before finally reaching steady-state production rates (**Extended Data figure 3; Data File S4**). It is also consistent with the growth of crushed stone production in the U.S. since the 1970s[7] which, despite the lack of a clear economic driver such as development of a durable carbon marketplace or federal subsidies, increased from 0.9 Gt in 1970 to 1.5 Gt in 2022. Parameter *m* (yrs) defines the logistic function inflection point (midpoint); the year when the state's rate of production maximizes. Our working hypothesis for this initial assessment is that states closer to croplands will maximize production sooner while states further away will lag and take over after the initial states encounter diminishing returns. Using base value m_0 = 2041, scaling constant m_1 = 10, each state has a normalized (0–1) distance to croplands d. For state, s, $m_s = m_0 + m_1 \times d(s)$. States close to croplands will maximize rock production around year m_0 while states further away at $m_0 + m_1$. A similar approach was followed for the rate of rock production r (Gt/yr): as $r_s = r_0 + r_1 \times h(s)$, with r_0 = 0.22 and r_1 = 0.07. These parameters allow the rock production rate to increase annually but remain sustainable without exceeding historical data.

Here *h(s)* is the normalized (0–1) historic production (2010–2018) of crushed traprock for each state[8]. The hypothesis is that states currently producing large quantities of rock can to ramp up production faster and achieve higher annual rates of rock production. Finally, the maximum rock production L_s achieved by each state at year 2070 increases with historical production but decreases with distance to croplands according to $L_s = 0.7 \times h(s) - 1.7 \times d(s)$, where 0.7 and -

1.7 are weighting factors on the distance and historical production allowing normalization of basalt production by state. A logistic curve $P(s|y)$ is thus obtained for each state, s, at year y. We then normalize so that $\sum_s P(s|\ 2070)$ = 1 or 2 Gt/yr by 2070 (**Data File S4**).

Allocation of rock supply states to demand states

The source states S providing basalt are represented as single pixels (n = 13) of a raster image at the location of the geographic centre of the state, while the croplands T are all the pixels ($n \approx 5000$) found within the boundaries of the target states where maize, soy or wheat are present in the CLM land cover representation. Optimised basalt distribution, maximizing net CDR, depends on all possible combinations between source states and targets:

$(S_1, T_1), (S_1, T_2), \ldots, (S_1, T_t), (S_2, T_1), (S_2, T_2), \ldots, (S_2, T_t), \ldots, (S_s, T_1), (S_s, T_2), (S_s, T_t), \quad \forall s \in S, \forall t \in T.$

The tuples are 1 for states supplying basalt to the target, and 0 if not. We used the PuLP v2.6 Linear Programming API to create the thousands of linear constraints required and solve the optimization.

Constraints on rock allocation to croplands

Each target field t in cropland state T can only receive basalt annually from a single state s ($\forall t \in T$): $\sum_i^s (S_i, T_t) \leq 1$. For each year y, production (t rock) $P_{s,y}$ constrains supply from every state s ($\forall s \in S$) : $\sum_i^t (S_s, T_i) * area(T_i) * appRate \leq P_{s,y}$ for target *area* in ha and *appRate* 40 t/ha.

Given the above constraints, we maximized total net CDR (in t CO_2) on each year y.

$$CDR_{Net}(y) = \sum_{s=1}^{S}\sum_{t=1}^{T}[CDR_{Gross}(S_s, T_t, y) - Tra_E(S_s, T_t, y) - Gri_E(S_s, y) - Spr_E(T_t, y) + Fer_E(T_t, y)] \times (S_s, T_t) \qquad \text{Eq. 2}$$

Here, Tra_E are the transport CO_2 emissions which depend both on source state and target on account of distance travelled, Gri_E the grinding and mining emissions which depended only on the source state assuming grinding takes place at the mining location, Spr_E the spreading emissions depended only on the target where the spreading will take place, and Fer_E avoided fertilizer emissions which depended on the target where the weathering occurs and P/K are released. All the processes are in tonnes of CO_2 as functions of year y. Life cycle CO_2 emissions for P- and K-fertilizer production were calculated as average values for different time horizons using the methodologies included in the Ecoinvent database[9] (summarized in Table S7, ref[10]) with projections of rising P fertilizer prices (2020-2070) for a medium resource scenario and maintaining K prices at current value, as described previously[10]. Fertilizer emissions have the opposite sign to the other secondary processes as the mineral release from EW results in substitution of fertilizer application and thus savings in both emissions and costs.

Costs provide a final constraint. For the optimization we defined total EW costs for year y (in $) as

$$CDR_{Cost}(y) = \sum_{s=1}^{S}\sum_{t=1}^{T}[Tra_C(S_s,T_t,y) + Gri_C(S_s,y) + Spr_C(T_t,y) - Fer_E(y)] \times (S_s,T_t) \quad \text{Eq. 3}$$

For every state, s ($\forall s \in S$) and every target t ($\forall t \in T$) we restricted costs below certain price points (C_{thr} = \$300/tCO_2) as

$$\left[{CDR_{Cost}(s,t,y)}\Big/{CDR_{Net}(s,t,y)}\right] \times (s,t) \leq C_{thr} \quad \text{Eq. 4}$$

All nominal costs and secondary emissions are time dependant on simulation year as they require E3-US annual model projections as inputs. Projected diesel prices are used to calculate annually updated transport and spreading costs while electricity prices are used to calculate grinding costs as well as transport and spreading once the transport network and agricultural machinery have been electrified, later in the model run (see respective sections, below). For secondary emissions and when diesel is the primary fuel, appropriate fuel efficiency metrics are used (see respective sections, below); as processes are gradually electrified during the transient run, projected life cycle emissions of the electricity network are used to calculate emissions for processes.

Climate-carbon-nitrogen cycle simulations

Our model framework starts with U.S. climate simulations (2020–2070) from the medium-mitigation future pathway climate (SSP2-4.5) ensemble of CMIP6 runs with the Community Earth System Model v.2 (CESM2)[11]. These drive the Community Land Model v.5 (CLM5), the CESM2 land model, to simulate at high spatial (23 km × 31 km) and temporal (30 min) resolution terrestrial carbon and nitrogen cycling with prognostic crop growth[12] and other ecosystem processes, including heterotrophic respiration. CLM5 simulates monthly crop productivity, soil hydrology (precipitation minus evapotranspiration), soil respiration and nitrogen cycling. CLM5 has representation of eight active crop functional types, each with specific ecophysiological, phenological and biogeochemical parameters. CLM5 includes CO_2 fertilization effects on agricultural systems benchmarked against experiments and observations[12]. We initialized CLM5 simulations in 2015 using fully spun-up conditions from global runs at ~100 km × 100 km resolution, adding an extra 200-year spin-up in the regional set-up to stabilize the CN pools to the higher resolution setting. Atmospheric CO_2 increases ~100 ppm from 2015 to 2070, as defined by SSP2-4.5. In our CLM5 simulations with rising CO_2 and climate change drive increases in crop NPP and reductions in evapotranspiration, which can facilitate weathering in our soil profile EW model[10].

CLM5 includes an interactive nitrogen fertilization scheme that simulates fertilization by adding nitrogen directly to the soil mineral nitrogen pool to meet crop nitrogen demands using both synthetic fertilizer and manure[12]. The model represents inorganic N transformations based on the DayCent model, which includes separate dissolved NH_4^+ and NO_3^- pools, as well as environmentally controlled nitrification, denitrification and volatilization rate. Land use and land cover remains constant to spatially track basalt application through the years. Synthetic fertilizer

application is prescribed by crop type[13], with rates increasing by 3% per decade from 2020 to 2050 in agreement with current N-fertilizer usage increases[14,15], and then stabilizing from 2050 to 2070. Average U.S. CLM5 fertilizer application rates in 2020 (102 kg N ha^{-1} yr^{-1}) are consistent with current practices[14].

We model the effect of basalt addition on fluxes of N_2O, NO and NH_3 from soil, with the updated denitrification DayCent module, including soil hydrology (e.g., water-filled pore space and volumetric soil water content)[16], with additional implementations[17]. Our parameterization considers the possible effect of increased soil pH from basalt application on direct agriculture emissions of N_2O, NO and NH_3 volatilization. Indirect soil nitrogen gas emissions are not explicitly modelled, but the small bias is likely marginal, given that these emissions account for less than 5% of total agricultural N_2O emissions[17]. Direct cropland CLM5 soil nitrogen trace gas emissions are within the range of estimates for U.S. based field observations, bottom-up inventories and other land surface models (ref[17], see Fig 4 and Table 3 therein). Although land surface modelling of nitrogen cycling process is uncertain[17], our framework gives relative changes in N_2O emission with EW consistent with reductions observed in basalt-treated plots in EW field trials in the U.S. Corn Belt[16,17] and increases in soil NH_3 fluxes reported from EW greenhouse trials[18]. Our CLM5 nitrogen cycling parameterizations for EW effects are the best available given current datasets[17] and could be developed further as more observational data and constraints on soil nitrogen fluxes from EW field trials become available.

1-D Soil Profile EW Modelling

Our analysis uses a 1-D vertical reactive transport model for rock weathering with dynamic monthly soil infiltration from CLM5 and water transport through a series of soil layers[19]. The transport equations include source terms representing rock grain dissolution and alkalinity within the soil profile, accounting for crop productivity and advancements incorporating the effects of the biogeochemical transformations of nitrogen fertilizers following the methodology of ref[10]. The core model accounts for changing dissolution rates with soil depth and time as grains dissolve, and chemical inhibition of dissolution as pore fluids approach equilibrium with respect to the reacting basaltic mineral phases, and the formation and dissolution of pedogenic calcium carbonate mineral in equilibrium with pore fluids[19]. Simulations for each state incorporate basalts with representative state specific mineralogies, as determined by cluster analysis of our normative mineralogy datasets (**Extended Data figure 3; Data File S5**) and specified mineral stoichiometries and kinetic rate parameters (**Data File S6**).

We assume the applied crushed basalt is tilled into the surface of soil (0 – 15 cm depth), and model the weathering of this material with a log-normal particle size distribution (PSD)[5] using unique surface-area-mass relationship with the theory developed previously[19]. Crushed basalt is assumed to be distributed evenly in the tillage layer. As the existing PSDs at each soil layer are at different stages of weathering, the combined PSD at each level, and for each mineral, is calculated and tracked over time. We account for repeated basalt applications by combining the existing PSD with the PSD of the new application. Simulated mineral dissolution fluxes from the model output were used to calculate the release of P and K over time. Mass transfer of P within the relatively more rapidly dissolving[20] accessory mineral apatite is calculated based on the P content of the rock and the volume of bulk minerals dissolved during each time step.

The mathematical model combines a multi-species geochemical transport model with a mineral mass balance and rate equations for the chemical dissolution of basaltic mineral phases. The model includes an alkalinity mass balance that includes the effect of crop nutrient uptake, fertilizer applications and soil N cycling, soil respiration and dynamic calculation of pH in soil pore waters. The main governing equations are described below, further details are provided in ref[19].

Transport equation. The calculated state variable in the transport equation is the dissolved molar equivalents of elements released by stoichiometric dissolution of mineral i, in units of mole L^{-1}. ϕ is volumetric water content, C_i is dissolved concentration (mole L^{-1}) of mineral i transferred to solution, t is time (mths), q is vertical water flux (m y^{-1}), z is distance along vertical flow path (m), R_i is the weathering rate of basalt mineral i (mole per litre of bulk soil mth^{-1}) and C_{eq_i} is the solution concentration of weathering product at equilibrium with the mineral phase i (Eq. 5).

$$\phi \frac{\partial C_i}{\partial t} = -q \frac{\partial C_i}{\partial z} + R(pH, tmp)\left(1 - \frac{C_i}{C_{eq}(tmp, q)}\right) \qquad \text{Eq. 5}$$

Values for C_{eq} for each of the mineral phases in the state-specific basalt grains are computed by calibrating the results of the soil 1-D model against those of a PHREEQC 1-D reactive transport model (RTM). The RTM incorporated the same kinetic reaction equations as this study's 1-D model and full geochemical speciation equilibria for solutes and minerals including equilibria that describes cation exchange on soil exchange surfaces and the formation of secondary minerals[19]. It includes the secondary mineral phases consistent with basalt weathering[21]: calcite, amorphous silica, gibbsite and goethite, that create sinks for dissolved calcium, silicon, aluminium and iron, respectively.

Calibration was undertaken against the time evolution of mass transfer for each mineral with minimisation of residual errors between the two models over 1 year. These values of C_{eq} are computed for a range of soil temperatures and infiltration (flow) rates: specifically, temperature (10, 15, 20, 25°C) and flowrate (0.25, 0.5, 1, 1.5, 2, 2.5, 3 m/yr) for a profile consisting of particles of radius 10µm (**fig. S3**). This particle size provides a benchmark for the maximum dissolution rate, which is lower than that for the larger particle sizes considered in the simulations. Therefore, this representation of weathering kinetics provides a conservative lower bound on dissolution rate due to its inhibition upon approaching solubility equilibrium, represented by C_{eq}.

Calibrating the 1-D soil profile model of this study, with temperature and flow-dependent C_{eqs} allows it to accurately reproduce mineral and basalt weathering rates computed by the detailed PHREEQC RTM (**figs. S4–S6**). Thus, soil profile EW rates for any given pixel are constrained by temperature- and infiltration rate-dependent chemical inhibition term which slows the dissolution rate as the chemical state of the fluid approached equilibrium with the respective mineral phases (Eq. 5). **Figs. S4–-S6** demonstrate that the simplified mass transfer inhibition term in Eq. 5 successfully described the same kinetic behaviour of mineral and basalt weathering calculated by the RTM. Note cation exchange on soil exchange surfaces and the formation of secondary minerals is not explicitly dealt with but handled implicitly via C_{eq} calibration with the PHREEQC model that includes these processes.

Rates of basalt grain weathering define the source term for weathering products and are calculated, for each component mineral and its relative reactive area in the basalt grains, as a function of soil pH, soil temperature, soil hydrology, soil respiration and crop net primary productivity (NPP)[19]. We adopt a methodology that treats basalt reactive surface as a fractal[22]. The fractal dimension provides a means of consolidating measurements taken at different scales. At the particle scale, we model the surface area geometrically and relate it to the radius of individual particles[19]. The vertical water flux is zero when pore water content is below a critical threshold for vertical flow. Weathering occurs under no-flow conditions and the accumulated solutes in pore water are then advected when water flow is initiated under sufficient wetting, tracked using a single bucket model.

Mineral mass balance. The change in mass of basalt mineral i, B_i, is defined by the rate of stoichiometric mass transfer of mineral i elements to solution. Eq. 6 is required because we are considering a finite mass of weathering rock, which over time can react to completion, either when solubility equilibrium between minerals and pore water composition is reached, or when applied basalt is fully depleted.

$$\frac{\partial B_i}{\partial t} = -R(pH, tmp)\left(1 - \frac{C_i}{C_{eq}(tmp, q)}\right) \qquad \text{Eq. 6}$$

An additional transport equation for Ca ion (mol L^{-1}) is included for secondary mineral formation of the calcium carbonate phase calcite

$$\frac{\partial Ca}{\partial t} = -q\,\frac{\partial Ca_{sol}}{\partial z} + \sum_{i=1}^{nmin} R(B_i)\, B_{ca_i} \qquad \text{Eq. 7}$$

Where *nmin* is the number of minerals, $Ca_{sol} = \min(Ca, Ca_{sat})$ and C_{sat} is the concentration of Ca ion in pore fluids calculated to be in equilibrium with the carbonate mineral phase. We also define $Ca_{precip} = Ca - Ca_{sol}$. B_{ca_i} is the mass fraction of calcium within each mineral, Ca_{sol} is the concentration of calcium in solution and Ca_{precip} the calcium precipitate with units of concentration; all variables have units of mole/L.

These equations form a coupled system of partial differential equations for all mineral components of the basalt and calcite[19]. We solve these numerically with a Crank-Nicholson finite different scheme with discretized depth and time. Two additional equations are required to solve the system: a Dirichlet boundary condition at the soil surface $C = 0$ at $z = 0$, and a Neumann boundary condition at the bottom of the soil profile where the concentration gradient is set to zero. An initial condition is specified as a concentration field of zero mole L^{-1} at $t = 0$, and a specified initial basalt mass uniformly distributed vertically over the top 15 cm of soil.

Removal of weathering products. The total mass balance over time (Eq. 8) for basalt mineral weathering allows calculation of the products transported from the soil profile. The total mass of weathering basalt is defined as follows where m is the total number of weathering minerals in the rock, t_f is the duration of weathering and L is the total depth of the soil profile. We define q as the

net monthly sum of water gained through precipitation and irrigation, minus evapotranspiration (i.e., infiltration), as calculated by CLM5. Infiltration is capped below by zero within the model, and the effect of negative q, which is to dry the soil, is accounted for with a simple single bucket model. When q is negative, the bucket is drained down to wilting point, and when positive the bucket is filled up to field capacity at a specific pixel. When the bucket is below field capacity and the q is positive, q is scaled down by the fraction of the bucket which is empty. Total weathered basalt is given by (Eq. 8)

$$\text{Total weathered Basalt} = \sum_{i=1}^{m} [\phi \int_{z=0}^{L} C_i(t,z)\, \mathrm{d}z + q \int_{t=0}^{t_f} C_i(t,L)\, \mathrm{d}t] \qquad \text{Eq. 8}$$

Modelling Soil Nitrogen Fertilizer Effects on EW. Simulation of the effect of nitrogen cycling processes on EW is via sixteen stoichiometric nitrogen transformations included with the CLM5 code that influence the soil weathering environment[10]. The modelling accounts for 20 layers in the soil profile at each location with a monthly time-step; variables passed from CLM5 by time and depth to the 1-D soil model are given in ref[10] At each depth, we compute nitrogen transformation effects on soil water alkalinity with reaction stoichiometries that add or remove alkalinity. Together with soil CO_2 levels, this affects pore water pH and the aqueous speciation that determines mineral weathering rates. This allows us to account mechanistically for the impact of soil acidification, including that from N fertilization on EW and CDR, a potential source of nitric acid weathering at low pH that limits CDR by EW in cropland. Dynamic modelling at monthly time-steps resolves seasonal cycles of CDR via alkalinity fluxes and soil carbonate formation/dissolution in response to future changes in atmospheric CO_2, climate, land surface hydrology, and crop and soil processes. The effect of the nitrogen cycle on the soil acidity balance is derived from nitrogen transformations associated with the production or consumption of hydrogen ions.

Following ref[10], we assigned a stoichiometric acidity flux $\Delta H_{i,\mathrm{N}}$ (mol H^+ mol^{-1} N) to each nitrogen flux $F_{i,\mathrm{N}}$ (gN m^{-3}soil s^{-1}) calculated by the CLM5 code. The product ($F_{i,\mathrm{N}} \cdot \Delta H_{i,\mathrm{N}}$), with appropriate unit conversions, gives the acidity flux during the time-step Δt (month) for the ith reaction of the CLM5 nitrogen cycle. Their sum (Eq. 9) is, therefore, the total change in acidity $\Delta \mathrm{Acidity}_{\mathrm{N}}$ due to the CLM5 nitrogen cycle:

$$\Delta \mathrm{Acidity}_{\mathrm{N}} = \sum(F_{i,\mathrm{N}}\, \Delta H_{i,\mathrm{N}}) / 14.0067\, \Delta \mathrm{t} \qquad \text{Eq. 9}$$

where 14.0067 gN mol^{-1} N is the atomic weight of nitrogen and the time-step is one month. Along with the Ca, Mg, K, and Na ions released from weathering the applied minerals, $\Delta \mathrm{Acidity}_{N}$ contributes a negative term to the soil water alkalinity balance used to calculate the soil pH (Eq. 10):

$$\mathrm{Alk}_{\mathrm{t}} = \mathrm{Alk}_{\mathrm{t-1}} + 2 \cdot (\mathrm{Ca}_{\mathrm{weath}} + \mathrm{Mg}_{\mathrm{weath}}) + \mathrm{K}_{\mathrm{weath}} + \mathrm{Na}_{\mathrm{weath}} - \Delta \mathrm{Acidity}_{\mathrm{N}} \qquad \text{Eq. 10}$$

This pH value is accounted for in the rate laws for mineral dissolution and therefore influences the rate of basalt weathering and the resulting net alkalinity that is produced at each depth within the soil profile, and that contributes to CDR[10,19].

Modelling Soil Profile pH and its Effects on EW. The initial alkalinity profile in each grid cell is determined from the starting soil pH profile[23] downscaled to the resolution of CLM5, and the PCO_2 profile at steady-state based on spin-up of the model with average biomass production and soil organic matter decomposition, which reflects the long-term land use history of a particular location. Alkalinity mass and flux balance for an adaptive time-step accounts for alkalinity and acidity inputs from 1) mineral dissolution rates and secondary mineral precipitation (pedogenic carbonate), 2) biomass production and decomposition[24] and 3) biogeochemical N transformations. The soil pH profile is determined from an empirical soil pH buffering capacity[25] relating soil pH to the alkalinity at each depth. The soil PCO_2 depth profile of a grid cell is generated with the standard gas diffusion equation[26], scaled by monthly total soil respiration from CLM5.

The vertical root respiration profile is based on the monthly total soil respiration from CLM5 for any given grid cell and assumed to be distributed proportionally to the root biomass. A traditional approach is taken to estimate the vertical root distribution by applying an exponential root density profile given a column total[27]. The root density profile is then given by (Eq 11):

$$\rho_{rd}(t,z) = A(t)e^{-az} \qquad \text{Eq. 11}$$

where *z* is the depth; $a = 1/L$, where L is a vegetation dependent e-folding length scale; and *A(t)* is the surface root density. The rooting depth (*d*) is typically defined as the depth that contains 95% of the root biomass, which can be shown to be equal to (Eq. 12):

$$d = \frac{-\ln(1-0.95)}{a} = \frac{3}{a} \qquad \text{Eq. 12}$$

Given that the root respiration (ρ_{rr}) is proportional to the root density and A_{rr} is the monthly total respiration, then (Eq. 13)

$$\rho_{rr}(t,z) = A_{rr}(t)e^{-3z/d} \qquad \text{Eq. 13}$$

Integrating Eq. 13 over the soil depth gives the total root respiration $RR(t) = A_{rr}d/3$. And therefore (Eq. 14)

$$\rho_{rr}(t,z) = \frac{3RR(t)e^{-3z/d}}{d} \qquad \text{Eq. 14}$$

The above source term Eq. 14) is used in a transport equation (Eq. 15) to track the alkalinity contribution from the root respiration.

$$\phi\frac{\delta A}{\delta t} = -q\frac{\delta A}{\delta z} + \frac{3RR(t)e^{-3z/d}}{d} \qquad \text{Eq. 15}$$

At any location, the soil solution is in dynamic equilibrium with dissolved inorganic carbon species and the values of gas phase soil and atmospheric pCO_2. The relative change induced by weathering will be the consumption of H^+ and the production of HCO_3^-.

Extended Data fig. 5 provides an initial evaluation of model performance in a field setting. The comparison indicates modelled EW rates of calcium and magnesium loss from the applied

basalt, soil pH and potential CDR rates within error of observations from an EW field trial in the Corn Belt (2017–2020) lending support to our approach.

Gross CDR calculations. The gross CDR by EW of crushed basalt applied to topsoil was calculated as the sum of two pathways: (1) the transfer of weathered base cations (Ca^{2+}, Mg^{2+}, Na^{+} and K^{+}) from soil drainage waters to surface waters that are charge balanced by the formation of HCO_3^{-} ions and transported to the ocean (equation (16)), and (2) the formation of pedogenic carbonates (equation (17)).

Pathway 1 for calcium ions: $CaSiO_3 + 2CO_2 + 3H_2O \rightarrow Ca^{2+} + 2HCO_3^{-} + H_4SiO_4$ (Eq. 16)

Pathway 2 for calcium carbonate formation: $Ca^{2+} + 2HCO_3^{-} \rightarrow CaCO_3 + CO_2 + H_2O$ (Eq. 17)

Monovalent and divalent base cations are released from basaltic minerals by dissolution based on stoichiometry (**Data File S6**). CDR, via pathway 1, potentially sequesters two moles of CO_2 from the atmosphere per mole of divalent cation. However, ocean carbonate chemistry reduces the efficiency of CO_2 removal (η) to an extent, depending on ocean temperature, salinity and the dissolved CO_2 concentration of the surface ocean. We calculate η for average ocean temperature (17 °C), salinity (35%) and a Representative Concentration Pathway (RCP) 8.5 simulation for 2050 (i.e., the worst-case scenario) for dissolved pCO_2 of 600 μatm, giving η = 0.86 moles of CO_2 removed per mole of monovalent cation and 1.72 moles of CO_2 removed per mole of divalent cation added to the oceans[28]. CDR via pathway 2 occurs if dissolved inorganic carbon derived from atmospheric CO_2 precipitates as pedogenic carbonate and sequestered 1 mol CO_2 per mole of Ca^{2+}. Thus for any given grid cell, we compute CDR by EW as the alkalinity flux in soil drainage and pedogenic calcite precipitation. Possible CO_2 degassing due to changes in surface water chemistry during transport of weathered products draining from soils into large river systems is determined by catchment river modelling, see subsequent sections.

Quarrying and Grinding Costs and Energy

Energy and financial costs associated with the extraction of rock (**Tables S1, S2**) were derived from ref[29], for which we assume the energy costs associated with the detailed UK case-study are consistent with those in the U.S. The framework for technoeconomic assessment was adapted for EW from ref[30]. Operating labour and supervision were calculated using working hours from ref[29] and US average salaries. Capital and operational costs for crushing were converted from $£_{2010}$ to $\$_{2020}$ values using an average 2010 exchange rate of 1.546$ to £, a capital location factor for Western Europe of 1.06, and the Chemical Engineering Plant Cost Index (551 in 2010, and 596.2 in 2020). Although missing from ref[29], we assumed an engineering, procurement, and contractors cost of 8% and a contingency of 5% (given that rock extraction is a highly mature and globally scaled industry). Additional owners' costs of 20% total plant costs (not included in ref[29]) were also incorporated (**Tables S1, S2**).

Rock grinding is calculated by scaling the cost of a semi-autogenous mill (i.e. uses feed rock as the grinding medium): reference cost $1.7M for 300 kW and scaling exponent of 0.31, and a motor: reference cost $260k for 2,225 kW and a scaling exponent of 0.81, both to a maximum of 4,500 kW with a labour and maintenance factor of 2, ref[31]. Bare erected costs were calculated using the Chemical Engineering Plant Cost Index (1000 for the reference, and 596.2 in 2020). For total power requirements greater than the maximum of a single unit, multiple fully scaled units were

used. We assumed an engineering, procurement, and contractors cost of 8% and a contingency of 5% (given that rock extraction is a highly mature and globally scaled industry). Additional owners' costs of 20% total plant costs were also incorporated (**Table S3**).

The levelized cost of production (LCOP) was calculated using the method detailed in ref[32], assuming a capital charge factor (CCF) of 0.11. Capacity factors (CF) were assumed to already be accounted for in production data. We calculated a levelization factor (LF, ~1.24) assuming rates of 12% discount, 2% inflation, 1% escalation, and a 25-year capital service life. All costs (labour, electricity, diesel) reflect $\$_{2020}$, averaged over the U.S. To obtain state-specific costs over the 2020–2070 period, we averaged and normalized E3-US price projections for each state for the year 2020 and scaled accordingly (**fig. S7**).

Grinding energy requirements E in kWh/t were obtained from ref[33] as:

$$E = 4.76 \times M \times \left(\frac{1}{x_{prod}^{f(x)}} - \frac{1}{x_{feed}^{f(x)}}\right) \quad \text{Eq. 18}$$

where M is the work index (assumed 19.4 kWh/t), x is the p80 in μm of the feed or product (in this study 100 μm), and $f(x) = -0.295 + \left(\frac{x}{10^6}\right)$. Electrical energy requirements of grinding were 16.6 kWh/t and total requirements for extraction and comminution were 20.4 kWh/t.

Transportation Costs and CO_2 Emissions

We analyzed transportation costs and CO_2 emissions as described previously[2,23,28]. We estimated costs and emissions for basalt transport by assimilating projected data of fuel/electricity prices, wages (**figs. S7, S8**), as well as the electricity production mix and the corresponding emission factors at a state level using data from the E3-US macro-economic model (**figs. S9, S10**)[34]. Fuel efficiency, for freight road transport, was 2.1 kWh/mile (1.3 kWh/km) for heavy electric vehicles[35,36]. For heavy diesel fuel trucks in 2020, we used 5.8 miles per US gallon (2.51 km/litre)[37]. For freight rail fuel efficiency in the United States we used data from historical records at 472 t-miles per gallon[38,39].

To estimate transport costs per t-kilometer (t-km), we considered standard road and rail cost models, including wages, fuel, maintenance, and depreciation[40,41]. For roads, we consider a heavy transport type (33 tons with a payload capacity of 22 tons) and, in-line with emissions accounting for freight operations, assume road transport vehicles will perform a different activity after basalt delivery[42] (i.e., single trip). Modifying our analysis to include costs for a return journey increases CDR costs by ~15% for the first decade of deployment and much less thereafter with the electrification of the transport network. We assumed that electric HGV will be readily available from 2030, achieving a 90% market share by 2050[34,36,43]. We modelled the rail network decarbonization transition by adding a 20-year lag to the electric HGV scenario[39] (**fig. S8**). We then applied a projected shared pathway of both transport types to estimate transport costs. Transport distances were calculated from geographical data to identify available sources of basalt rock[44] and cropland areas using a transport network analysis with least cost path algorithms in ArcGIS[45]. Finally, we utilized a combination of truck and train modes for transporting basalt to the crops, depending on the distance from the rock source. For distance bands ranging from 50 km

to 1750 km, the proportion of truck usage varied from 0.96 to 0.31, respectively[46]. This reflects rail transport being more cost effective than trucks over longer distances. Costs and carbon emissions of both transport modes will fall in the coming decades, with the introduction of electric HGV but with a slower rollout of the electrification of the rail network[37]. Emissions and costs for rock dust spread were obtained following[6] adapted for the U.S. following E3ME model outputs.

U.S. Energy and economic forecasts

Projections of costs are based on a global policy scenario consistent with limiting global warming above pre-industrial level to 1.5°C (without overshoot), based on population and economic development consistent with SSP2[47]. The scenario is an updated version of that used in ref[48] using the non-optimization integrated assessment model framework E3ME-FTT-GENIE[49], covering global macroeconomic dynamics (E3ME), S-shaped energy technological change dynamics (FTT), and carbon cycle and climate system (GENIE). The scenario incorporates a range of market-based and regulatory policies, including carbon pricing, energy efficiency standards and incentives for low- and zero-carbon technology uptake in the power sector, for personal transport and buildings. It also includes an estimate of the short-term impacts of the Covid-19 pandemic on the economy and energy system. Most of the climate-related policies employed are already implemented in at least one jurisdiction, and here we assume they are implemented in all nations (which is necessary to meet the temperature target). The resulting modelled global greenhouse gas emissions reach net-zero shortly after 2050, driven by electrification of secondary energy use and near-complete decarbonisation of the power sector, which also features some bioenergy with carbon capture and storage to compensate remaining emissions, mostly from agriculture and industrial processes. Bioenergy use is also increased in aviation and freight transport, but global (modern plus traditional) bioenergy consumption does not exceed 150 EJ in any year.

The scenario is disaggregated to state resolution for the USA using data and projections from the E3-US model[50,51] (**fig. S7-S10**). E3-US follows the approach used in the global E3ME model, including high disaggregation into seventy econometrically specified sectors defined in each state. E3-US also incorporates an integrated treatment of the energy system, including a bottom-up representation of the power sector. The model was designed to address questions relating to fiscal policy and redistribution across states, including carbon taxes and other environmental fiscal reform and the impacts of new energy regulation, energy efficiency measures, feed-in-tariffs and support for new technologies.

Prices at state level are estimated by mapping the national-level figures for the U.S. to the state-level data, while maintaining overall consistency with national outcomes at sectoral level. For example, a RAS procedure ensures that the volume of electricity generated by each technology in each of the US's three grids sums to the national total, while still meeting the expected levels of electricity demand. Electricity prices in each state are then determined by taking a weighted average of the levelized costs of each generation technology used in the relevant grid, adjusting for state taxes.

Modelling EW Effects on Riverine Carbonate Chemistry

Our model for estimating carbonate saturation state (CSS) in the rivers uses well-documented aqueous geochemical datasets in the contiguous U.S., where United States Geological Survey (USGS) has been monitoring the solute composition and discharge rates of the US streams with a high temporal resolution and a high spatial coverage for many decades[52]. From the USGS, we

collected a suite of aqueous species and parameters, including alkalinity, pH, calcium, water temperature, salinity, and discharge rate. Sites with multiple measurements in a day are averaged daily for each parameter, after which all parameters are merged together based on the unique site number and sampling date and any rows containing NA values are deleted. After data filtering, the average monthly value of each parameter for each river is calculated by aggregating the samples by month using a discharge-weighted approach. Also note that for pH values, we first converted original pH values from –log10([H^+]) scale to the [H^+] concentration scale (mol/L) before data aggregation and then converted them back to –log10([H^+]) in the final step for calculating river omega values.

We then delineate the corresponding watershed for each river site using GRASS GIS[53] based on the stream flow direction at a 30" resolution from HydroSHEDS[54]. Specifically, we use the GRASS module r.accumulate to derive the stream network and flow accumulation over the northern America from stream direction. Next, we snapped each river gauge to the newly calculated stream network. We then use the module r.water.outlet to create the watershed basin for each snapped stream gauge. The watersheds that are completely located within the states where EW is implemented are selected, which are used to calculate the time-series water infiltration flux (from climate model data) that drains into each river site. Finally, we have a dataset containing 863 river sites (**Fig. 2a**) with complete hydrogeochemical parameters and watershed boundaries that will be fed into the carbon saturation state (CSS) model to calculate the initial Ω values. Using the HydroSHEDS stream network, we also delineate 6 large watersheds (including Mississippi, Colorado, Columbia, Sacramento, Lawrence, and Nelson) in North America (**Fig. 2b**). We then calculate the fluxes of bicarbonate, Mg, Ca, Na, K, Fe, and Si (derived from EW modeling) for each big watershed through time (**Fig. S11, S12**). Some of these fluxes will be fed into the ocean model to estimate the impact of ocean carbon storage on CDR efficiency (see next section).

Initial and time-series Ω values during EW are calculated based on solute chemistry, temperature, and salinity according to (Eq. 19):

$$W = \frac{[Ca^{2+}][CO_3^{2-}]}{K_{sp}} \qquad \text{Eq. 19}$$

where K_{sp} represents the apparent solubility product for calcite corrected for site-specific temperature and salinity[55] and brackets denote concentration.

We first calculate dissolved inorganic carbon (DIC), [CO_3^{2-}] and K_{sp} using the seacarb package in R[56], considering the impact of both temperature and salinity. Combining [CO_3^{2-}], K_{sp} and [Ca^{2+}], we calculate the initial Ω for each site. The distribution of Ω in rivers/streams is right-skewed, and the majority of the Ω values are smaller than 10. To model the evolution of monthly Ω values in each river site through time, we first used linear interpolation to build the relationship between the monthly background concentration of aqueous species (including Ca, DIC, alkalinity, and salinity) and river discharge for each single watershed. Then we used this relationship to calculate the monthly concentration of these aqueous species in each river site scaled by the water infiltration rate (derived from CLM5) through time. Note that these concentration values are treated as the new background values in the river before receiving the EW products. Afterwards, we calculate the extra concentrations of Ca, alkalinity, and salinity generated by EW at each watershed for each month through time and added those extra concentrations to the corresponding background aqueous species concentration to obtain the updated river chemistry (**fig. S13**).

Finally, we calculate the new Ω values using DIC, alkalinity, Ca concentration, salinity, and water temperature through time following the method of Zhang et al.[57] For both the 1 Gt yr^{-1} and 2 Gt yr^{-1} rock extraction scenarios, most river sites experience Ω values smaller than 10 from 2020 to 2070. This highlights that the transport of dissolved constituents in surface waters is unlikely to be a primary bottleneck limiting the CDR potential of EW.

Estimating the impact of ocean carbon storage on CDR efficiency

We evaluate the CDR efficiency by EW on the U.S. croplands using a 'carbon-centric' version of the Grid Enabled Integrated Earth system model — cGENIE. The ocean physics and climate model components of cGENIE comprise a reduced physics (frictional geostrophic) 3-D ocean circulation model coupled to a 2-D energy-moisture balance model and a dynamic-thermodynamic sea ice model[58]. Heat, salinity, and biogeochemical tracers are transported via parameterized isoneutral diffusion and eddy-induced advection[59]. The ocean model exchanges heat and moisture with the atmosphere, sea ice, and land while being forced at the ocean surface by zonal and meridional wind stress according to a specified static wind field. Heat and moisture are horizontally mixed throughout the atmosphere and exchange heat and moisture with the ocean and land surfaces, with precipitation occurring above a given relative humidity threshold. The sea ice model tracks horizontal ice transport and exchanges of heat and fresh water, using the thickness, areal fraction, and concentration of ice as prognostic variables. Full descriptions of the climate model and ocean physics can be found in[58,60]. The ocean model is configured here as a 36 × 36 equal-area grid (uniform in longitude and sine of latitude) with 16 logarithmically spaced depth levels and seasonal forcing at the ocean surface.

The ocean and sediment biogeochemistry modules in cGENIE control air-sea gas exchange, the transformation and repartitioning of biogeochemical tracers within the ocean, and the impacts of shallow sediment diagenesis on calcium carbonate formation/dissolution and burial. The ocean biological carbon pump is driven by a parameterized uptake rate of nutrients in the surface ocean, with this flux converted stoichiometrically to biomass that is then partitioned into particulate or dissolved organic matter for downstream advective transport, sinking, and remineralization within the ocean interior. Dissolved organic matter is transported with the ocean circulation and decays according to a specified time constant, while particulate organic matter is instantaneously exported from the surface ocean and is remineralized within the ocean interior following an exponential decay function with a specified remineralization length scale. The ocean biogeochemistry also contains a fully coupled carbonate system, which tracks individual dissolved inorganic carbon (DIC) species, dissolved alkalinity, and ocean pH. Calcium carbonate forms in surface ocean grid cells at a stoichiometric ratio with organic matter production (the so-called "rain ratio") and is exported as a solid species and is dissolved in the ocean interior or shallow marine sediments depending on ambient temperature, pressure, and carbonate chemistry[122]. A simple scheme for shallow sediment diagenesis allows us to run the ocean alkalinity cycle as an open system, with delivery from weathering of the land surface and ultimate burial as calcite ($CaCO_3$) in marine sediments). More detailed description and validation of the ocean and sediment biogeochemistry in cGENIE is provided in refs. [59,61]. We implement a simple model of carbon exchange with the terrestrial biosphere in which aboveground biomass (vegetation) and soil carbon are treated as global pools that respond to temperature and atmospheric pCO_2 (e.g., a "slab" or "box" terrestrial biosphere)[62].

The model climate system and ocean carbonate/alkalinity cycle are spun up to steady state using a two-stage procedure. First, the model is run as a closed system for 20 kyr with atmospheric abundances of CO_2, CH_4, and N_2O imposed at preindustrial values to bring the ocean-atmosphere system and shallow sediments into steady state. This run is used to diagnose the approximate steady state burial flux of calcium carbonate in marine sediments, which is then imposed as a weathering flux of calcium and alkalinity in a second stage spin up in which the ocean and sediments are allowed to evolve as an open system. The second stage spin up is run for 20 kyr.

All subsequent simulations are branched from the open system spin up at model year 1765 and run to year 2100 according to the SSP2-4.5 scenario for atmospheric CO_2, CH_4, and N_2O[63]. Time-varying atmospheric abundances of CH_4 and N_2O are imposed according to the SSP2-45 trajectory for all simulations, while atmospheric CO_2 abundance is emission-driven. The emission trajectory for SSP2-4.5 is first computed by the model by prescribing the atmospheric CO_2 trajectory for that scenario, with all subsequent runs utilizing the emission trajectory diagnosed in cGENIE for the SSP2-4.5 pathway.

Our simulations of carbon dioxide capture through EW on croplands in the U.S. reflect riverine delivery of alkalinity (here assumed to be the sum of Mg and Ca) and DIC from 6 watersheds from U. S. riverine data. The riverine alkalinity and DIC flux data are averaged every 5 years and fed to 8 consecutive cGENIE runs from 2030 to 2070 as the forcings of DIC and alkalinity fluxes at the surface ocean grid cells corresponding to 6 river mouths (**fig. S14**) in addition to the CO_2 removal flux from the atmosphere which corresponds to the HCO_3 fluxes (**fig. S11**).

We also simulate a hypothetical carbon cycle intervention meant to represent direct CDR or mitigation of emissions more than that implied by SSP2-45 pathway. For this, we reduce CO_2 emission rates by a specified value corresponding to EW in the U.S. croplands except without any addition of alkalinity and DIC to the ocean, relative to the control emission rates for SSP2-45. This simulation is referred to as "baseline" or "base" CDR experiment. By comparing CO_2 leakage between "EW" and "baseline" experiments we can evaluate the leakage specifically caused by addition of DIC and alkalinity to the ocean through EW.

Air Quality Simulations

We assessed the effect of changes in soil nitrogen emissions from EW on air quality, using CESM2.2. For the atmospheric component, we employed CAM-chem version 6 with the MOZART Troposphere Stratosphere (TS1) chemistry mechanism[64] with the Model for Simulating Aerosol Interactions and Chemistry (MOSAIC) with the four-mode Modal Aerosol Mode (MAM4). The MOSAIC is a comprehensive aerosol chemistry module designed to simulate aerosol concentrations and size distribution, including nitrate aerosol. MOSAIC dynamically partitions H_2SO_4, methanesulfonic acid (MSA), HNO_3, HCl, NH_3, and secondary organic aerosol (SOA) into different aerosol size bins/modes, driven by particle-phase thermodynamics and heterogeneous chemical reactions. The formation of HNO_3 is through O_3-NO_x-HO_x chemistry or N_2O_5 hydrolysis, which are calculated in the TS1 chemistry[65]. CAM6-chem is coupled to the interactive CLM5, which provides biogenic volatile organic compounds (BVOCs) emissions and handles dry deposition.

We performed two sets of experiments: Control (present-day climate and future emissions with no basalt application) and EW (present-day climate and future emissions with basalt application). For future anthropogenic and biomass burning emissions, we used the inventory developed for CMIP6 SSP2-4.5[66]. Soil agriculture NO and NH_3 emissions were obtained from our CLM5 EW 2

Gt yr^{-1} modelling experiment (by 2030, 2050 and 2070). **Table S4** summarizes the main anthropogenic emissions for short-lived air pollutants projected over the United States, and the soil agriculture NO and NH_3 emissions. For climate, we ran CAM6-chem nudged to the Modern-Era Retrospective analysis for Research and Applications (MERRA2) meteorological fields from 2015 to 2018[67] to reduce variability related to dynamic simulations in the atmospheric model (CAM6) and fully focus on surface O_3 and $PM_{2.5}$ air quality changes from EW. This setting also ensures that BVOC emissions remain the same in both experiments, thereby reducing the influence of potential future changes in biogenic emissions on our air quality assessment. Additionally, we kept the fire module off, i.e., we used prescribed biomass burning emissions, as well as the ozone damage module off, to minimize interference from other processes. We note that this approach may introduce uncertainties as future changes in climate, BVOCs emissions, fires and ozone uptake by plants could alter atmospheric chemistry and air quality outcomes. All simulations ran for 4 years, with the first year as model spin-up, for 2030, 2050 and 2070 emission conditions. Simulations were performed at the horizontal resolution of 0.9° × 1.25° (about 100 km × 100 km). CESM2 simulations have been extensively evaluated by comparison with satellite, sonde, aircraft and ground observations on regional and global scales (e.g., refs[64,68,69]).

Air pollution effect on crop yields

We investigated the effects of projected changes in surface O_3 levels with EW on crop yields using three different ozone exposure metrics widely used to quantify vegetation ozone exposure and protect crop health[70]. They included a cumulative exposure metric (AOT40; ppm-hr), a mean exposure metric (M12 or M7; ppb) and a flux-based approach (POD_3; mmol m^{-2}) (**Table S5**). In CESM2.2, F_{O_3} used to calculated POD_3 is calculated using the O_3 concentration (nmol m^{-3}) and the O_3 dry deposition velocity (s m^{-1}), which depends on the aerodynamic, quasi-laminar sublayer about canopy and the surface resistances[71]. The surface resistance is computed based on the stomata and leaf mesophyll resistances, which considers the stomatal control of O_3 uptake and its environmental dependances (e.g., soil water availability, atmospheric CO_2, etc). We considered June–August as the growing period for maize, soybean and wheat in the USA.

The relative yield (RY) for each crop was then derived from statistical relationships between historical ozone exposure and crop yields observations for maize, soybean and wheat (**Table S5**). To estimate the economic gains from avoided surface O_3 pollution (Fig. 4), we used the RY changes estimated from our three ozone exposure metrics and per state datasets[72] for crop yield (BU/acre), price ($/BU) and area (acre) for maize, wheat and soybean reported in 2020. Crop prices were adjusted to consider market changes in 2030, 2050 and 2070 using the FAO market producer price indexes[73].

Table S1. Costs for mineral extraction and crushing to aggregate (mm) sized particles for a 1.3 Mt/yr operation. (adapted from ref[29] for N. America using the method described above).

**Cost item**	**Value**
**Operational Costs**	
Total operating labor + supervision ($\$_{2020}$/yr)	1,038,332
Administration ($\$_{2020}$/yr)	51,917
Fixed plant operation ($\$_{2020}$/yr)	896,066
Building operation ($\$_{2020}$/yr)	101,155
Equipment operation ($\$_{2020}$/yr)	106,714
Tyres ($\$_{2020}$/yr)	25,187
Total fixed operating cost ($\$_{2020}$/yr)	*2,219,370*
Diesel (litre/day)	266
Electricity (kWh/day)	19,888
Total variable operating cost ($\$_{2020}$/yr)	*377,945*
**Capital Costs**	
Primary ($\$_{2020}$)	415,591
Secondary ($\$_{2020}$)	15,283,860
Tertiary ($\$_{2020}$)	17,381,167
Screening ($\$_{2020}$)	11,197,023
Buildings ($\$_{2020}$)	4,651,038
Development ($\$_{2020}$)	821,358
Mobile Plant ($\$_{2020}$)	13,902,445
Total Bare Erected Costs ($\$_{2020}$)	63,652,481
Engineering, procurement, and contractors ($\$_{2020}$)	5,092,198
Contingency ($\$_{2020}$)	3,437,234
Total Plant Costs ($\$_{2020}$)	72,181,914
Total Overnight Costs ($\$_{2020}$)	*86,618,297*

Table S2. Additional cost items associated with rock grinding to 100 µm for a 1.3 Mt/yr operation. Electricity requirements were derived from Morell[33], total free on board equipment costs were derived from Woods[31], and total overnight costs calculated according to the method outlined above.

**Cost item**	**Value**
**Operational Costs**	
Electricity (kWh/t)	16.6
Electricity ($/yr)	1,441,667
Maintenance ($/yr)	115,288
Total additional opex ($/yr)	1,556,995
Total Power (kW)	4,138
**Capital Costs**	
Total Free on board ($,CEPCI:1000)	4,263,031
Total Bare erected ($_{2020}$)	5,083,238
Engineering, procurement, and contractors ($_{2020}$)	406,659
Contingency ($_{2020}$)	274,495
Total Plant Costs ($_{2020}$)	5,764,392
Owners costs (20% TPC)	1,152,878
Total Overnight Costs ($_{2020}$)	*6,917,271*

Table S3: Summary of the levelized costs for mineral extraction and processing to 100 µm in North America for a 1.3 Mt/yr operation (adapted from ref[29] for North America using the method described above).

**Cost item**	**Value**
Total Overnight Costs Crushing ($_{2020}$)	*86,618,297*
Total Overnight Costs Grinding ($_{2020}$)	*18,355,997*
Levelized capital cost of production ($/t)	9.8
Fixed operational costs ($_{2020}$)	2,334,657
Levelized fixed operational costs of production ($/t)	2.2
Total Electricity (kWh/t)	20.4
Total Diesel (L/t)	0.05
Diesel ($/litre)	0.674
Electricity ($/kWh)	0.067

Table S4. Summary of future anthropogenic and soil agriculture emissions in the U.S.[a]

Anthropogenic							Soil Agriculture			
							No EW		EW (2 Gt yr^{-1})	
Year	BC	OC	CO	NO[b]	NH_3[b]	SO_2	NO	NH_3	NO	NH_3
2030	0.12	0.59	16	1.7	0.19	0.37	0.44	1.81	0.43	1.80
2050	0.08	0.45	12	1.1	0.17	0.20	0.46	1.90	0.38	1.95
2070	0.05	0.54	8	0.7	0.15	0.15	0.47	2.00	0.35	2.05

[a]Total annual emission. BC, OC, CO: Tg C yr^{-1}; NO and NH_3: Tg N yr^{-1}; SO_2: Tg S yr^{-1}

[b]Anthropogenic NO and NH_3 do not include agriculture emissions.

Table S5. Summary of ozone exposure metrics and relative yields for maize, soybean and wheat.

Ozone Exposure Metrics[a]	Relative yield (RY) for each crop	References
$AOT40 = \sum_{i=1}^{n}([O_3]_i - 0.04)$	RYmaize = 1 − 0.0124 × AOT40<br>RYsoybean = 1 – 0.0090 × AOT40<br>RYwheat = 1 – 0.0163 × AOT40	ref[74]<br>ref[75]
$M12 \text{ or } M7 = \frac{1}{n}\sum_{i=1}^{n} 10^3[O_3]_i$	$RYmaize = \frac{e^{-\left(\frac{M12}{124}\right)^{2.83}}}{e^{-\left(\frac{20}{124}\right)^{2.83}}}$<br>$RYsoybean = \frac{e^{-\left(\frac{M12}{107}\right)^{1.58}}}{e^{-\left(\frac{20}{107}\right)^{1.58}}}$<br>$RYwheat = \frac{e^{-\left(\frac{M7}{186}\right)^{3.2}}}{e^{-\left(\frac{25}{186}\right)^{3.2}}}$	ref[76]<br>ref[76]<br>ref[77]
$POD_3 = 10^{-6}\sum(F_{O_3} - Y)\,\Delta t$	RYmaize =1-0.0041 × POD_3<br>RYsoybean = 1-0.0067 × POD_3<br>RYwheat =1-0.0064 × POD_3	ref[78]<br>ref[78]<br>ref[78]

[a]$[O_3]_i$ is the hourly mean ozone mixing ratio in ppm during the twelve hours of local daylight (08:00–20:00); n is the number of hours in the 3-month growing season defined as the three months prior to the start of the harvest period for each crop; Y is the threshold (3 nmol O_3 $m^{-2}s^{-1}$), Δt is the time step (in s) and F_{O_3} is the O_3 flux (nmol O_3 m^{-2} s^{-1}) minus the threshold accumulated over a 90-day growing period. M7 is computed similarly as to M12 but considering a 7-hr day time window (9:00-4:00).

(a) Western U.S. (n = 7988) **(b)** Eastern U.S. (n = 341)

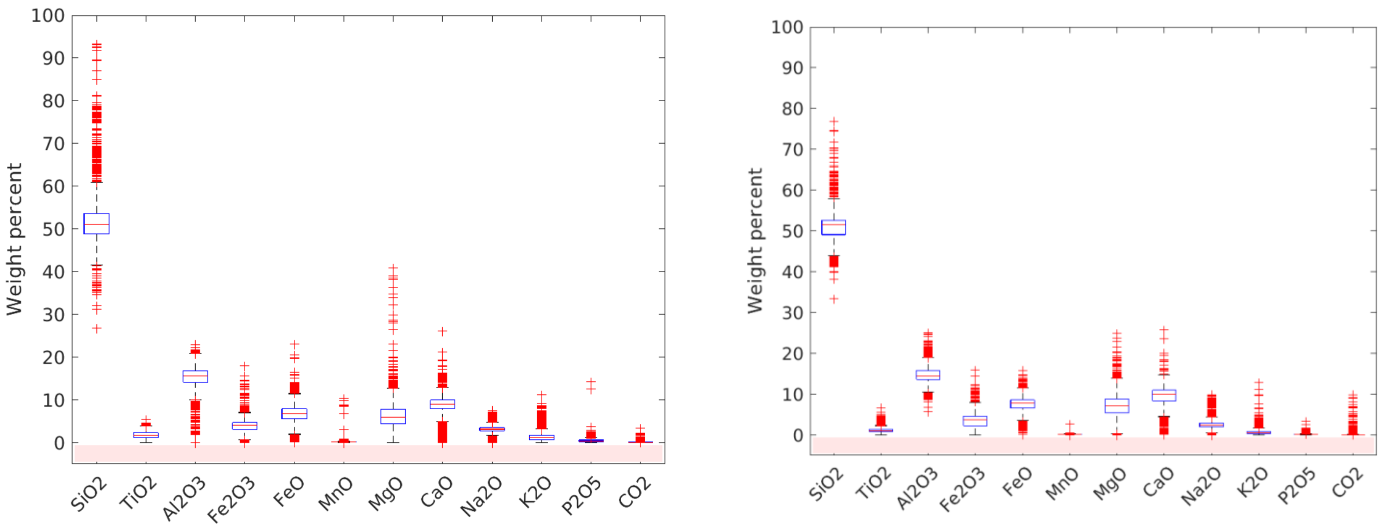

Fig. S1. Basalt tock metal oxides in western and eastern regions. Box and whisker plots of the metal oxide datasets for mafic rocks in (**a**) western and (**b**) eastern U.S. Eastern points are east of –90E and western points are west of –100E. The boxplots of the oxide data suggest only minor differences between east and west coast mafic rocks, with western rocks showing a slightly greater range of values for the important plant nutrients K and P. We use three K-bearing normative minerals in the scheme: orthoclase (K-feldspar), kalsilite and leucite. Of these, normative orthoclase is nonzero for 9140 samples, normative leucite for only one sample, and no samples had normative kalsilite. As orthoclase is a slow-weathering mineral and non-normative phases such as glasses are excluded, our K release is likely to be conservative. Apatite is the only normative P-bearing mineral; it is non-zero for 8752 samples.

(A) Phosphorus (P) content of U.S. basalts

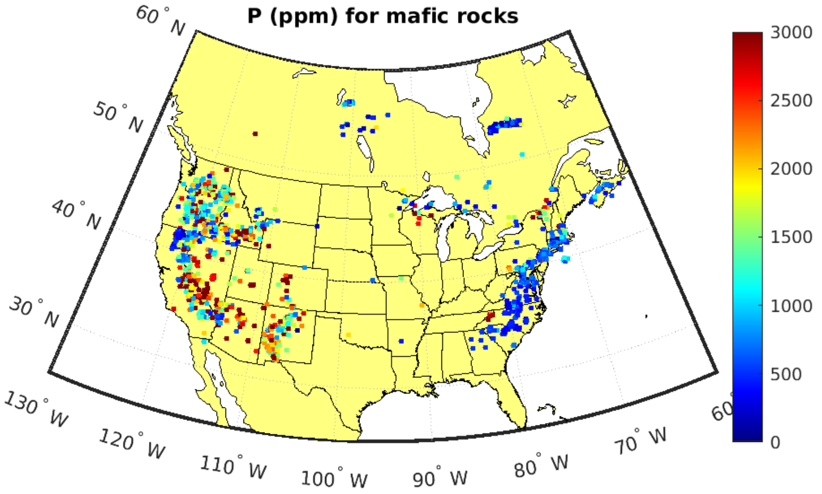

(B) Potassium (K) content of U.S. basalts

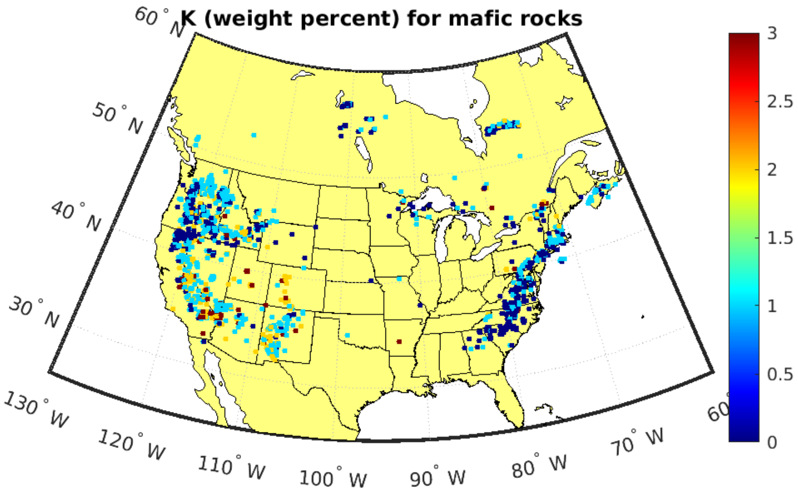

Fig. S2. Geospatially mapped basalt phosphorus (P) and potassium (K) contents Locations for geochemical data used in the present study.

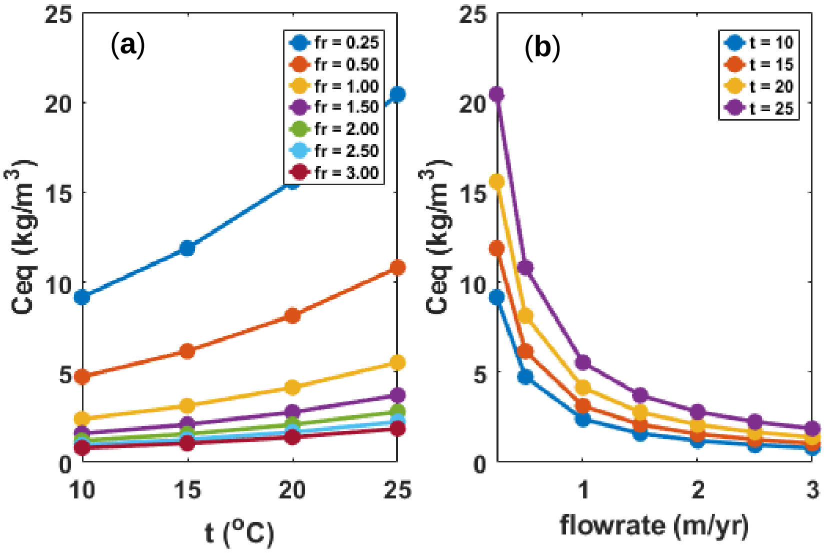

Fig. S3. Variations in the chemical affinity term, C_{eq}, with soil temperature and infiltration rate. (**a**) Response of C_{eq} values calculated by the PHREEQC Reactive Transport Model to increasing soil temperature with varying infiltration rates (flow rate). (**b**) Response of C_{eq} values calculated by the same PHREEQC Reactive Transport Model to increasing soil water infiltration rate (flow rate) with varying soil temperatures. These examples are for the basaltic mineral labradorite.

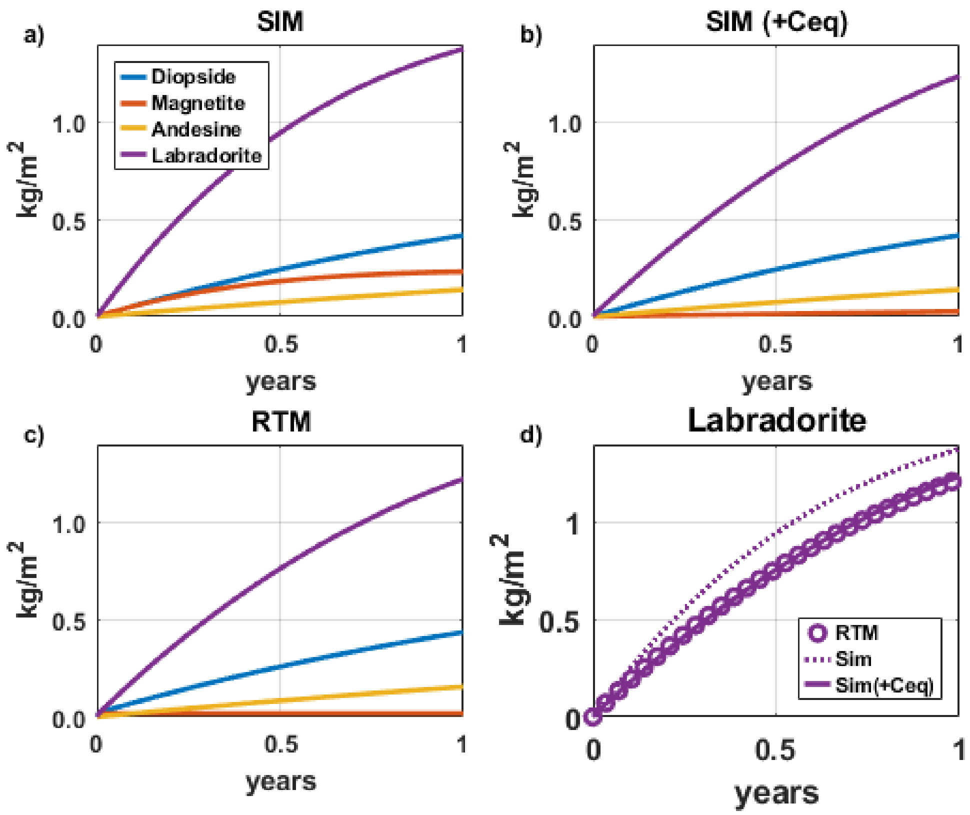

Fig. S4. Comparison of EW model basalt mineral weathering rates 1. Increases in basaltic mineral mass loss by weathering simulated by (**a**) 1-D soil model without C_{eq} applied (SIM), (**b**) the 1-D soil model with C_{eq} applied (SIM + C_{eq}) and (**c**) PHREEQC Reactive Transport Model (RTM). (**d**) Comparison of labradorite mass loss with the 1-D soil model with (SIM + C_{eq}), without C_{eq} (SIM) and the RTM. Note convergence in mineral mass loss by weathering between 1-D soil model with chemical affinity (SIM + C_{eq}) and the RTM.

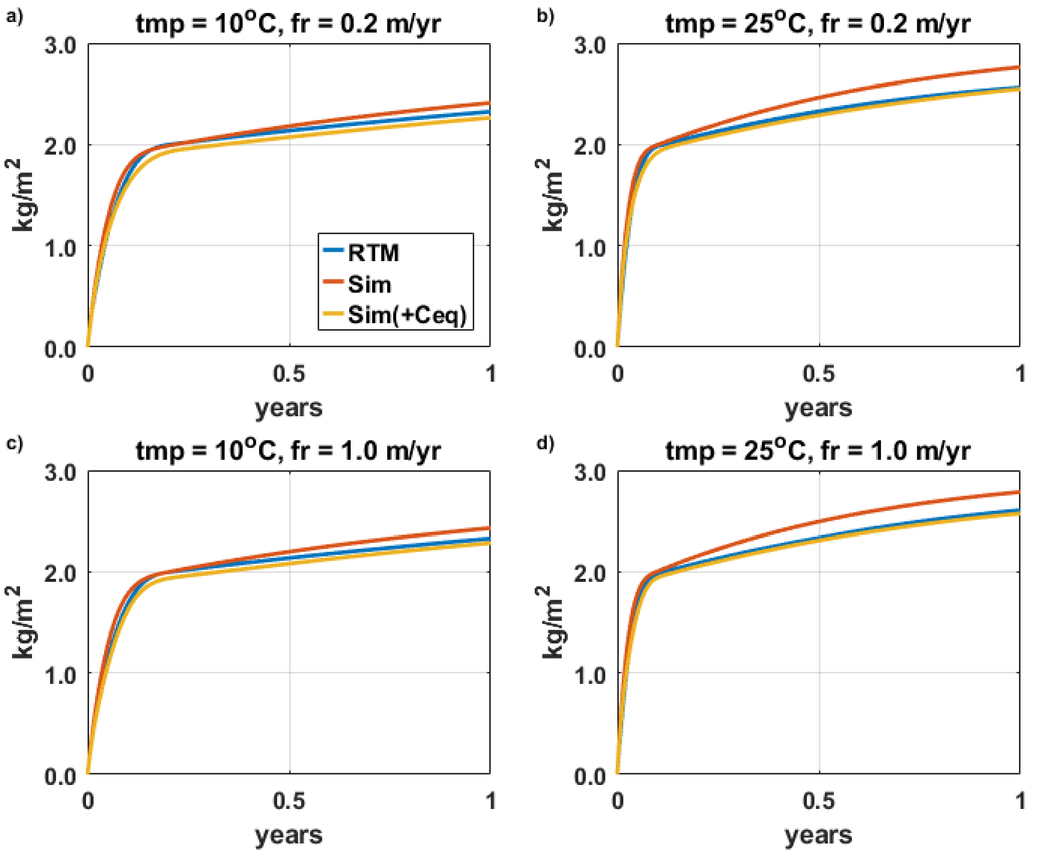

Fig. S5. Comparison of EW model basalt grain weathering rates 2. Each panel (**a**) to (**d**) shows our 1-D soil profile EW model results with (SIM + C_{eq}), and without C_{eq} applied (SIM), and those for the PHREEQC Reactive Transport Model (RTM) over a year at a specific soil temperature (tmp) and infiltration (fr) rate. Note convergence in basalt grain mass loss by weathering between the 1-D soil model with chemical affinity (SIM + C_{eq}) (yellow line) and the RTM (blue line) in all cases. Simulations for New Jersey 2 normative basalt mineralogy, a fast-weathering basalt (Extended Data fig. 3).

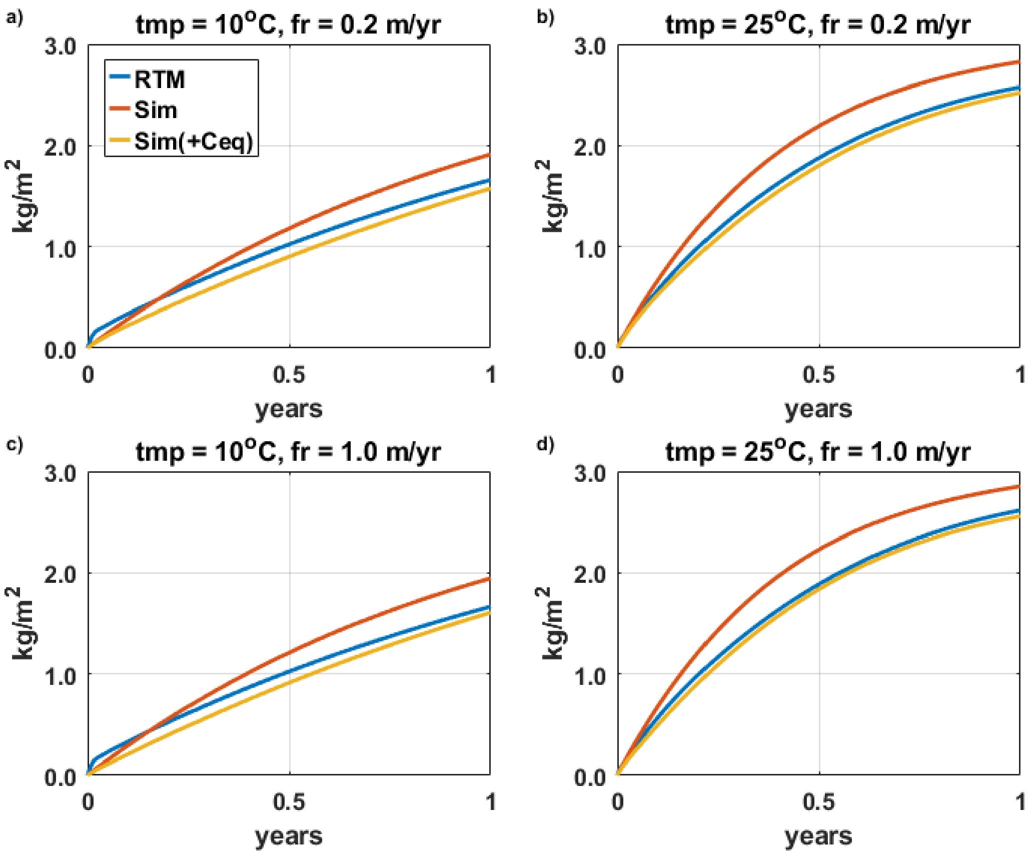

Fig. S6. Comparison of EW model basalt grain weathering rates 3. Each panel shows our 1-D soil profile EW model results with (SIM + C_{eq}), and without C_{eq} applied (SIM), and those for the PHREEQC Reactive Transport Model (RTM) over a year at a specific soil temperature (tmp) and infiltration (fr) rate. Note convergence in basalt grain mass loss by weathering between the 1-D soil model with chemical affinity (SIM + C_{eq}) (yellow line) and the RTM (blue line) in all cases. Simulations for New Jersey 1 normative basalt mineralogy, a slower-weathering basalt (Extended Data fig. 3).

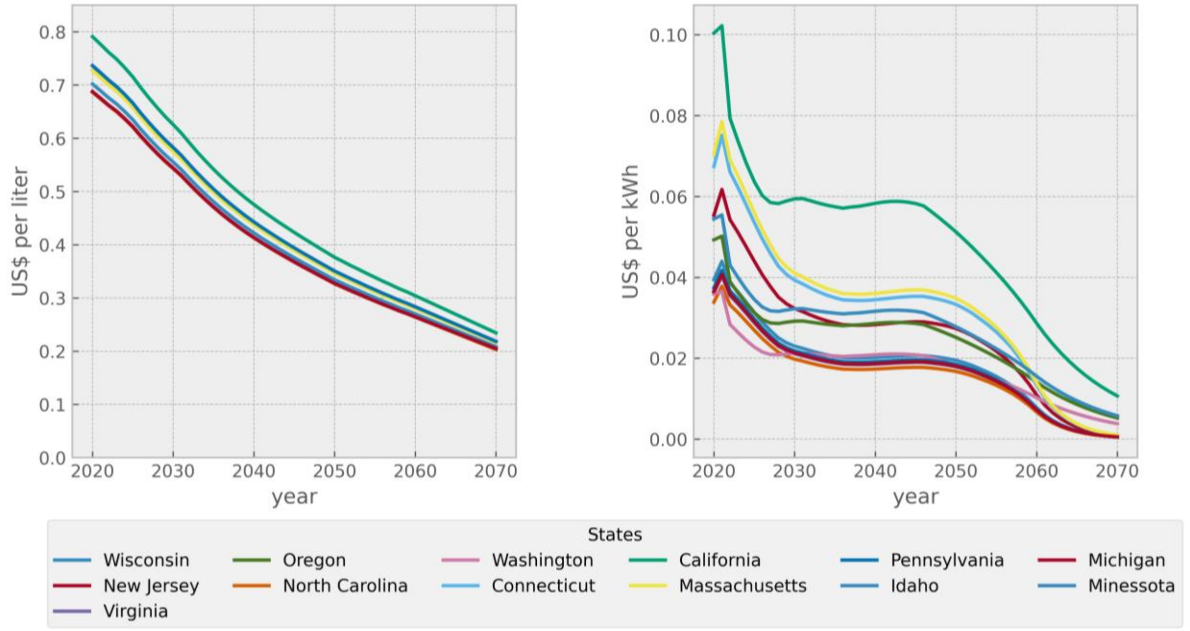

Fig. S7. Projected future changes in energy prices by state. (**a**) Fuel prices in US dollars per litre of diesel, and (**b**) electricity prices in US dollar per kwh (projects from E3-US).

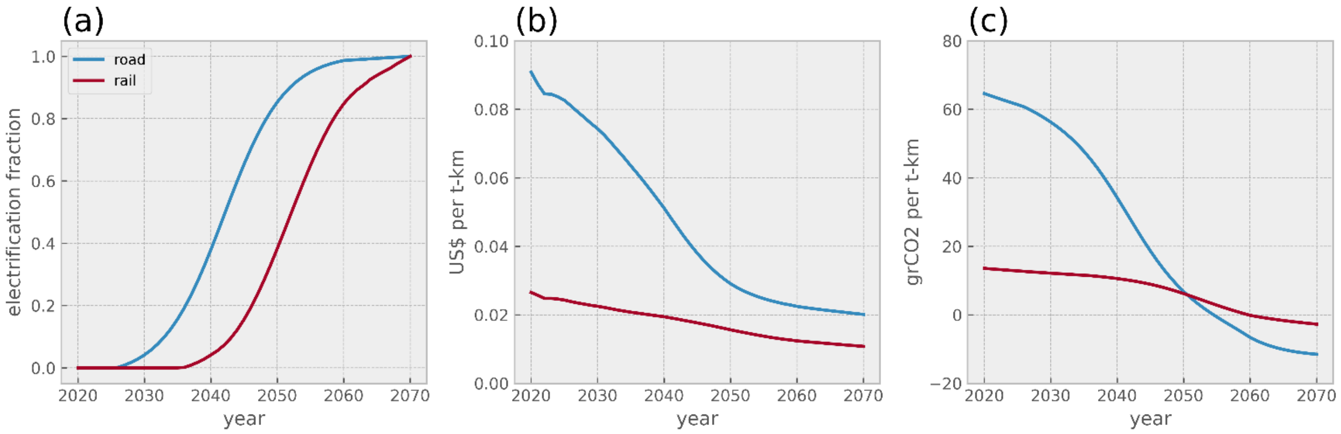

Fig. S8. Transport electrification paths, costs and emissions (**a**) Freight transport electrification path in the US (2020–2070) for both road and rail systems. (**b**) Projected transport cost in US dollars per ton km (tkm) of basalt powder for both road and rail transport modes. (**c**) Projected transport emissions in grCO_2 per tkm of basalt powder for both road and rail transport modes. For electrification of transportation systems, we used the 1.5°C energy policies scenario of the E3-US macro-economic model, which are in line with the Biden "Pathways to Net-Zero Greenhouse Gas Emissions by 2050" report. In that scenario, private vehicle electrification achieves 99% by year 2050 following a logistic growth. To account for challenges in electrifying larger scale transport modes, we added a 10-year lag for heavy duty vehicles and a 20-year lag for rail transportation and the farming machinery used for spreading. In this scenario, heavy duty vehicles achieve an 80% electrification rate by 2050 while the rail network and the spreading equipment reaches 40%. For an equal proportion of road and rail transportation, this would equate to 60% electrification of the transport network by 2050.

.

(**a**) US energy regions

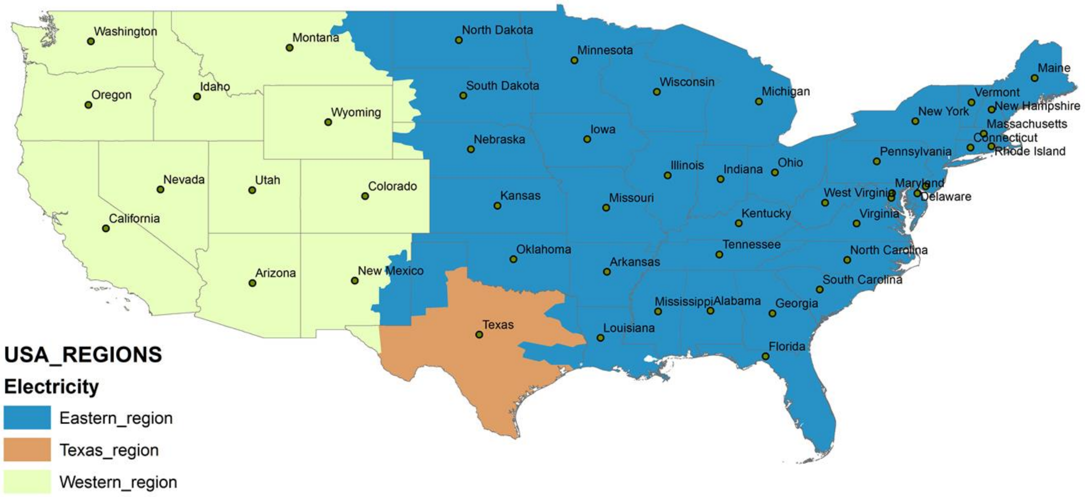

(**b**) Project energy production by region (E3-US)

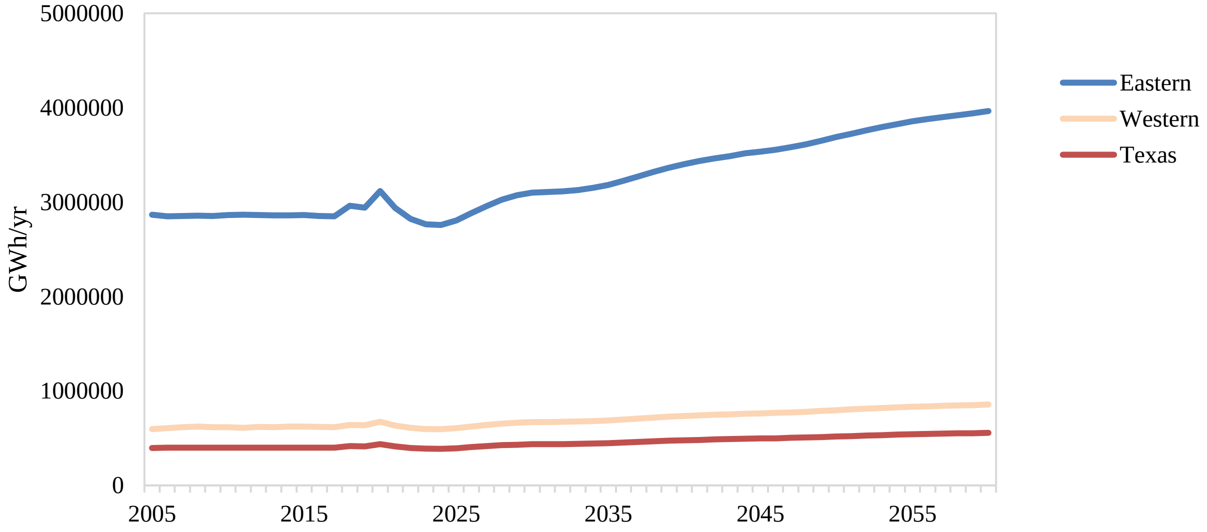

Fig. S9. U.S. power generation regions and output. (**a**). Distribution of the main electricity regions in the U.S. (**b**). Projected generation by region (GWh/yr^{-1}) (E3-US).

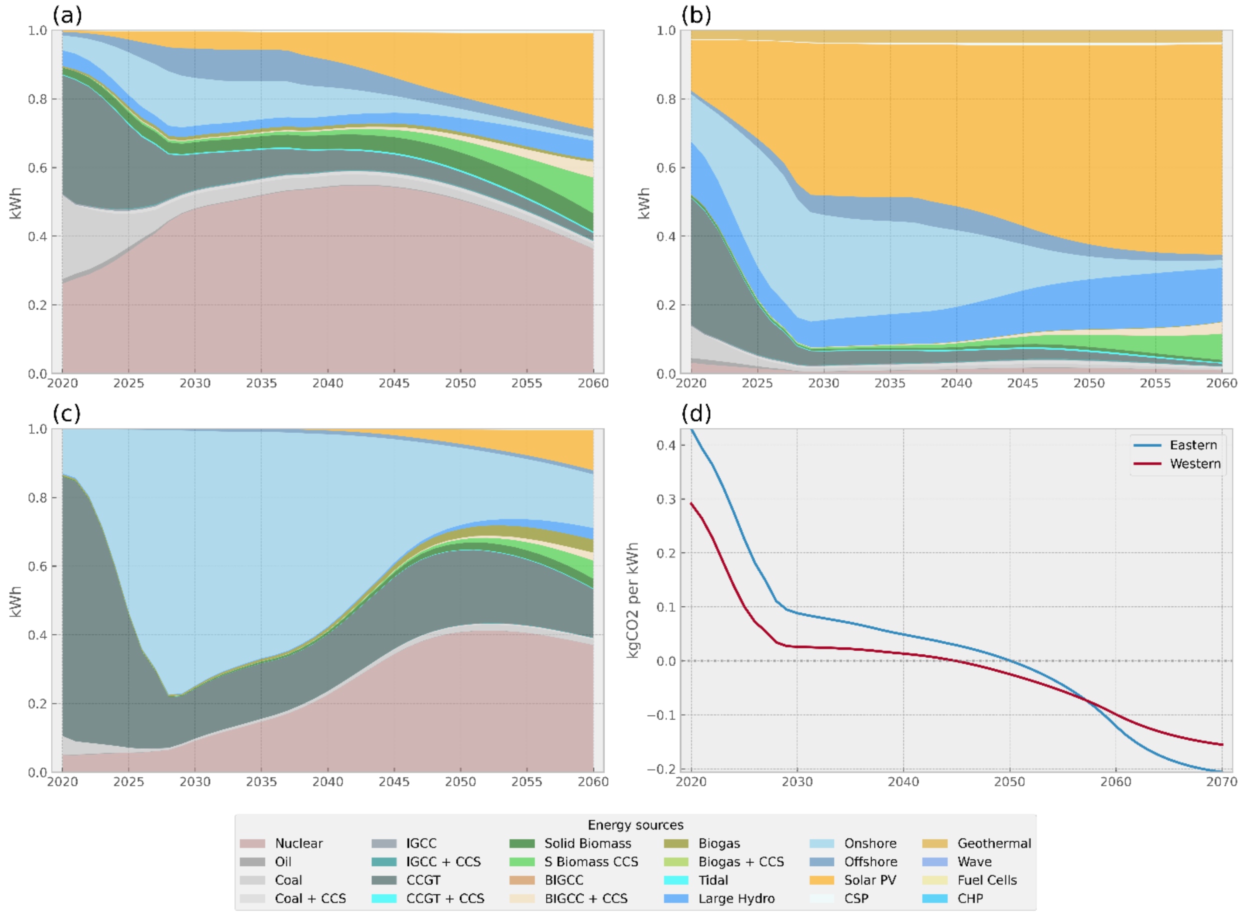

Fig. S10. Projected U.S. distribution of energy production by region. (**a**) Projected distribution of energy technologies to generate a kWh in the Eastern Region, (**b**) Western Region, and (**c**) Texas Region. The left axis indicates the contribution of energy technologies to generate a kWh. (**d**) average regional carbon emissions in kg of CO_2 /kWh (E3-US).

(a) 1 Gt yr^{-1} rock extraction by 2070 scenario

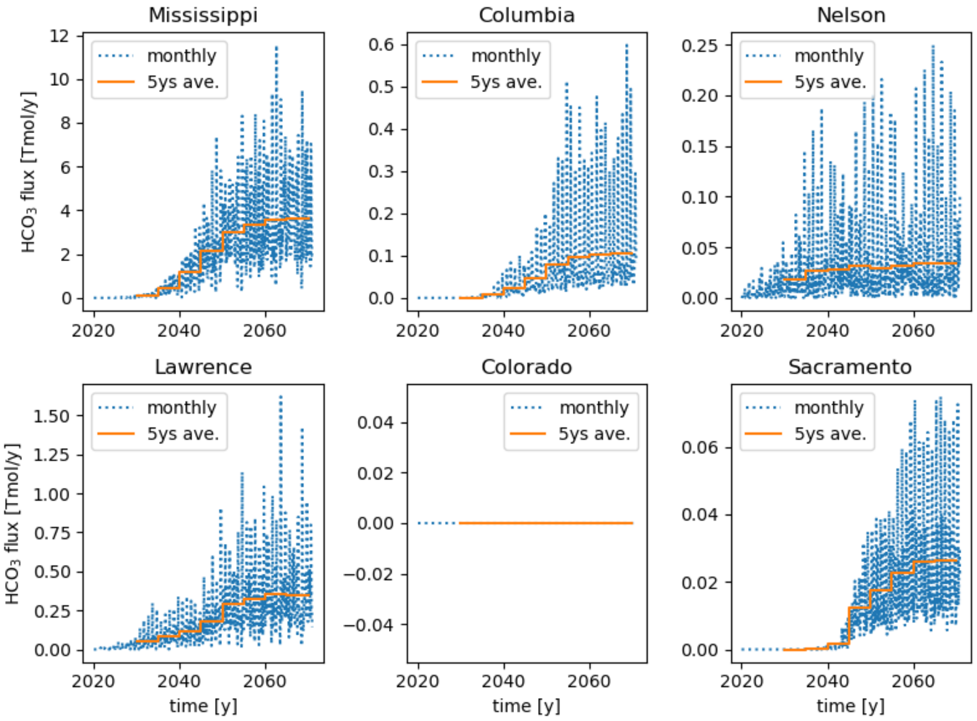

(**b**) 2 Gt yr^{-1} rock extraction by2070 scenario

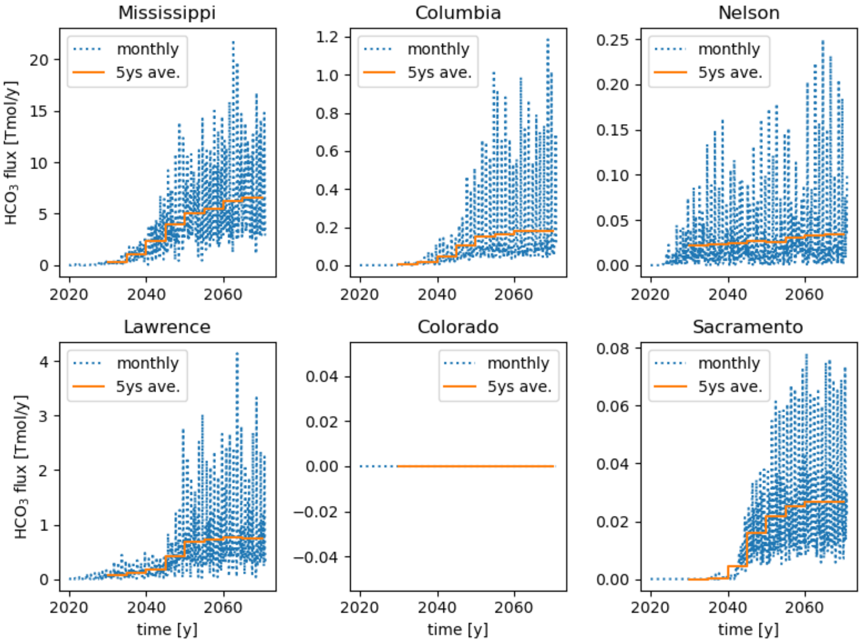

Fig. S11. Riverine HCO_3^- fluxes for six watersheds and both rock extraction scenarios

(a) 1 Gt yr^{-1} rock extraction by 2070 scenario

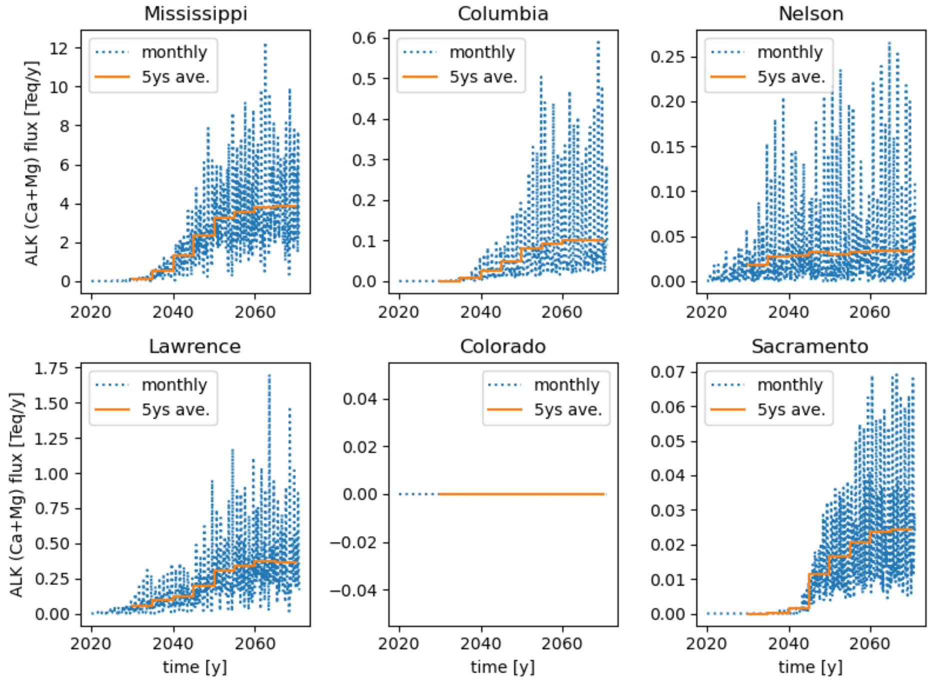

(b) 2 Gt yr^{-1} rock extraction by 2070 scenario

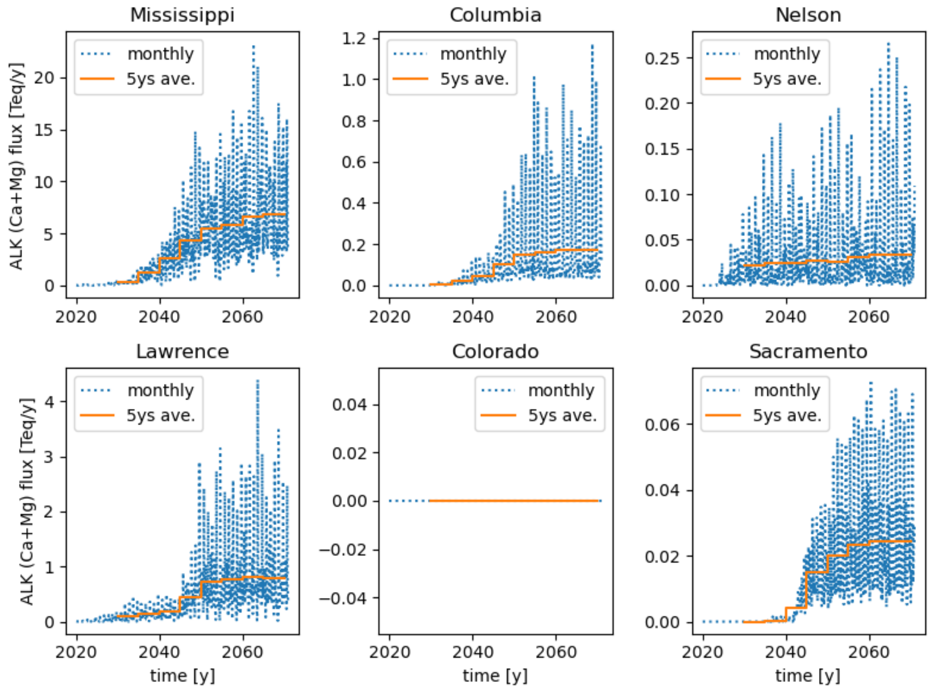

Fig. S12. Riverine alkalinity fluxes for six watersheds and both rock extraction scenarios

(a) 1 Gt yr^{-1} rock extraction by 2070 scenario

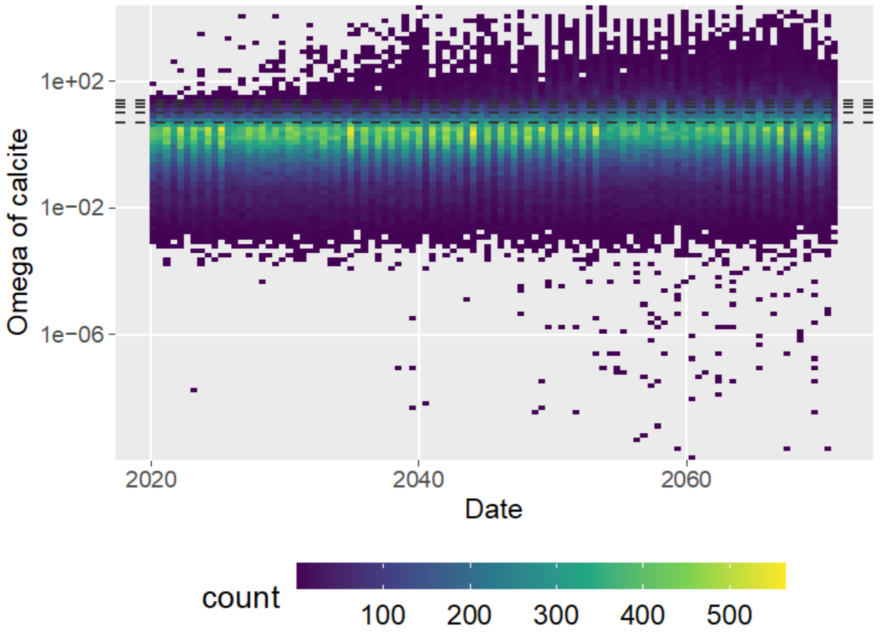

(**b**) 2 Gt yr^{-1} rock extraction by 2070 scenario

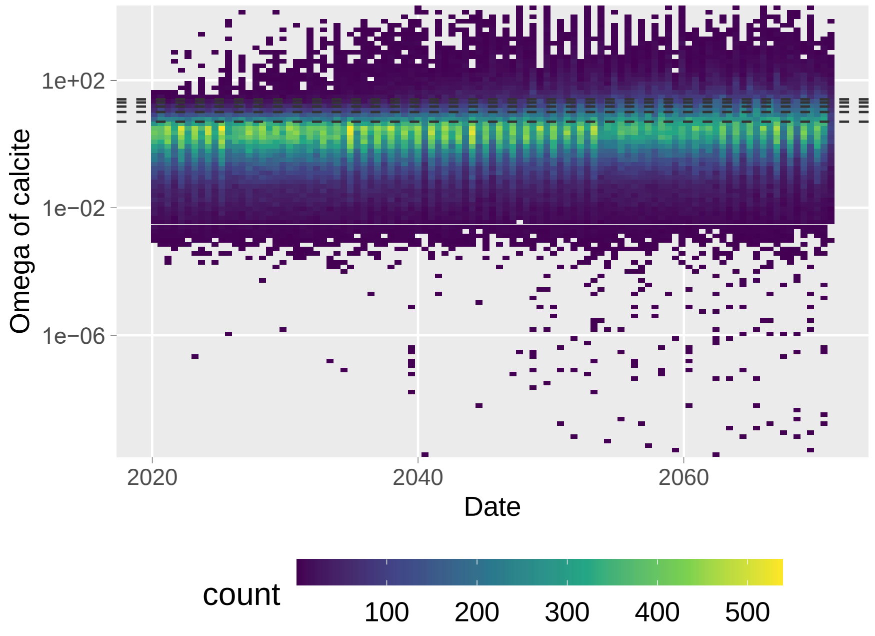

Fig. S13. Evolution of river carbonate saturation state for both EW scenarios.

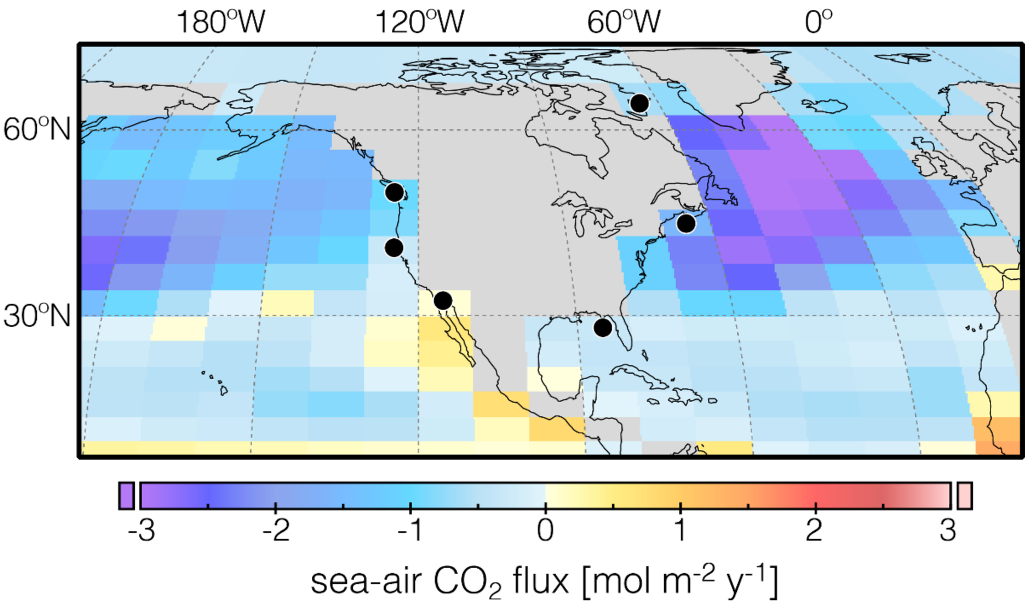

Fig. S14. Location of major river basin drainage sites for GENIE simulations.

SI References

1 Hollocher, K. *Calculation of a CIPW norm from a bulk chemical analysis*, <https://muse.union.edu/hollochk/files/2021/07/norm4.xlsx> (2011).

2 PetDB database *EarthChem Syntheses*, <https://earthchem.org/data-access/earthchem-syntheses/> (2021).

3 GEOROC database *Geochemistry of rocks of the oceans and continents*, <https://georoc.eu/> (2022).

4 Lehnert, K., Su, Y., Langmuir, C. H., Sarbas, B. & Nohl, U. A global geochemical database structure for rocks. *Geochemistry Geophysics Geosystems* **1**, 1012, doi:https://doi.org/10.1029/1999GC000026 (2000).

5 Lewis, A. L. *et al.* Effects of mineralogy, chemistry and physical properties of basalts on carbon capture potential and plant-nutrient element release via enhanced weathering. *Applied Geochemistry*, 105023, doi:10.1016/j.gca.2004.05.027 (2021).

6 Renforth, P. The potential of enhanced weathering in the UK. *International Journal of Greenhouse Gas Control* **10**, 229–243, doi:https://doi.org/10.1016/j.ijggc.2012.06.011 (2012).

7 Statista *U. S. crushed stone production*, <https://www.statista.com/.> (2023).

8 National Minerals Information Center USGS aggregates time series data by state, type, and end use. (https://www.usgs.gov/media/files/usgs-aggregates-time-series-data-state-type-and-end-use, 2021, accessed April 2023).

9 Wernet, G. *et al.* The ecoinvent database version 3 (part I): overview and methodology. *The International Journal of Life Cycle Assessment* **21**, 1218–1230, doi:https://doi.org/10.1007/s11367-016-1087-8 (2016).

10 Kantzas, E. P. *et al.* Substantial carbon drawdown potential from enhanced rock weathering in the United Kingdom. *Nature Geoscience* **15**, 382–389, doi:10.1038/s41561-022-00925-2 (2022).

11 Danabasoglu, G. *et al.* The Community Earth System Model Version 2 (CESM2). *Journal of Advances in Modeling Earth Systems* **12**, e2019MS001916, doi:https://doi.org/10.1029/2019MS001916 (2020).

12 Lawrence, D. M. *et al.* The Community Land Model Version 5: Description of New Features, Benchmarking, and Impact of Forcing Uncertainty. *Journal of Advances in Modeling Earth Systems* **11**, 4245–4287, doi:https://doi.org/10.1029/2018MS001583 (2019).

13 Lawrence, D. M. *et al.* The Land Use Model Intercomparison Project (LUMIP) contribution to CMIP6: rationale and experimental design. *Geoscientific Model Development* **9**, 2973–2998, doi:10.5194/gmd-9-2973-2016 (2016).

14 USDA. *Fertilizer use and price*, <https://www.ers.usda.gov/data-products/fertilizer-use-and-price/> (2019).

15 FAO. World fertilizer trends and outlook to 2022. (Rome, 2019).

16 Blanc-Betes, E. *et al.* In silico assessment of the potential of basalt amendments to reduce N_2O emissions from bioenergy crops. *Global Change Biology: Bioenergy* **13**, 224–241, doi:https://doi.org/10.1111/gcbb.12757 (2020).

17 Val Martin, M. *et al.* Improving nitrogen cycling in a land surface model (CLM5) to quantify soil N_2O, NO, and NH_3 emissions from enhanced rock weathering with

croplands. *Geoscientific Model Development* **16**, 5783–5801, doi:https://doi.org/10.5194/gmd-16-5783-2023 (2023).
18 Chiaravalloti, I., Theunissen, N. & Planavsky, N. J. Observed ammonia fluxes during maize production in mesocosms with basalt amendments. *ESS Open Archive*, doi:https://doi.org/10.22541/essoar.167751584.47065180/v1 (2023).
19 Beerling, D. J. *et al.* Potential for large-scale CO_2 removal via enhanced rock weathering with croplands. *Nature* **583**, 242–248, doi:10.1038/s41586-020-2448-9 (2020).
20 Palandri, J. L. & Kharaka, Y. K. A compilation of rate parameters of water-mineral interaction kinetics for application to geochemical modeling. *Open File Report 2004-1068*, 64 pages (2004).
21 Pohlmann, M. *et al.* Pore water chemistry reveals gradients in mineral transformation across a model basaltic hillslope. *Geochemistry Geophysics Geosystems* **17**, 2054–2069, doi:https://doi.org/10.1002/2016GC006270 (2016).
22 Navarre-Sitchler, A. & Brantley, S. Basalt weathering across scales. *Earth and Planetary Science Letters* **261**, 321–334, doi:https://doi.org/10.1016/j.epsl.2007.07.010 (2007).
23 Ramcharan, A. *et al.* Soil Property and Class Maps of the Conterminous United States at 100-Meter Spatial Resolution. *Soil Science Society of America Journal* **82**, 186–201, doi:https://doi.org/10.2136/sssaj2017.04.0122 (2018).
24 Banwart, S. A., Berg, A. & Beerling, D. J. Process-based modeling of silicate mineral weathering responses to increasing atmospheric CO_2 and climate change. *Global Biogeochemical Cycles* **23**, GB4013, doi:https://doi.org/10.1029/2008GB003243 (2009).
25 Nelson, P. N. & Su, N. Soil pH buffering capacity: a descriptive function and its application to some acidic tropical soils *Soil Research* **48**, 201–207, doi:https://doi.org/10.1071/SR09150 (2010).
26 Cerling, T. E. Carbon dioxide in the atmosphere; evidence from Cenozoic and Mesozoic Paleosols. *American Journal of Science* **291**, 377–400 (1991).
27 Jackson, R. B. *et al.* A global analysis of root distributions for terrestrial biomes. *Oecologia* **108**, 389–411, doi:10.1007/BF00333714 (1996).
28 Renforth, P. & Henderson, G. Assessing ocean alkalinity for carbon sequestration. **55**, 636–674, doi:https://doi.org/10.1002/2016RG000533 (2017).
29 Brown, T. J. *et al.* Underground mining of aggregates. Main report. 322 pp. (Camborne School of Mines, Penryn, UK, 2010).
30 Roussanaly, S. *et al.* Offshore power generation with carbon capture and storage to decarbonise mainland electricity and offshore oil and gas installations: A techno-economic analysis. *Applied Energy* **233**, 478–494, doi:https://doi.org/10.1016/j.apenergy.2018.10.020 (2019).
31 Woods, D. R. *Rules of thumb in engineering practice*. 479 pp. (Wiley-VCH Verlag GmbH & Col KG2A, 2007).
32 Gerdes, K., Summers, W. M. & Wimer, J. Quality Guidelines for Energy System Studies: Cost Estimation Methodology for NETL Assessments of Power Plant Performance. (National Energy Technology Laboratory (NETL), Pittsburgh, PA, United States, 2011).
33 Morrell, S. Predicting the overall specific energy requirement of crushing, high pressure grinding roll and tumbling mill circuits. *Minerals engineering* **22**, 544–549, doi:https://doi.org/10.1016/j.mineng.2009.01.005 (2009).
34 Mercure, J. F. *et al.* Macroeconomic impact of stranded fossil fuel assets. *Nature Climate Change* **8**, 588–593, doi:10.1038/s41558-018-0182-1 (2018).

35 Phadke, A. A., Khandekar, A., Abhyankar, N., Wooley, D. & Rajagopal, D. Why regional and long-haul trucks are primed for electrification now. (2021).
36 Liimatainen, H., van Vliet, O. & Aplyn, D. The potential of electric trucks – An international commodity-level analysis. *Applied Energy* **236**, 804-814, doi:https://doi.org/10.1016/j.apenergy.2018.12.017 (2019).
37 AEO2020. Annual Energy Outlook 2020 with projections to 2050. (U.S. Energy Information Administration (EIA), 2020).
38 BTS.GOV. (ed Bureau of Transportation Statistics) (2013).
39 IEA. The Future of Rail, Opportunities for energy and the environment. International Energy Agency. (2019).
40 SMMT. Truck specification for best operational efficiency, guide, department for transport. (2010).
41 MDS. Analysis of road and rail costs between coal mines and power stations, MDS TRANSMODAL LIMITED. (2012).
42 Leonardi, J., McKinnon, A. & Palmer, A. *Guidance on measuring and reporting greenhouse gas (GHG) emissions from freight transport operations*, <https://assets.publishing.service.gov.uk/media/5a7c2df4e5274a25a9140f9d/ghg-freight-guide.pdf> (2009).
43 Oscar Delgado, Felipe Rodríguez & Muncrief, R. Fuel efficiency technology in European heavy-duty vehicles: baseline and potential for the 2020–2030 time frame. The International Council of Clean Transportation. (2017).
44 Hartmann, J. & Moosdorf, N. The new global lithological map database GLiM: A representation of rock properties at the Earth surface. *Geochemistry, Geophysics, Geosystems* **13**, doi:10.1029/2012GC004370 (2012).
45 Maguire, D. J. in *Encyclopedia of GIS* (eds Shashi Shekhar & Hui Xiong) 25-31 (Springer US, 2008).
46 Sprung, M. J. *et al.* Freight facts and figures. (US Department of Transportation, https://www.bts.dot.gov/sites/bts.dot.gov/files/docs/FFF_2017_Full_June2018revision.pdf, 2018, accessed April 2023).
47 Chewpreecha, U., Pollitt, H. & Mercure, J.-F. in *Prospects and Policies for Global Sustainable Recovery: Promoting Environmental and Economic Sustainability* (eds Philip Arestis & Malcolm Sawyer) 215–263 (Springer International Publishing, 2023).
48 Holden, P. B. *et al.* Climate–carbon cycle uncertainties and the Paris Agreement. *Nature Climate Change* **8**, 609–613, doi:10.1038/s41558-018-0197-7 (2018).
49 Mercure, J.-F. *et al.* Environmental impact assessment for climate change policy with the simulation-based integrated assessment model E3ME-FTT-GENIE. *Energy strategy reviews* **20**, 195–208, doi:https://doi.org/10.1016/j.esr.2018.03.003 (2018).
50 Cambridge Econometrics E3ME Manual: Version 9.0. (https://www.e3me.com/what/e3me/, 2022).
51 Council of Economic Advisers & Office of Management and Budget. *White paper* 48 (Washington DC, 2023).
52 USGS. Water data for the nation. (United States Geological Survey, http://waterdata.usgs.gov/nwis/, 2016).
53 GRASS Development Team Geographic Resources Analysis Support System (GRASS GIS). (Open Source Geospatial Foundation, 2017).

54 Lehner, B., Verdin, K. & Jarvis, A. New global hydrography derived from spaceborne elevation data. *Transactions of the American Geophysical Union* **89**, 93–94 (2008).

55 Zeebe, R. E. & Wolf-Gladrow, D. *CO2 in seawater: equilibrium, kinetics, isotopes*. (Gulf Professional Publishing, 2001).

56 Gattuso, J.-P. *et al.* Seawater carbonate chemistry. (http://cran.r-project.org/package=seacarb, 2015).

57 Zhang, S. *et al.* River chemistry constraints on the carbon capture potential of surficial enhanced rock weathering. *Limnology and Oceanography* **67**, S148–S157, doi:10.1002/lno.12244 (2022).

58 Marsh, R., Müller, S., Yool, A. & Edwards, N. Incorporation of the C-GOLDSTEIN efficient climate model into the GENIE framework: "eb_go_gs" configurations of GENIE. *Geoscientific Model Development* **4**, 957–992, doi:10.5194/gmd-4-957-2011 (2011).

59 Reinhard, C. T. *et al.* Oceanic and atmospheric methane cycling in the cGENIE Earth system model – release v0.9.14. *Geoscientific Model Development* **13**, 5687–5706, doi:10.5194/gmd-13-5687-2020 (2020).

60 Edwards, N. R. & Marsh, R. Uncertainties due to transport-parameter sensitivity in an efficient 3-D ocean-climate model. *Climate Dynamics* **24**, 415–433, doi:10.1007/s00382-004-0508-8 (2005).

61 Ridgwell, A. *et al.* Marine geochemical data assimilation in an efficient Earth System Model of global biogeochemical cycling. *Biogeosciences* **4**, 87–104, doi:10.5194/bg-4-87-2007 (2007).

62 Kanzaki, Y., Planavsky, N. J. & Reinhard, C. T. New estimates of the storage permanence and ocean co-benefits of enhanced rock weathering. *PNAS Nexus* **2**, pgad059, doi:10.1093/pnasnexus/pgad059 (2023).

63 Meinshausen, M. *et al.* The RCP greenhouse gas concentrations and their extensions from 1765 to 2300. *Climatic Change* **109**, 213, doi:10.1007/s10584-011-0156-z (2011).

64 Emmons, L. K. *et al.* The chemistry mechanism in the Community Earth System Model Version 2 (CESM2). *Journal of Advances in Modeling Earth Systems* **12**, e2019MS001882, doi:https://doi.org/10.1029/2019MS001882 (2020).

65 Lu, Z. *et al.* Radiative forcing of nitrate aerosols from 1975 to 2010 as simulated by MOSAIC module in CESM2-MAM4. *Journal of Geophysical Research: Atmospheres* **126**, e2021JD034809, doi:https://doi.org/10.1029/2021JD034809 (2021).

66 Riahi, K. *et al.* The Shared Socioeconomic Pathways and their energy, land use, and greenhouse gas emissions implications: An overview. *Global environmental change* **42**, 153–168, doi:https://doi.org/10.1016/j.gloenvcha.2016.05.009 (2017).

67 Gelaro, R. *et al.* The Modern-Era Retrospective Analysis for Research and Applications, Version 2 (MERRA-2). *Journal of Climate* **30**, 5419–5454, doi:https://doi.org/10.1175/JCLI-D-16-0758.1 (2017).

68 Lamarque, J. F. *et al.* CAM-chem: description and evaluation of interactive atmospheric chemistry in the Community Earth System Model. *Geoscientific Model Development* **5**, 369–411, doi:10.5194/gmd-5-369-2012 (2012).

69 Schwantes, R. H. *et al.* Evaluating the impact of chemical complexity and horizontal resolution on tropospheric ozone over the conterminous US with a global variable resolution chemistry model. *Journal of Advances in Modeling Earth Systems* **14**, e2021MS002889, doi:https://doi.org/10.1029/2021MS002889 (2022).

70 Tai, A. P. K., Sadiq, M., Pang, J. Y. S., Yung, D. H. Y. & Feng, Z. Impacts of surface ozone pollution on global crop yields: comparing different ozone exposure metrics and incorporating co-effects of CO_2. *Frontiers in Sustainable Food Systems* **5**, 534616, doi:https://doi.org/10.3389/fsufs.2021.534616 (2021).
71 Val Martin, M., Heald, C. L. & Arnold, S. R. Coupling dry deposition to vegetation phenology in the Community Earth System Model: Implications for the simulation of surface O_3. *Geophysical Research Letters* **41**, 2988–2996, doi:https://doi.org/10.1002/2014GL059651 (2014).
72 USDA. Statistics by state. (US Department of Agriculture, 2021, accessed 21 April 2023).
73 FAO. Food and agriculture 2050 data portal version 2.0 BETA. (FAO, 2018, accessed 21 April 2023).
74 McGrath, J. M. *et al.* An analysis of ozone damage to historical maize and soybean yields in the United States. *Proceedings of the National Academy of Sciences* **112**, 14390–14395, doi:https://doi.org/10.1073/pnas.1509777112 (2015).
75 Van Dingenen, R. *et al.* The global impact of ozone on agricultural crop yields under current and future air quality legislation. *Atmospheric Environment* **43**, 604–618, doi:https://doi.org/10.1016/j.atmosenv.2008.10.033 (2009).
76 Lesser, V. M., Rawlings, J. O., Spruill, S. E. & Somerville, M. C. Ozone Effects on Agricultural Crops: Statistical Methodologies and Estimated Dose-Response Relationships. *Crop Science* **30**, 148–155, doi:https://doi.org/10.2135/cropsci1990.0011183X003000010033x (1990).
77 Adams, R. M., Glyer, J. D., Johnson, S. L. & McCarl, B. A. A Reassessment of the Economic Effects of Ozone on U.S. Agriculture. *Journal of the Air & Waste Management Association* **39**, 960–968, doi:https://doi.org/10.1080/08940630.1989.10466583 (1989).
78 Mills, G. *et al.* Closing the global ozone yield gap: Quantification and cobenefits for multistress tolerance. *Global Change Biology* **24**, 4869–4893, doi:https://doi.org/10.1111/gcb.14381 (2018).
