# Supplementary figures and images for: Transforming US agriculture for carbon removal with enhanced weathering
Source: Nature. 2025 Feb 5;638(8050):425–34. doi: 10.1038/s41586-024-08429-2 (PMC11821523; doi:10.1038/s41586-024-08429-2)

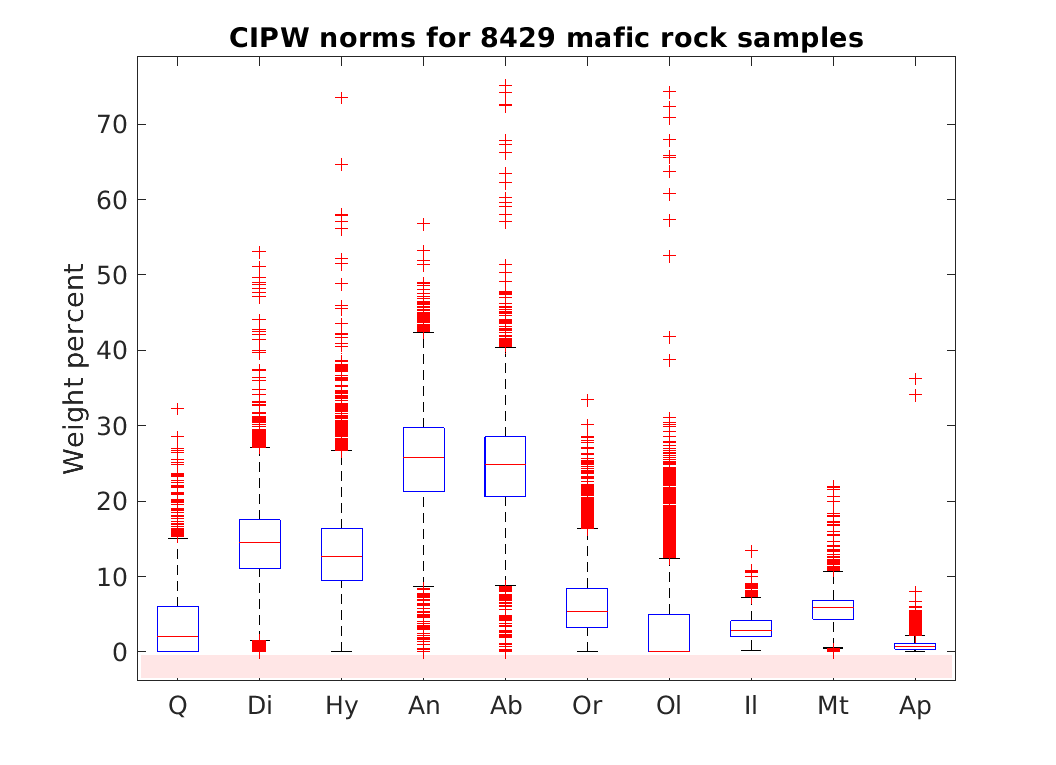

Supplement: Supplementary file 2 — geochemistrydata_normcode. [file 41586_2024_8429_MOESM2_ESM.gz › tarballfiles/CIPWnormsfor8429maficrocksamples_SiO2max_57pct_fullsamponly_boxplots_14-Nov-2023.png]

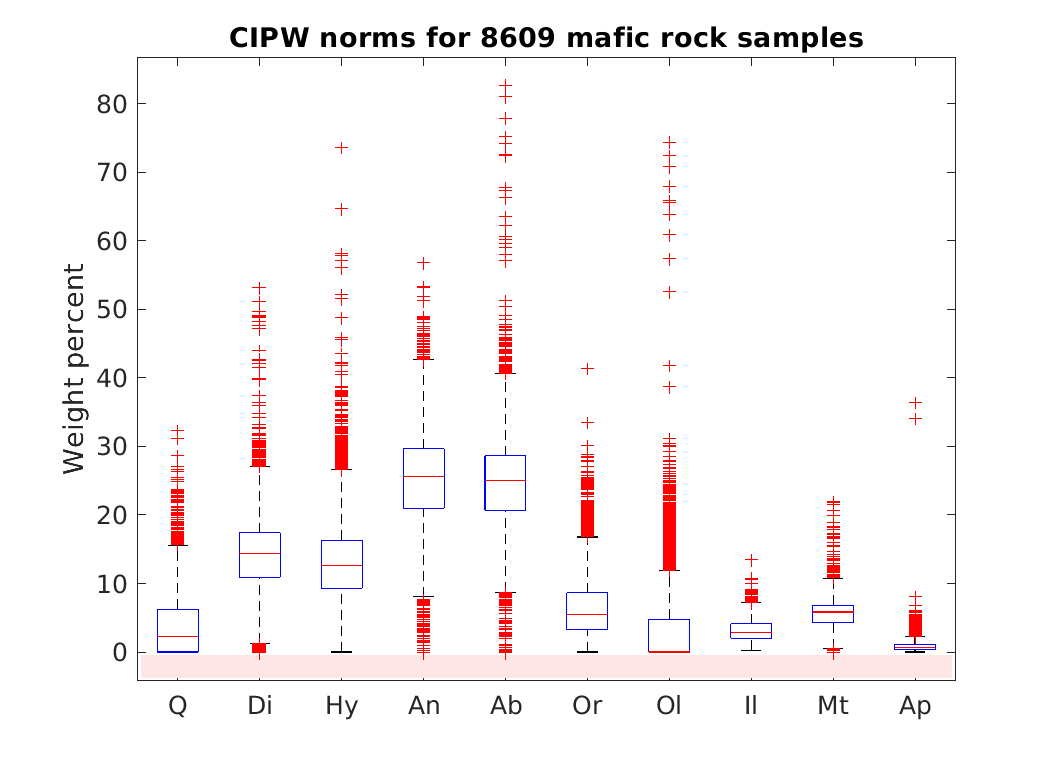

Supplement: Supplementary file 2 — geochemistrydata_normcode. [file 41586_2024_8429_MOESM2_ESM.gz › tarballfiles/CIPWnormsfor8609maficrocksamples_SiO2max_63pct_fullsamponly_boxplots_14-Nov-2023.png]

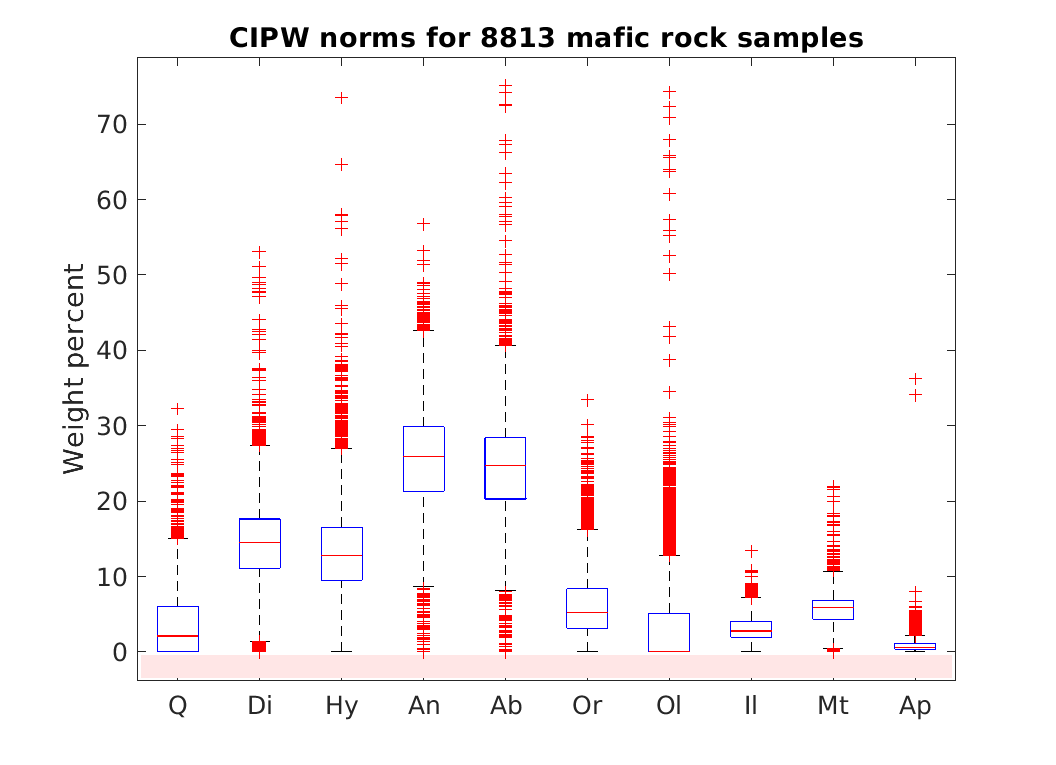

Supplement: Supplementary file 2 — geochemistrydata_normcode. [file 41586_2024_8429_MOESM2_ESM.gz › tarballfiles/CIPWnormsfor8813maficrocksamples_boxplots_05-Nov-2023.png]

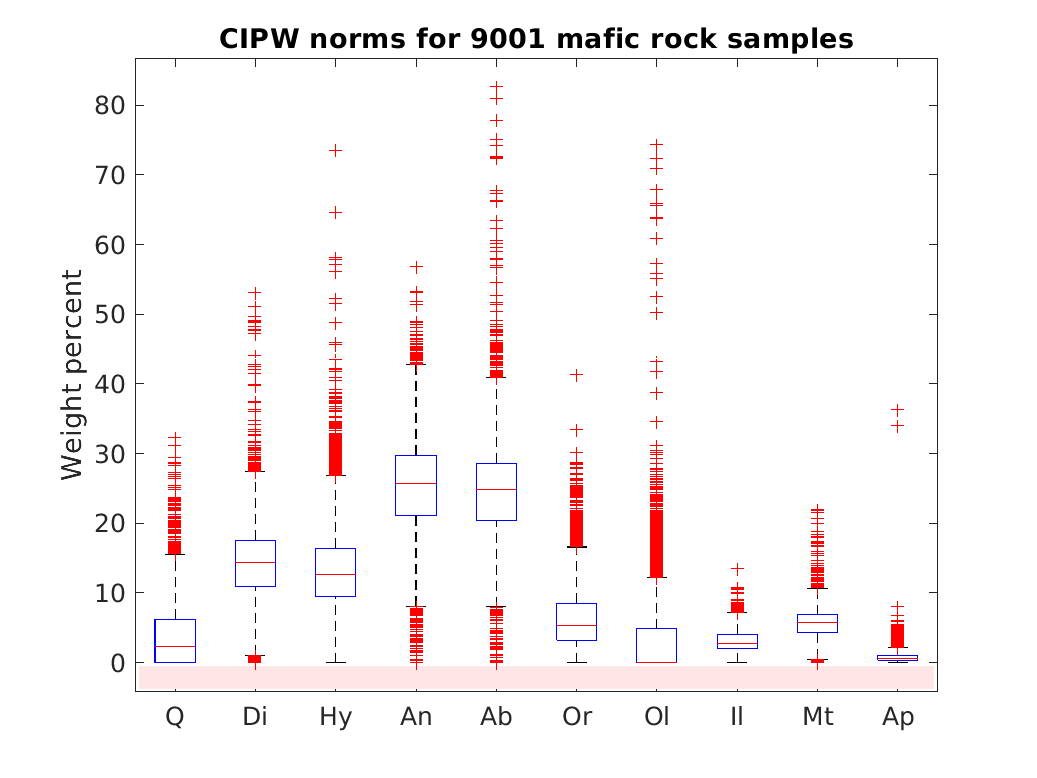

Supplement: Supplementary file 2 — geochemistrydata_normcode. [file 41586_2024_8429_MOESM2_ESM.gz › tarballfiles/CIPWnormsfor9001maficrocksamples_boxplots_05-Nov-2023.png]

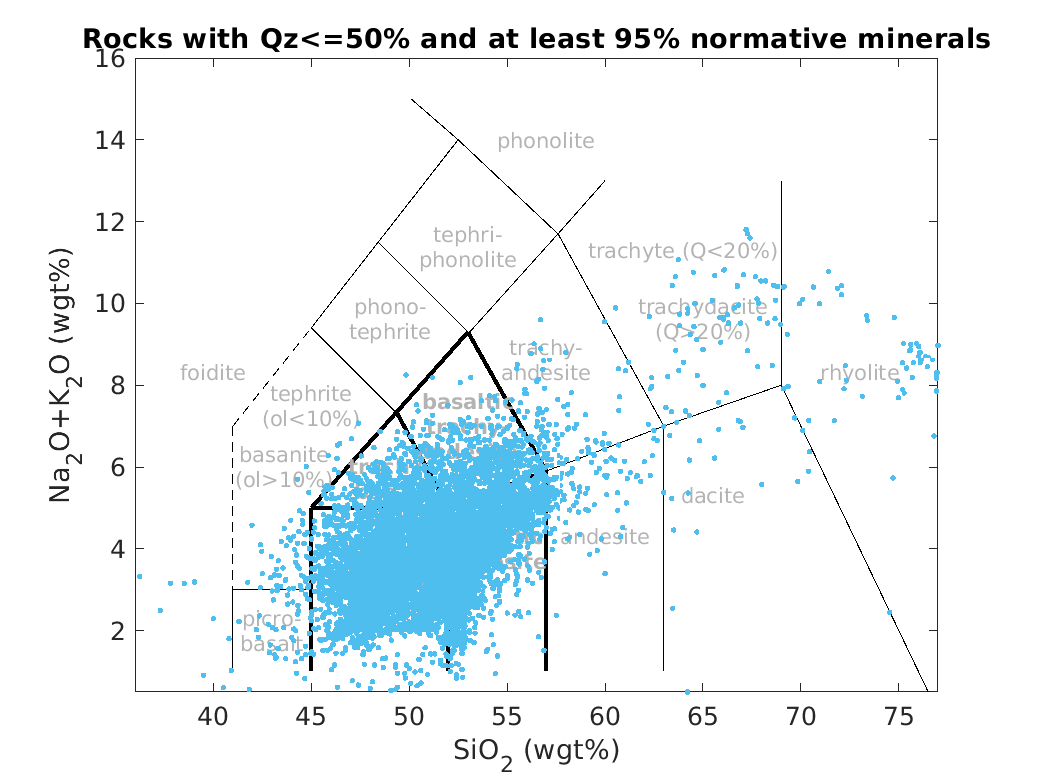

Supplement: Supplementary file 2 — geochemistrydata_normcode. [file 41586_2024_8429_MOESM2_ESM.gz › tarballfiles/TAS_normQcutoff_50pct_05-Nov-2023.png]

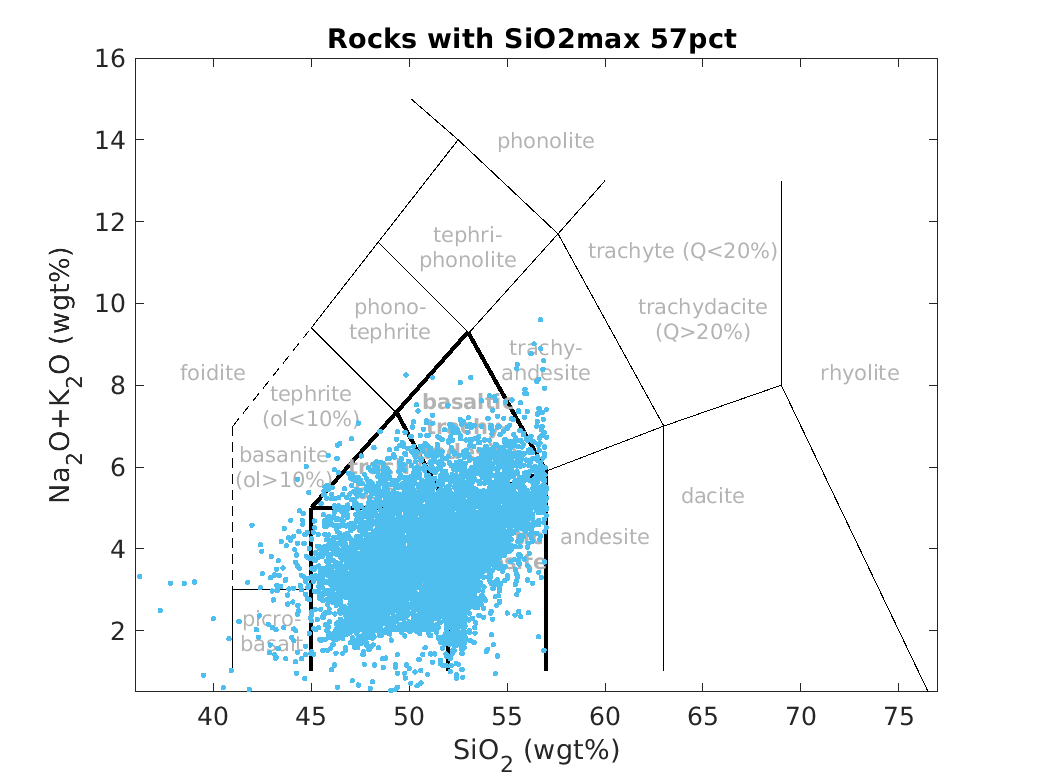

Supplement: Supplementary file 2 — geochemistrydata_normcode. [file 41586_2024_8429_MOESM2_ESM.gz › tarballfiles/TAS_SiO2max_57pct_05-Nov-2023.png]

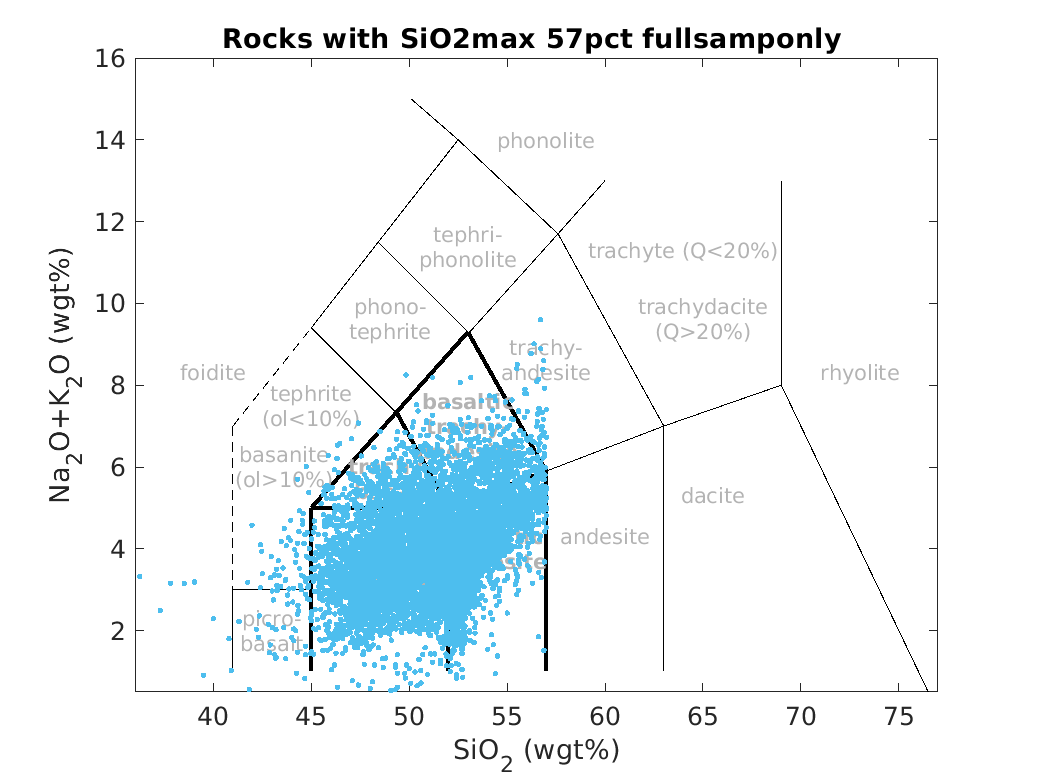

Supplement: Supplementary file 2 — geochemistrydata_normcode. [file 41586_2024_8429_MOESM2_ESM.gz › tarballfiles/TAS_SiO2max_57pct_fullsamponly_14-Nov-2023.png]

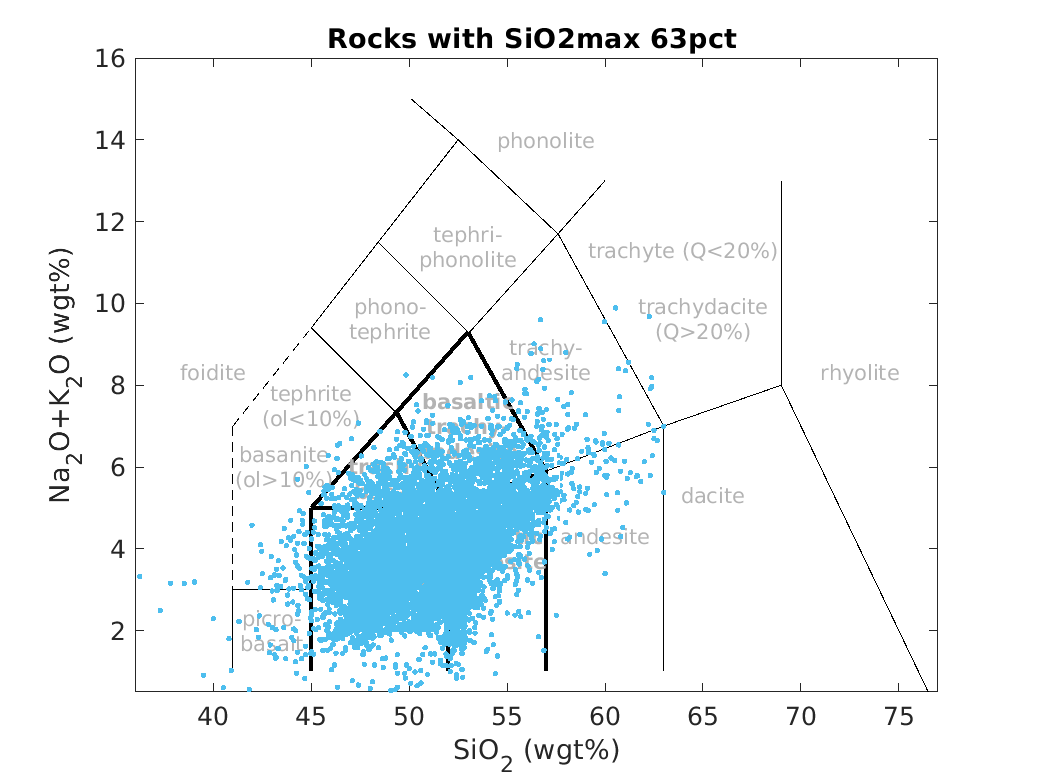

Supplement: Supplementary file 2 — geochemistrydata_normcode. [file 41586_2024_8429_MOESM2_ESM.gz › tarballfiles/TAS_SiO2max_63pct_05-Nov-2023.png]

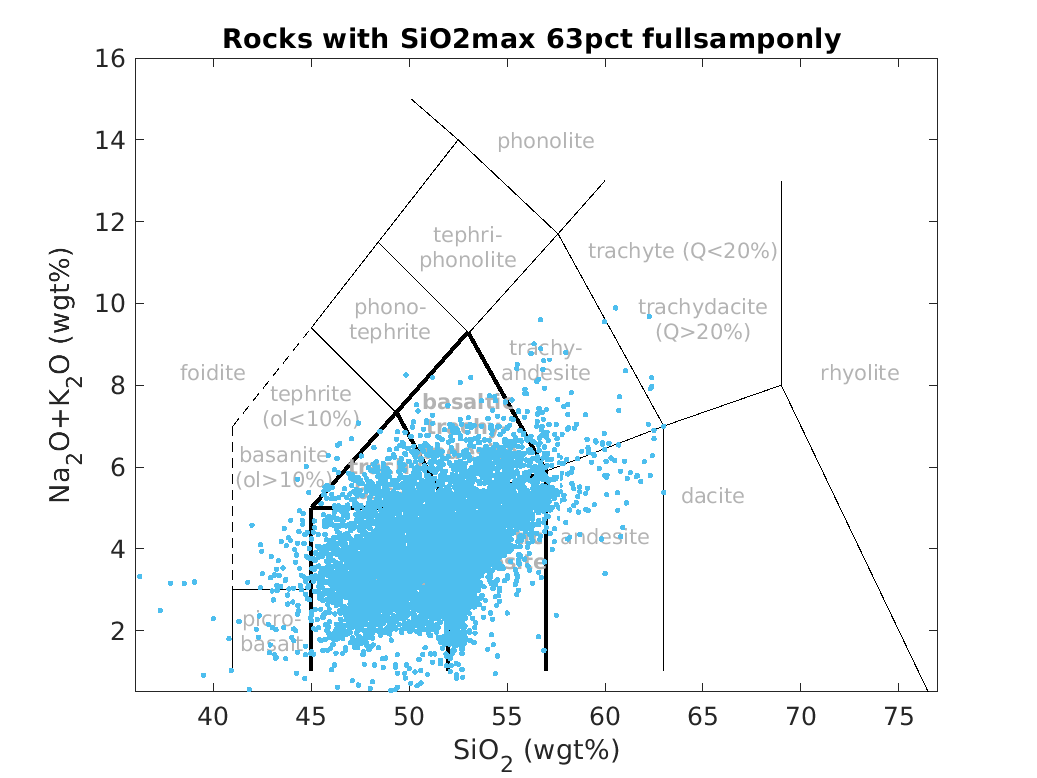

Supplement: Supplementary file 2 — geochemistrydata_normcode. [file 41586_2024_8429_MOESM2_ESM.gz › tarballfiles/TAS_SiO2max_63pct_fullsamponly_14-Nov-2023.png]

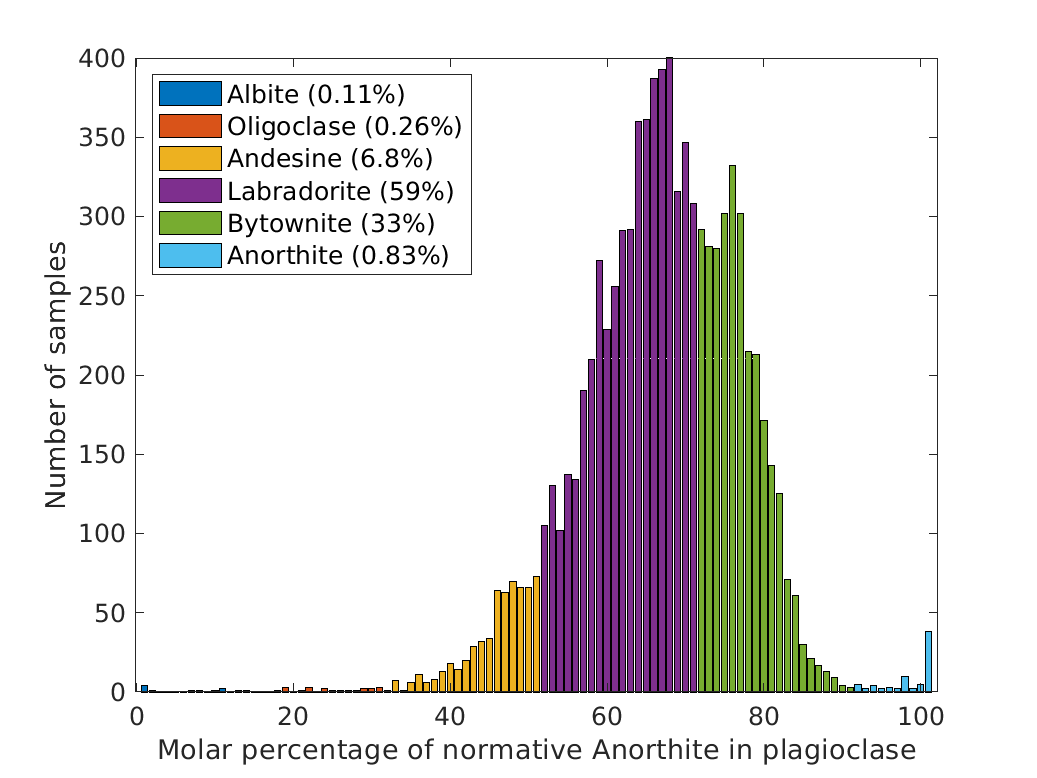

Supplement: Supplementary file 2 — geochemistrydata_normcode. [file 41586_2024_8429_MOESM2_ESM.gz › tarballfiles/NAm_hist_Anx_mafic_01-Dec-2023.png]

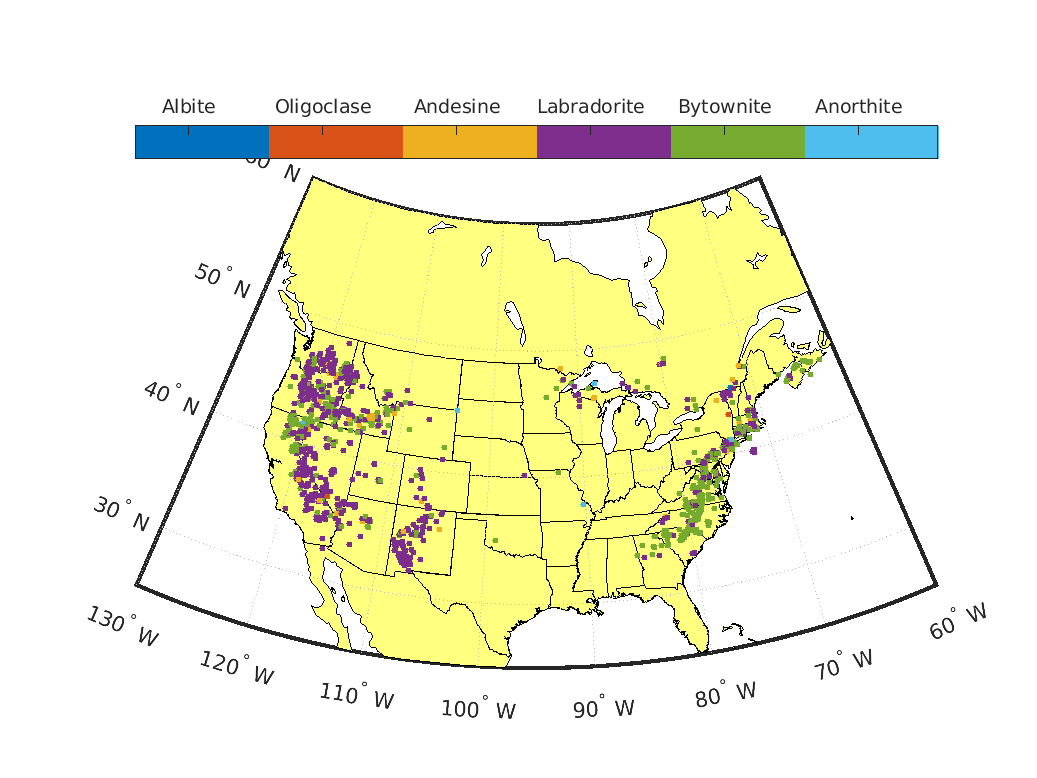

Supplement: Supplementary file 2 — geochemistrydata_normcode. [file 41586_2024_8429_MOESM2_ESM.gz › tarballfiles/NAm_map_Anx_mafic_01-Dec-2023.png]

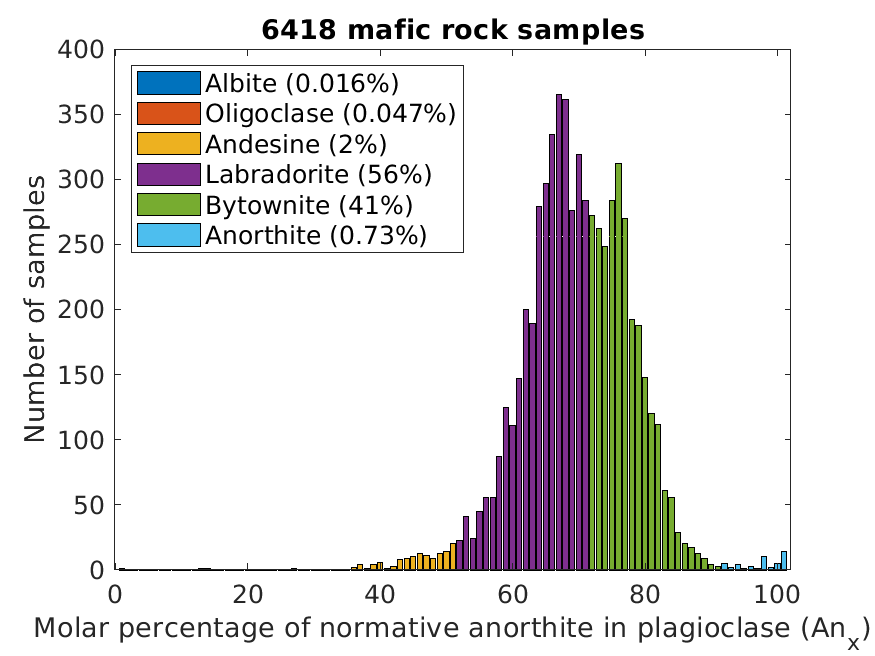

Supplement: Supplementary file 2 — geochemistrydata_normcode. [file 41586_2024_8429_MOESM2_ESM.gz › tarballfiles/CIPWnormsfor6418maficrocksamples_SiO2max_57pct_fullsamponly_removecalcite_Anxhist_04-Mar-2024.png]

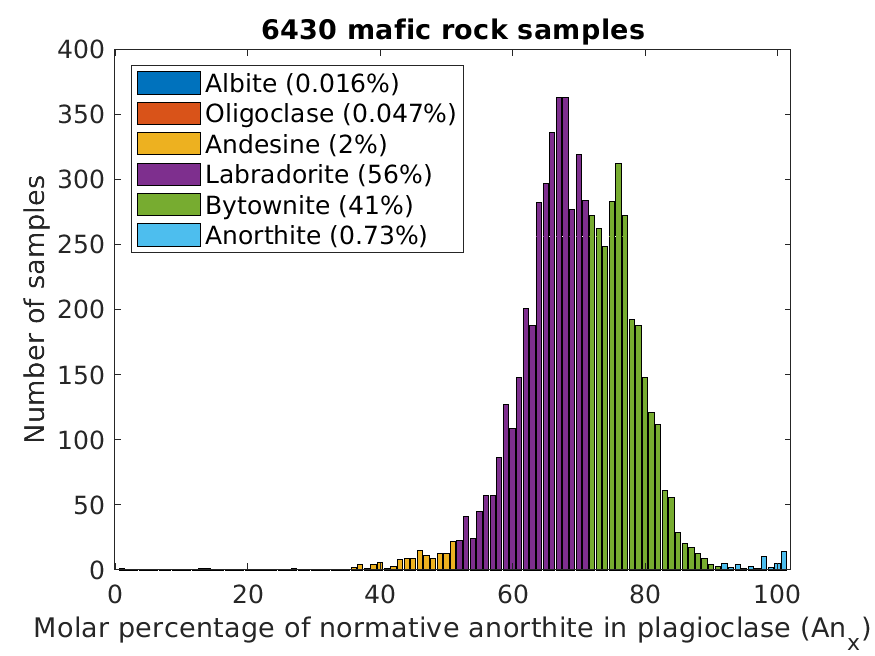

Supplement: Supplementary file 2 — geochemistrydata_normcode. [file 41586_2024_8429_MOESM2_ESM.gz › tarballfiles/CIPWnormsfor6430maficrocksamples_SiO2max_57pct_fullsamponly_Anxhist_04-Mar-2024.png]

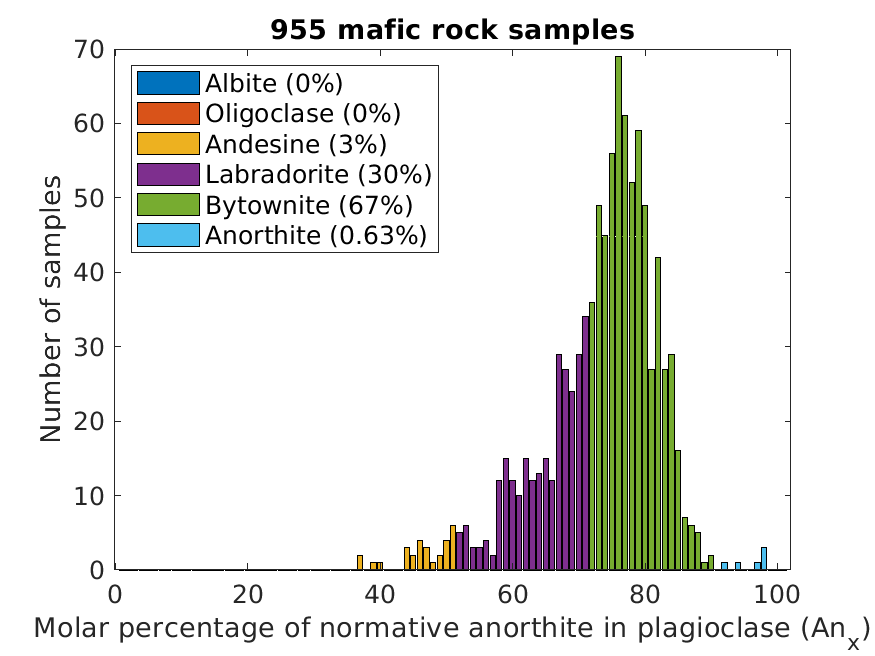

Supplement: Supplementary file 2 — geochemistrydata_normcode. [file 41586_2024_8429_MOESM2_ESM.gz › tarballfiles/CIPWnormsfor955maficrocksamples_SiO2max_57pct_fullsampco2_removecalcite_Anxhist_04-Mar-2024.png]

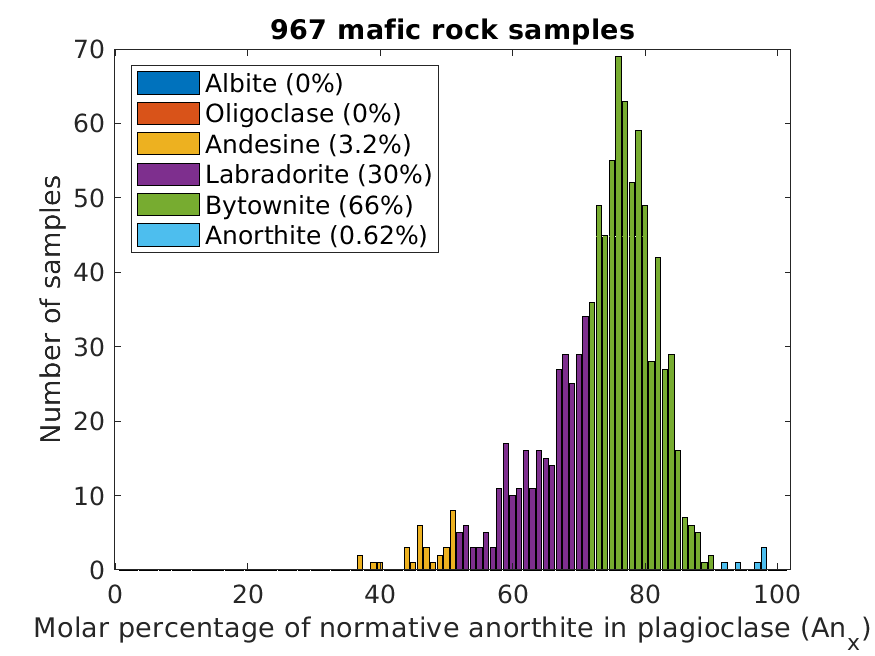

Supplement: Supplementary file 2 — geochemistrydata_normcode. [file 41586_2024_8429_MOESM2_ESM.gz › tarballfiles/CIPWnormsfor967maficrocksamples_SiO2max_57pct_fullsampco2_Anxhist_04-Mar-2024.png]

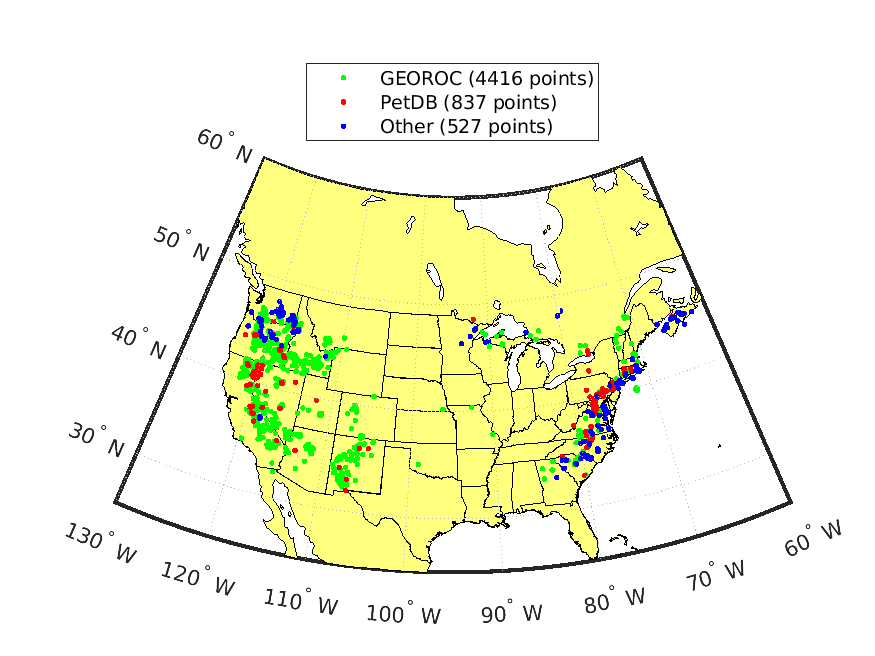

Supplement: Supplementary file 2 — geochemistrydata_normcode. [file 41586_2024_8429_MOESM2_ESM.gz › tarballfiles/NAm_map_oxides_mafic_04-Mar-2024.png]

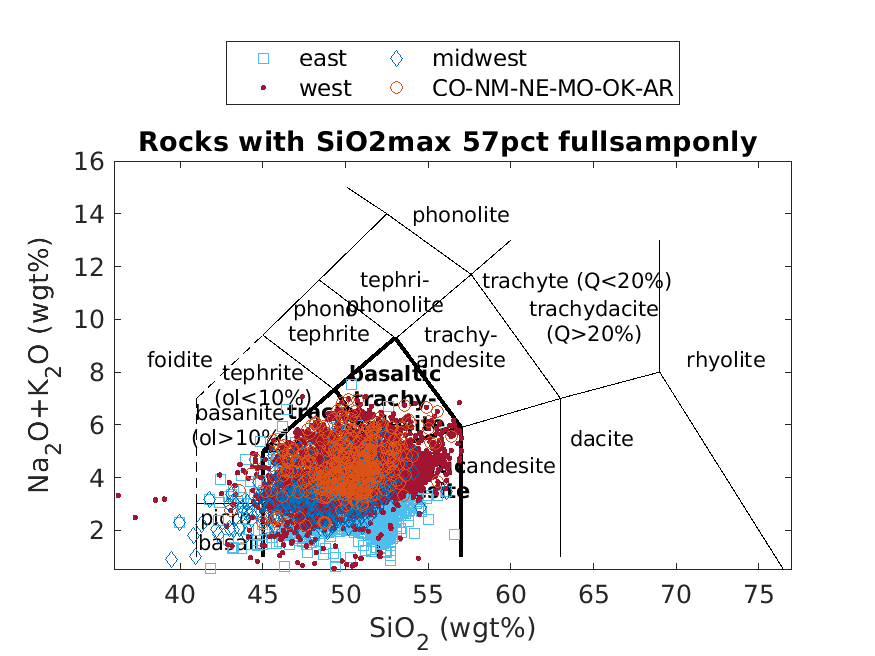

Supplement: Supplementary file 2 — geochemistrydata_normcode. [file 41586_2024_8429_MOESM2_ESM.gz › tarballfiles/TAS_maficrocks_04-Mar-2024.png]

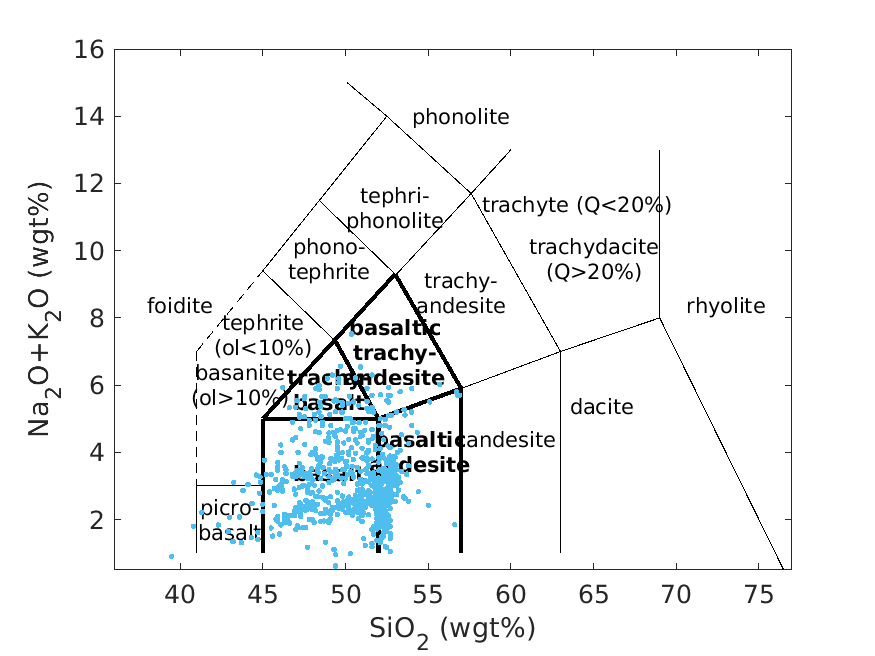

Supplement: Supplementary file 2 — geochemistrydata_normcode. [file 41586_2024_8429_MOESM2_ESM.gz › tarballfiles/TAS_SiO2max_57pct_fullsampco2_04-Mar-2024.png]

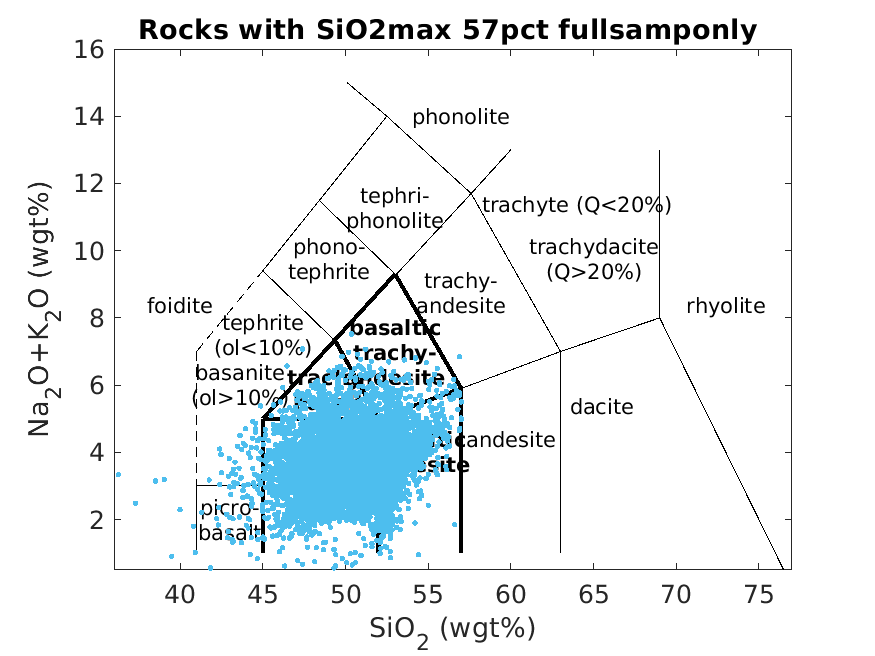

Supplement: Supplementary file 2 — geochemistrydata_normcode. [file 41586_2024_8429_MOESM2_ESM.gz › tarballfiles/TAS_SiO2max_57pct_fullsamponly_04-Mar-2024.png]

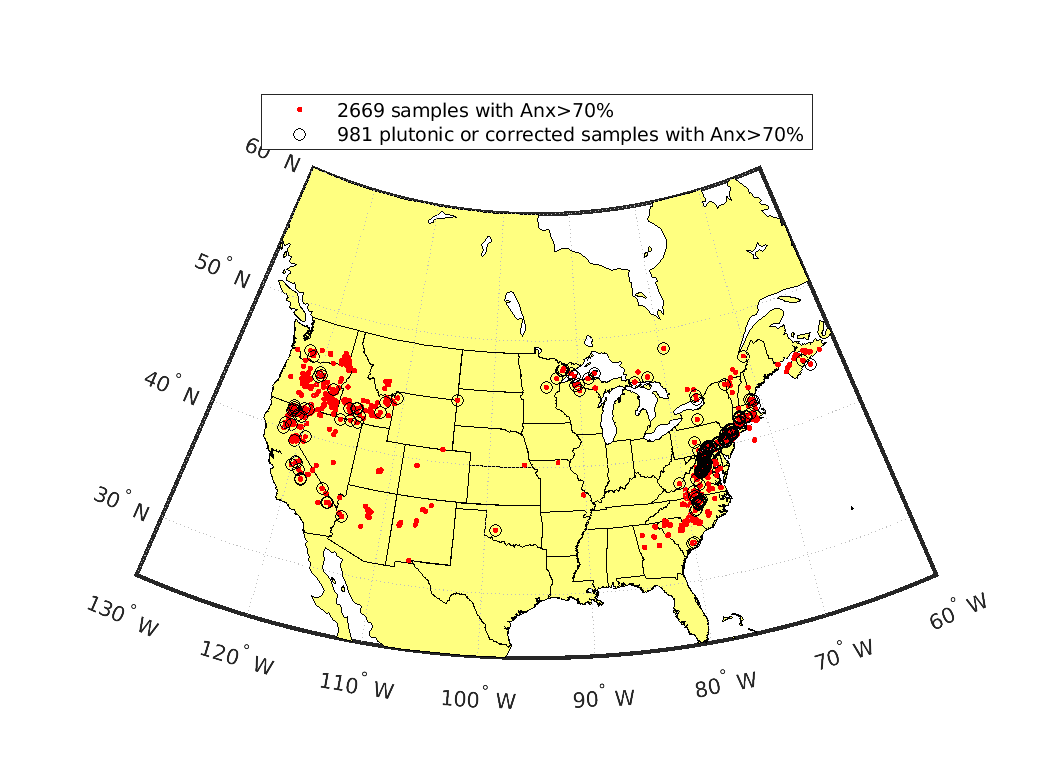

Supplement: Supplementary file 2 — geochemistrydata_normcode. [file 41586_2024_8429_MOESM2_ESM.gz › tarballfiles/map_USAstudy_2669_calcicsamples_08-Mar-2024.png]
